# Integration of Genome-Wide SNP Data and Gene-Expression Profiles Reveals Six Novel Loci and Regulatory Mechanisms for Amino Acids and Acylcarnitines in Whole Blood

**DOI:** 10.1371/journal.pgen.1005510

**Published:** 2015-09-24

**Authors:** Ralph Burkhardt, Holger Kirsten, Frank Beutner, Lesca M. Holdt, Arnd Gross, Andrej Teren, Anke Tönjes, Susen Becker, Knut Krohn, Peter Kovacs, Michael Stumvoll, Daniel Teupser, Joachim Thiery, Uta Ceglarek, Markus Scholz

**Affiliations:** 1 LIFE Leipzig Research Center for Civilization Diseases, University of Leipzig, Leipzig Germany; 2 Institute of Laboratory Medicine, Clinical Chemistry and Molecular Diagnostics, University Hospital Leipzig, Leipzig, Germany; 3 Institute for Medical Informatics, Statistics and Epidemiology, University of Leipzig, Leipzig, Germany; 4 Department for Cell Therapy, Fraunhofer Institute for Cell Therapy and Immunology, Leipzig, Germany; 5 Heart Center Leipzig, Leipzig, Germany; 6 Institute for Laboratory Medicine, Ludwig-Maximilians University Munich, Munich, Germany; 7 Medical Department, Clinic for Endocrinology and Nephrology, University of Leipzig, Leipzig, Germany; 8 Interdisciplinary Centre for Clinical Research, University of Leipzig, Leipzig, Germany; 9 Integrated Research and Treatment Center Adiposity Diseases, University of Leipzig, Leipzig Germany; Wellcome Trust Sanger Institute, United Kingdom

## Abstract

Profiling amino acids and acylcarnitines in whole blood spots is a powerful tool in the laboratory diagnosis of several inborn errors of metabolism. Emerging data suggests that altered blood levels of amino acids and acylcarnitines are also associated with common metabolic diseases in adults. Thus, the identification of common genetic determinants for blood metabolites might shed light on pathways contributing to human physiology and common diseases. We applied a targeted mass-spectrometry-based method to analyze whole blood concentrations of 96 amino acids, acylcarnitines and pathway associated metabolite ratios in a Central European cohort of 2,107 adults and performed genome-wide association (GWA) to identify genetic modifiers of metabolite concentrations. We discovered and replicated six novel loci associated with blood levels of total acylcarnitine, arginine (both on chromosome 6; rs12210538, rs17657775), propionylcarnitine (chromosome 10; rs12779637), 2-hydroxyisovalerylcarnitine (chromosome 21; rs1571700), stearoylcarnitine (chromosome 1; rs3811444), and aspartic acid traits (chromosome 8; rs750472). Based on an integrative analysis of expression quantitative trait loci in blood mononuclear cells and correlations between gene expressions and metabolite levels, we provide evidence for putative causative genes: *SLC22A16* for total acylcarnitines, *ARG1* for arginine, *HLCS* for 2-hydroxyisovalerylcarnitine, *JAM3* for stearoylcarnitine via a trans-effect at chromosome 1, and *PPP1R16A* for aspartic acid traits. Further, we report replication and provide additional functional evidence for ten loci that have previously been published for metabolites measured in plasma, serum or urine.

In conclusion, our integrative analysis of SNP, gene-expression and metabolite data points to novel genetic factors that may be involved in the regulation of human metabolism. At several loci, we provide evidence for metabolite regulation via gene-expression and observed overlaps with GWAS loci for common diseases. These results form a strong rationale for subsequent functional and disease-related studies.

## Author Summary

Human metabolite levels differ between individuals due to environmental and genetic factors. In the present work, we analyzed whole blood levels of amino acids and acylcarnitines, reflecting disease relevant metabolic pathways, in a cohort of 2,107 individuals. We then performed a genome wide association analysis to discover genetic variants influencing metabolism. Thereby, we discovered six novel regions in the genome and confirmed ten regions previously found to be associated with metabolites in plasma, serum or urine. Subsequently, we analyzed whether these variants regulate gene-expression in peripheral mononuclear cells and at several loci we identified novel causal relations between SNPs, gene-expression and metabolite levels. These findings help explaining the functional mechanisms by which associated genetic variants regulate metabolism. Finally, several SNPs associated with blood metabolites in our study overlap with previously identified loci for human diseases (e.g. kidney disease), suggesting a shared genetic basis or pathomechanisms involving metabolic alterations. The identified loci are strong candidates for future functional studies directed to understand human metabolism and pathogenesis of related diseases.

## Introduction

High-throughput metabolomics experiments using mass spectrometry platforms are becoming an integral part of clinical and systems biology research. Profiling of amino acids and acylcarnitine species in dried whole blood samples of newborns is used worldwide in neonatal screening programs to identify rare inborn errors of metabolism [1]. These diseases are generally caused by rare mutations, leading to loss of function of an enzyme that catalyzes the biochemical reaction of the respective trait. Recently, many of the amino acid and fatty acid metabolites utilized in newborn screening were also implicated in common complex diseases of adults such as cardiovascular disease, insulin resistance and obesity. Exemplarily, obesity is accompanied by an increase in circulating levels of multiple amino acids, including branched chain amino acids [2,3], and in type 2 diabetics, altered levels of acylcarnitines were described [4,5]. Amino acids and acylcarnitines show substantial inter-individual variation [6] and a strong genetic contribution to their blood concentrations has been reported [7]. Thus, the integration of genetic and metabolic profiling holds the promise for providing novel insights into the regulation of metabolic homeostasis in health and disease.

Indeed, recent studies have identified common genetic variants associated with a variety of circulating metabolites in serum, plasma or urine using different analytical platforms (LC-MS/MS, NMR) [8–24]. However, the complexity of the metabolome cannot be captured by a single technology. Since differences in metabolite abundance have been described between plasma and whole blood [25], we hypothesized that additional genetic determinants affecting the blood metabolome are yet to be discovered.

Thus, we performed an integrated study combining genetics, gene expression and metabolom data (see S1 Fig for the study design). We applied a targeted LC-MS/MS method to measure the abundance of amino acids and acylcarnitines in dried whole blood spots of 2,107 individuals and performed genome-wide association analysis. Top findings were replicated in a second independent European Caucasian cohort of 923 Sorbs. Further, going beyond plain genetic associations, we integrated analyses of mRNA levels in leukocytes to establish causal links between genetic variations, gene-expression levels and metabolites. Finally, we explored whether SNP-metabolite associations identified in our study overlap with previously identified genetic loci for other complex traits or diseases.

## Results

### Discovery GWAS

**Fig 1:**
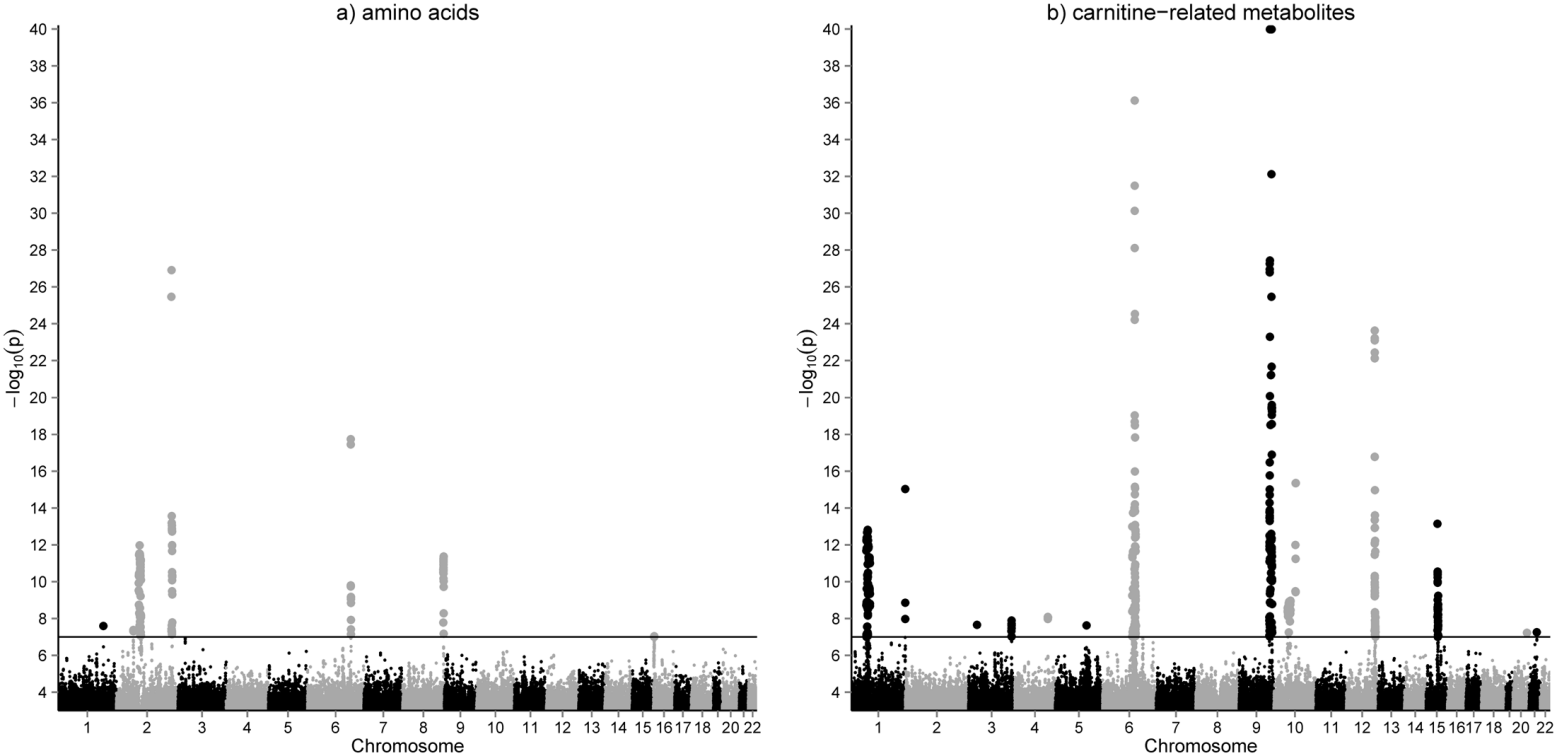
GWAS results for amino acids (a) and acylcarnitines (b) in whole blood. Manhattan plots of the genome-wide association analysis for metabolic phenotypes in 2,107 individuals of the LIFE-Heart cohort. Results are presented separately for 36 acylcarnitines (including free and total carnitine) and 26 amino acids. Results for metabolite ratios are omitted. The horizontal line represents a p-value = 1.0x10^-7^, which was the cutoff used for inclusion of identified associations in the replication state.

Quantitative concentrations of 26 amino acids, 36 acylcarnitines and 34 metabolite ratios were determined in dried whole blood spots of 2,107 participants of the LIFE Leipzig Heart Study using LC-MS/MS. Metabolites and their ratios reflect metabolic function of various biochemical pathways e.g. urea cycle, branched chain amino acid metabolism or cellular fatty acid oxidation (see S1 Table for complete list of phenotypes and their categories). We performed a genome wide association study (2,619,023 SNPs) for whole blood metabolites and identified 2,261 SNP-metabolite associations (119 after pruning) with p-values <10^-7^. These associations comprise 42 metabolites (including 19 ratios) and 866 SNPs (54 lead-SNPs after pruning) at 25 unique genomic locations (Fig 1, S2 Table). QQ-plots and regional association plots for all loci demonstrating valid quality control are presented in the supplemental material (S2 and S3 Figs).

### Replication analysis

**Fig 2:**
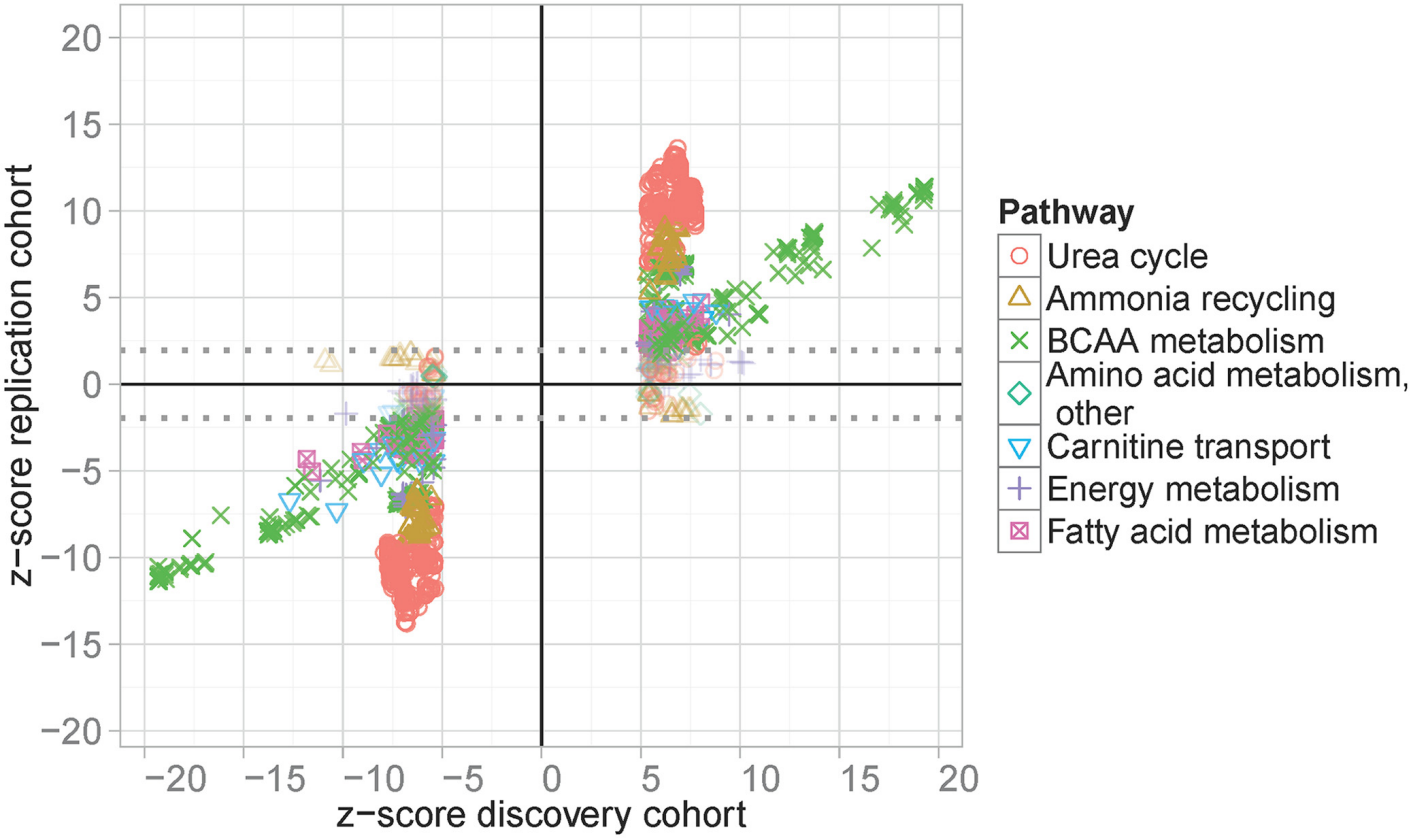
Results of replication analysis. GWAS top-hits of the LIFE Leipzig Heart study were compared with corresponding results in the Sorbs study. Top-hits were selected applying a p-value cut-off of p<1.0x10^-7^, which leads to the gap of z-scores at the x-axis. Associations below and above the dotted lines are considered as replicated controlling the false discovery rate at 5%. Colors and symbols correspond to physiologically related metabolites.

Next, replication of top SNPs was sought in an independent cohort of 923 individuals from the Sorb study, where genome-wide SNP and metabolite datasets were available. Good proxies (r^2^>0.8) for replication analysis in the Sorbs were available for 858 (99.1%) of our 866 top-SNPs, covering 21 of the 25 identified loci and comprising 2,227 associations (well-imputed proxies were not available for the loci at 1q32.3, 3p24.1, 5p15.2, 20q13.2, see S3 Table for complete results). We observed identical directions of effects for 2,133 (95.8%) combinations of SNPs and metabolites in the replication cohort, resulting in a replication rate of 88.3%, when applying a FDR (false discovery rate) of 5% (Fig 2). Replicated lead-SNPs were distributed over 14 of the 21 genomic loci eligible for replication analysis (Table 1; see S3 Table for results of non-replicated loci). In addition, we considered associations at locus #4 (2q34) with glycine and locus #14 (12q24.31) with C4 as validated results, since these loci were already reported in other GWAS for serum metabolites [8,9,13–15]. Moreover, non-lead-SNPs at 12q24.31 were replicated in the Sorbs at FDR 5% level. None of the other non-replicated loci or loci without proxies in the Sorb study achieved a p-value <10^−8^ in our initial GWAS.

**Table 1:** Results of SNP-metabolite association analyses.

Lo-cus	Lead-SNPs^1^	Cytogen. pos.	Phys. pos.	Nearby genes^2^	Associated Metabolites^3^	Beta (SE) LIFE-HEART	p-value LIFE-HEART	Beta (SE) Sorbs	p-value Sorbs	Published metabolite associations
**#1**	**rs17587071**, rs1303870	1p31.1	76 Mb	*ACADM* (0), *RABGGTB* (27), *SNORD45C* (28)	**C8**, C10, C6	-0.021 (0.0028)	1.487e-13	-0.011 (0.0031)	3.431e-04	Acetylcarnitine / hexanoylcarnitine, C12 / C10, C12 / C8 [13,14,20,21]
**#2**	**rs3811444**	1q44	246 Mb	*TRIM58* (0), *OR2W3* (0), *OR11L1* (34)	**C18**, C18:1, C16, Q3:(C16+C18:1)/C2, Q4:C0/(C16+C18)	0.051 (0.0063)	9.138e-16	0.063 (0.013)	2.22e-06	
**#3**	**rs6546838**, rs12620074	2p13.1	74 Mb	*ALMS1* (0), *EGR4* (160), *FBXO41* (180)	**Q15:Arg/Gly**, Q14:Arg/Orn, Q13:Arg/Cit, Arg	-0.0074 (0.00092)	2.079e-15	-0.0083 (0.00083)	2.385e-22	Myoinositol / N-acetylornithine, N-acetylornithine, N-acetylated compound(s) [8,13,14,16]
**#4**	**rs715**, rs4673545, rs13386028, rs10932350, rs7684	2q34	211 Mb	*CPS1* (0), *LOC29034* (59), *LANCL1* (200)	**Gly**, Q27:Sarc/Gly, Q15:Arg/Gly	-0.069 (0.0062)	1.232e-27	0.014 (0.011)	1.75e-01	Glycine, glycine / histidine, glycine / PC ae C38:2 [8,9,13–15,20]
**#5**	**rs4074110**	3q27.1	184 Mb	*MCCC1* (4.3), *DCUN1D1* (30), *ATP11B* (89)	**C5OH+HMG**	-0.024 (0.0042)	1.255e-08	-0.024 (0.0079)	2.003e-03	Hydroxyisovaleroylcarnitine [14]
**#6**	**rs11737481**	4q32.1	160 Mb	*ETFDH* (0), *PPID* (9), *C4orf46* (28)	**C10**	0.023 (0.0039)	8.068e-09	0.018 (0.0045)	9.602e-05	C14:1-OH / C10, decanoylcarnitine / palmitate (16:0), octanoylcarnitine / X-13435 [13,14,20]
**#7**	**rs162890**	5q31.1	132 Mb	*SLC22A4* (6.5), *PDLIM4* (15), *P4HA2* (60)	**AC-total**	0.047 (0.0084)	2.312e-08	0.063 (0.014)	1.1e-05	Propionylcarnitine [14]
**#8**	**rs12210538**, rs6939019, rs7763591, rs2428192, rs12205108	6q21	111 Mb	*SLC22A16* (0), *DDO* (23), *C6orf186* (81)	**AC-total**, C18:1, C16, C2, Q19:(Leu|Ile)/C3, C3, C0, Q11:Ala/C2, C182, C18, Q21:(C5OH+HMG)/(Leu|Ile), C5OH+HMG, C16:1, C20:3, C14, Q20:C5/(Leu|Ile), C4	-0.1 (0.0081)	7.397e-37	-0.12 (0.018)	2.003e-11	
**#9**	**rs17657775**	6q23.2	132 Mb	*ARG1* (0), *MED23* (0), *ENPP3* (50)	**Arg**, Q14:Arg/Orn, Q15:Arg/Gly, Q13:Arg/Cit	0.0561 (0.007)	2.606e-15^4^	0.0085 (0.0036)	1.934e-02^4^	
**#10**	**rs750472**	8q24.3	146 Mb	*FOXH1* (0), *KIFC2* (2), *CYHR1* (10)	**Q12:Ala/Asp**, Asp, Q34:Asp/C2, Q16:Asp/Cit	0.069 (0.0098)	2.39e-12	0.14 (0.02)	1.412e-11	
**#11**	**rs746872**, rs7874044, rs17452603, rs10760593, rs12686182, rs3124505, rs11789753, rs11999428	9q34.11	131 Mb	*CRAT* (0), *PPP2R4* (8.2), *DOLPP1* (12)	**MMA**, Q38:MMA/C3, C5OH+HMG, Q21:(C5OH+HMG)/(Leu|Ile)	-0.098 (0.0049)	4.214e-83	-0.089 (0.0078)	1.45e-28	C-glycosyltryptophan / succinylcarnitine [14]
**#12**	**rs12779637**	10q11.21	45 Mb	*MARCH8* (0), *ANUBL1* (30), *ALOX5* (140)	**C3**, C2	-0.13 (0.021)	1.04e-09	-0.12 (0.033)	4.46e-04	
**#13**	**rs1171614**	10q21.2	61 Mb	*SLC16A9* (0), *CCDC6* (79), *ANK3* (320)	**C0**, Q33:C0/(AC-total)	-0.12 (0.014)	4.268e-16	-0.1 (0.027)	1.428e-04	Carnitine, carnitine / X-12798 [13–15,20]
**#14**	**rs2066938**, rs12822898	12q24.31	120 Mb	*UNC119B* (0), *ACADS* (3), *MLEC* (21)	**C4**, Q35:C5/C4	0.046 (0.0044)	2.323e-24	0.0096 (0.0081)	2.333e-01	Butyrylcarnitine, butyrylcarnitine / propionylcarnitine [13,14,20,21]
**#15**	**rs12440281**, rs7162825, rs12442826, rs7183733	15q22.2	61 Mb	*TPM1* (18), *LACTB* (32), *RPS27L* (63)	**MMA**	0.081 (0.011)	6.986e-14	0.04 (0.018)	2.69e-02	succinylcarnitine [13,14]
**#16**	**rs1571700**	21q22.13	37 Mb	*HLCS* (0), *DSCR6* (42), *PIGP* (100)	**C5OH+HMG**	-0.02 (0.0037)	5.38e-08	-0.023 (0.0072)	1.173e-03	

Table includes all validated loci of our analysis. Validation is based on either successful replications in the Sorbs or by additional published evidence. The latter applies for two loci (#4 and #14) where association of lead-SNPs did not replicate in the Sorbs cohort. For each locus, nearby genes, independently associated SNPs, associated metabolites and statistics for the strongest association between them are shown (Beta estimators, corresponding standard errors and p-values). We also present the results of replication analysis and published evidence. Six loci with no corresponding published genetic variants were considered as “novel”.

^1^SNP with strongest association in the discovery cohort is presented in bold;

^2^Distance of SNPs to genes in kB in parentheses;

^3^Metabolite with strongest association in the discovery cohort is presented in bold. p-value Sorbs: best p-value of SNPs in Sorbs corresponding to the lead-SNP and metabolite of discovery cohort,

^4^Replication was successful for ratio Q14:Arg/Orn, only, hence, we report here on association with Q14:Arg/Orn

In total, our study led to the identification of 16 unique, validated loci for 36 whole blood metabolites (Table 1). At six of the 16 loci we identified associations for blood metabolites for the first time i.e. these loci represent novel findings of our study. Also, we successfully validated ten loci previously reported for serum, plasma, and urine metabolites (Table 1 and S4 Table). At three of these loci, associated metabolites were different from those previously reported. In detail, at locus #3 (2p13.1) we detected associations with Arg and related metabolite ratios, whereas earlier associations were reported for plasma N-acetylornithine and related compounds [8,13,14,16]. Further, at loci #11 (9q34.11) and #15 (15q22.2), we identified associations with methylmalonyl-carnitine, whereas earlier studies reported associations involving the isobaric compound succinyl-carnitine [13,14].

### eQTL analysis

To investigate if associated variants have gene regulatory effects, we analyzed our validated lead-SNPs for correlations with gene expression in peripheral blood mononuclear cells (PBMC). Transcriptome data (28,295 eligible transcripts) was available for 2,112 subjects of the LIFE Leipzig Heart study. At an FDR of 5%, 132 eQTLs were identified for 38 of the 45 validated lead-SNPs, affecting the expression of 69 transcripts. Explained variances of eQTLs ranged between 0.4% (corresponding p-value = 3.9x10^-3^) and 28.0% (corresponding p-value = 8.0x10^-153^, S5 Table).

**Fig 3:**
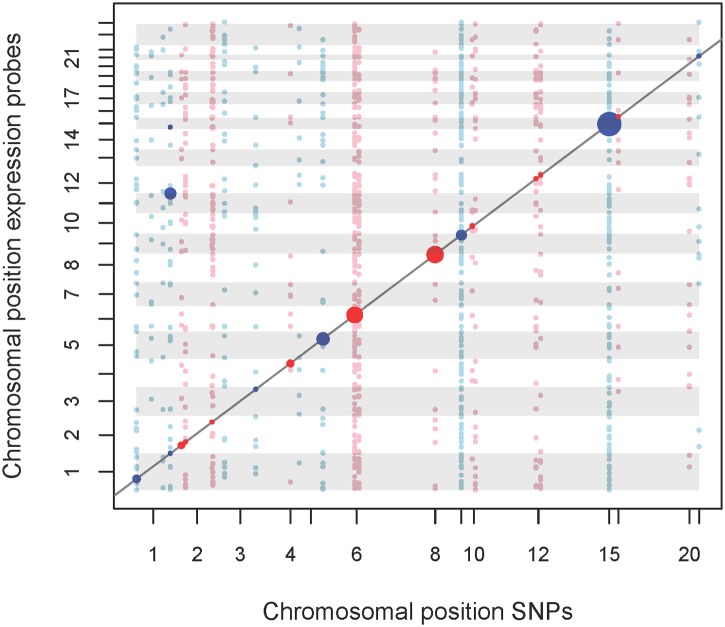
eQTL map of mQTL loci. We analysed the top-SNPs of our mQTL analysis regarding association with gene-expression levels. A total of 54 top-SNPs were correlated with 28,295 probe expressions. Expression probes of auto- and gonosomes were analysed, while SNPs were restricted to autosomes. X-axis represents physical position of SNPs. Y-axis represents the physical position of the start of the regulated transcript. Points located on the diagonal line relate to cis-effects, while other points relate to trans-effects. Associations with FDR = 5% are highlighted. Trans-eQTLs with p-values ≤ 0.001 are also shown. Size of points represents the strength of association. Colors of points and gray shadings indicate distinct chromosomes. An interactive html version of this map allowing exploration of the results is provided as supplemental S7 Fig.

We observed eQTLs at 14 of the 16 validated loci, including the six novel loci identified in our study (Fig 3 and S7 Fig, Table 2). All 14 loci included lead-SNPs with cis-regulatory effects on gene expression. In addition, novel loci #2 (1q44) and #12 (19q11), as well as reported locus #14 (12q24) also included trans-regulated eQTLs. The trans-eQTLs at locus #2 (1q44) regulating *JAM3* expression were inter-chromosomal and particularly strong, explaining about 13.0% of variance (Fig 3 and S7 Fig, Table 2).

**Table 2:** Results of eQTL analysis of validated loci.

Locus	Cytogen. pos.	Lead-SNPs^1^	Cis-regulated genes^2^	Trans-regulated genes^2^	Beta (SE)	p-value	q-value^3^
**#1**	1p31.1	rs17587071*, rs1303870	***ACADM***, *RABGGTB*		0.065 (0.004)	1.3e-45	2.2e-43
**#2**	1q44	rs3811444*	*SMYD3*, *OR2W3*	***JAM3***, *C15ORF54*	-0.23 (0.013)	4.9e-66	7.6e-60
**#3**	2p13.1	rs6546838*, rs12620074	***ALMS1***, *NAT8B*, *STAMBP*, *TPRKB*, *LOC200420*		0.028 (0.004)	1.3e-13	5.7e-12
**#5**	3q27.1	rs4074110*	***MCCC1***		0.023 (0.004)	1.4e-09	4.8e-08
**#6**	4q32.1	rs11737481*	***ETFDH***, *HS*.*415576*, *TMEM144*		-0.067 (0.005)	4.1e-41	6.2e-39
**#7**	5q31.1	rs162890*	***SLC22A5***, *SLC22A4*, *CDC42SE2*, *RAD50*, *P4HA2*		0.11 (0.006)	1.9e-74	4.8e-72
**#8**	6q21	rs7763591*, rs2428192, rs12205108, rs6939019, **rs12210538**	***SLC22A16***, *RPF2*, *DDO*, *CDC2L6*, *AMD1*		0.21 (0.009)	4.9e-98	2.5e-95
**#9**	6q23.2	rs17657775*	***MED23***, *ARG1*		0.048 (0.01)	2.7e-06	6.2e-05
**#10**	8q24.3	rs750472*	***PPP1R16A***, *LRRC14*, *KIFC2*, *RPL8*, *ZNF34*, *MFSD3*, *CYHR1*, *VPS28*, *LOC642859*, *COMMD5*		0.11 (0.005)	3.4e-102	2.6e-99
**#11**	9q34.11	rs746872*, rs7874044, rs12686182, rs3124505, rs10760593, rs17452603, rs11789753, rs11999428	***PPP2R4***, *SH3GLB2*, *CRAT*, *HS*.*148844*, *ENDOG*, *CCBL1*, *TBC1D13*		-0.061 (0.004)	2.1e-60	4.5e-58
**#12**	10q11.21	rs12779637*	***ANUBL1***, *FAM21C*	*FAM21D* ^4^	0.051 (0.006)	6.6e-19	4e-17
**#14**	12q24.31	rs12822898*, **rs2066938**	*RNF10*, *MLEC*, *UNC119B*, *CAMKK2*	***COQ5*** ^4^	0.049 (0.006)	3.8e-16	1.9e-10
**#15**	15q22.2	rs7162825*, rs12442826, rs7183733, **rs12440281**	***LACTB***, *TPM1*, *APH1B*		0.12 (0.004)	8e-153	1.2e-149
**#16**	21q22.13	rs1571700*	***HLCS***		0.033 (0.003)	2.8e-23	2.3e-21

^1^SNP with strongest metabolite association is presented in **bold** while SNP with strongest eQTL was marked with an asterisk*

^2^Gene with strongest association is presented in **bold**

^3^A q-value<5% was considered as significant, i.e. FDR is controlled at 5%.

^4^These genes are located on the same chromosome as the lead-SNPs at distances larger than 1Mb

### Integrative analysis of mQTLs, eQTLs and expression-metabolite associations

We next aimed to assess whether changes in expression of identified eQTL genes can explain observed SNP-metabolite associations in our study. Therefore, we analyzed the relationship between expression levels of these genes and metabolites. We found 40 study-wide significant associations between gene expressions and metabolites, corresponding to 9 loci and 18 eQTL transcripts (16 unique genes, see Table 3 and S6 Table).

**Table 3:** Results of associations between gene-expressions and metabolites.

Locus	Cytogen. pos.	Regulated Genes^1^	Metabolites^2^	Beta (SE)	p-value	q-value^3^
**#1**	1p31.1	***ACADM***	**C10**, C8	-0.084 (0.019)	1e-05	1.9e-03
**#2**	1q44	***SMYD3***, *JAM3*	**Leu|Ile**, C18, C16	0.2 (0.045)	6.6e-06	1.5e-03
**#3**	2p13.1	***STAMBP***, *NAT8B*	**Leu|Ile**, Q14:Arg/Orn, Q27:Sarc/Gly	-0.24 (0.056)	1.4e-05	2.3e-03
**#6**	4q32.1	***ETFDH***, *TMEM144*	**Q11:Ala/C2**, C2, C10, C16:1, C4, AC-total, C18:1, Leu|Ile, Q1:(Val+Leu|Ile)/(Phe+Tyr), Q20:C5/(Leu|Ile), C8, Q33:C0/(AC-total), C16, Q3:(C16+C18:1)/C2, Q26:Glu/Orn	-0.43 (0.057)	8.2e-14	2.6e-10
**#7**	5q31.1	***SLC22A4***	**Q19:(Leu|Ile)/C3**	-0.27 (0.078)	5e-04	4.1e-02
**#8**	6q21	***AMD1***, *SLC22A16*	**MMA**, C14	0.1 (0.028)	2.8e-04	3.0e-02
**#9**	6q23.2	***MED23***	**C8**	0.06 (0.017)	3.6e-04	3.3e-02
**#10**	8q24.3	***PPP1R16A***, *LRRC14*, *CYHR1*	**Q34:Asp/C2**, Asp, Q16:Asp/Cit, Q12:Ala/Asp, Q1:(Val+Leu|Ile)/(Phe+Tyr), C2	-0.62 (0.11)	4.3e-08	4.5e-05
**#11**	9q34.11	***PPP2R4***, *CRAT*	**MMA**, Q38:MMA/C3	0.16 (0.031)	1.3e-07	1.0e-04

Table displays significant associations of eQTL genes and metabolites for validated loci. Genes and metabolites are ordered according to strength of association. Statistics of strongest associations are also presented.

^1^Gene with strongest association is presented in **bold**

^2^Metabolite with strongest association is presented in **bold**

^3^A q-value<5% was considered as significant, i.e. FDR is controlled at 5%.

**Fig 4:**
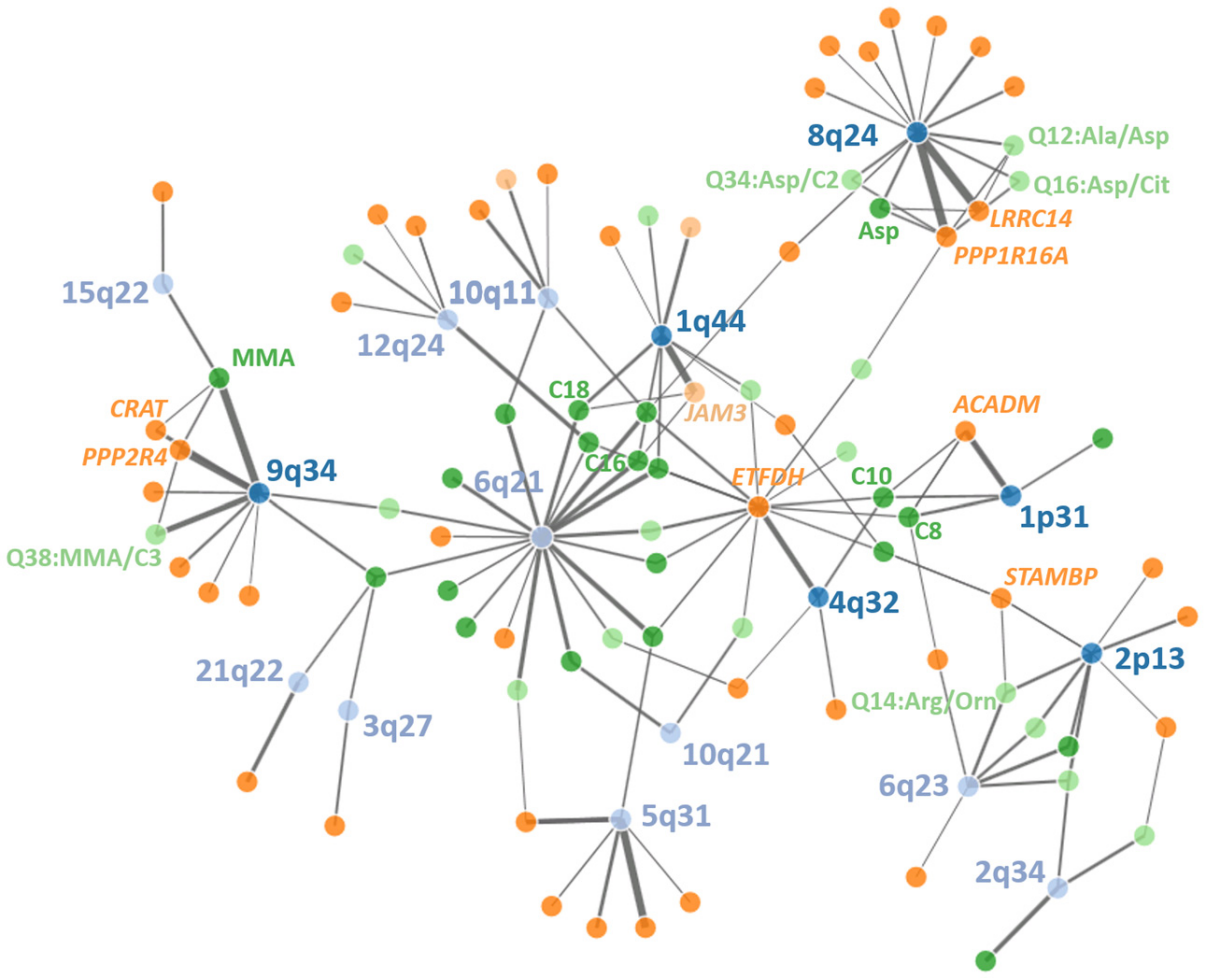
Network of discovered loci, eQTLs and metabolites. Significant relationships between genetic loci (top SNPs), gene-expression in PBMCs and metabolite levels in whole blood are displayed. Line thickness corresponds to amount of explained variance (Lightblue = genetic loci without triangles, darkblue = genetic loci with triangles, lightgreen = cis-regulated genes, darkgreen = trans-regulated genes, light orange = raw metabolites, darkorange = metabolite ratios). An interactive html-document document of the network can be found in the supplement material.

We then integrated information from SNP-metabolite (mQTL), SNP-gene expression (eQTL) and expression-metabolite associations to form association triangles. A triangle is defined by a triple of SNP, transcript and metabolite showing pair-wise associations (see methods for details). We constructed a network of all pairs of associations and their strengths (see Fig 4) to illustrate the multiple relationships between associated genetic loci, genes and metabolites. An interactive html-document to explore the network is provided as supplement material (S4 Fig). Certain overlaps with previously reported molecular interactions exist. These known relationships are summarized in S11 Table. We identified 177 relations containing 21 unique primary associations between features analysed in our study. Additionally, we identified 16 unique molecules potentially connecting features analysed in our study. As expected, these molecules include Proinsulin and Ubiquitin.

Association triangles were further used to test whether variances in gene expression are causally related to variances of metabolite levels. We discovered 38 association triangles mapping to six unique loci including the two novel loci #2 and #10 at 1q44 and 8q24.3, respectively (S7 Table). To estimate the number of such triangles identified by chance, we performed a comprehensive permutation analysis including mQTL, eQTL and expression-metabolite association analysis (S8 Fig). From this, the empirical likelihood of the reported six triangles obtained by chance was estimated to be <1x10^-15^. Particularly, in only two of 100 permutations we obtained a single triangle while in 98 of our 100 permutations, no triangles were observed.

Next, we used Mendelian randomization to establish a causal link between gene expression and the metabolite. We identified 15 metabolite-gene pairs included in 36 triangles (S7 Table). Next, we investigated whether identified eQTLs explained a significant part of the SNP-metabolite association which we could demonstrate for a total of five loci (Table 4). Strongest causal effects were found for novel locus #10 at 8q24.3 associated with several Aspartic acid traits (strongest causal effect for ratio Aspartic acid / Acetylcarnitine via cis-regulation of *PPP1R16A*) and locus #11 at 9q34.11 associated with MMA via *PPP2R4*.

**Table 4:** Integrative analysis and association triangles.

Locus	Cytogen. pos.	Lead-SNPs	Regulated gene	Associated Metabolites	Beta of mQTL	Beta of eQTL	Beta of expression-metabolite association	p-value causality gene expression and metabolite^1^	Explained variance mQTL	Explained variance eQTL	Explained variance expr.-metab. association	p-value
**#1**	1p31.1	**rs1303870**, rs17587071	***ACADM***	**C8**, C10	-0.016	0.027	-0.054	8.3x10^-5^	1.8%	2%	0.7%	4e-03
**#2**	1q44	**rs3811444**	***JAM3***	**C16**	0.044	-0.229	-0.049	2.8x10^-3^	1.4%	13%	0.6%	3.7e-02
**#3**	2p13.1	**rs6546838**	***STAMBP***	**Q14:Arg/Orn**	-0.022	-0.015	0.070	8.7x10^-4^	2.6%	0.9%	0.7%	6.9e-03
**#10**	8q24.3	**rs750472**	***PPP1R16A***	**Q34:Asp/C2**, Q16:Asp/Cit, Asp, Q12:Ala/Asp	-0.170	0.111	-0.619	1.0x10^-7^	1.8%	19.6%	1.5%	3.7e-04
**#11**	9q34.11	**rs11789753**, rs11999428, rs3124505, rs12686182, rs10760593, rs17452603, rs7874044	***CRAT***	**MMA**	-0.127	-0.043	0.107	5.2x10^-3^	2.2%	0.4%	0.6%	2.4e-02
		**rs11789753**, rs10760593, rs3124505, rs7874044, rs12686182, rs17452603, rs11999428	***PPP2R4***	**MMA**, Q38:MMA/C3	-0.127	-0.094	0.165	9.4x10^-7^	2.2%	2.4%	1.4%	2.2e-04

Combinations of SNPs, genes and metabolites for which gene-expression explains at least a nominally significant part of the observed SNP-metabolite association. Best combination of SNP, gene and metabolite is presented in bold with corresponding statistics. Causality of expression and metabolites is determined via Mendelian Randomization. Last column shows p-values of testing whether gene-expression explains a part of the observed SNP-metabolite association (see methods section). Triangles with strongest causality per locus are show in **bold.**

^1^analysed adopting Mendelian Randomisation method

### Associations with clinical traits and diseases

Finally, we explored whether SNP-metabolite associations identified in our study overlap with genetic loci for clinically relevant traits published in the National Human Genome Research Institute (NHGRI) GWAS Catalog. At nine of the 16 validated loci, metabolite associated SNPs matched SNPs previously associated with clinical traits or diseases (S9 Table). We observed associations with platelet and red blood cell properties at three loci associated with acylcarnitines in our study (1q44 (C18), 10q11 (C3) and 15q22 (MMA)) [26–28]. Further, we found that several of our variants were associated with clinical chemistry traits, e.g. fibrinogen (2q34) [29], homocysteine (2q34) [30] and traits reflecting lipid metabolism (HDL-cholesterol at 2q34 and 15q22) [31], purine catabolism (uric acid at 10q21) [32], and kidney function (creatinine at 2p13 and 2q34) [33]. At the 2p13 and 2q34 loci, reported associations for creatinine were also linked to chronic kidney disease [34]. In addition, variants at the 2q34 locus for glycine also convey risk for non-small cell lung cancer [35]. Interestingly, recent studies described a key role for glycine in cancer cell proliferation and tumorigenesis [36,37]. Further, metabolite associations at 3q27 (C5OH+HMG), 5q31 (AC-total), 9q34 (MMA) and 15q22 (MMA) overlapped with associations for Parkinson’s Disease [38], Asthma [39], Hypersomnia [40] and orofacial cleft [41], respectively. These co-localizations may implicate a shared genetic basis (pleiotropy) between complex traits and aid in forming new hypothesis regarding molecular pathomechanisms.

## Discussion

Several GWAS for urine, serum and plasma metabolites have been published using different measurement approaches [8,9,12–21,23]. Here, we report the first genome-wide association study for amino acid and acylcarnitine levels in whole blood. We discovered 25 loci of which 14 were replicated in an independent cohort. Additional two loci were strongly supported by mQTL-studies in serum or plasma [8,9,13–15]. Of these 16 loci, six describe novel SNP metabolite associations, comprising four loci associated with various acylcarnitines and two loci associated with amino acids. Our results demonstrate that studying whole blood can provide additional genetic loci not detected in previous mQTL studies for plasma or other body fluids. This might be attributable to differences in metabolite abundance and components of cellular metabolism not present in plasma (or other cell free body fluids) [25].

Further, we used whole genome expression in peripheral mononuclear cells to establish functional links between SNP-metabolite associations and gene-expression. EQTLs were discovered at 14 loci, including all of our six novel loci. Since eQTL analysis *per se* does not allow inferring causal genes, we performed gene expression association analysis between eQTL genes and metabolites associated with the corresponding SNP. This is a major advantage of our study since we can directly infer causal relationships, whereas most other studies can only report indirect evidence from public eQTL data bases. Besides limitations of gene-expression analysis, such as tissue specificity and numerous other ways for genetic variations to influence the function or abundance of proteins, we identified five loci for which a significant part of SNP-metabolite association was explained by blood eQTLs. These represent novel findings to the best of our knowledge and extend the very few examples of known causal chains between SNPs, gene-expression and metabolites [14,42,43].

### Characteristics and functional hypotheses of novel loci

At two of the six newly identified loci (6q23, *ARG1* and 21q22, *HLCS*), rare variants are known to cause autosomal recessive inborn errors of metabolism, providing a strong biological plausibility for the SNP-metabolite associations. Mutations in *ARG1 (6q23)*, encoding arginase, the enzyme which catalyzes the hydrolysis of arginine, are the cause of Argininemia (OMIM #207800). Here, we report common variants of *ARG1* to be associated with arginine levels. Likewise, defects in *HLCS (21q22)* are responsible for holocarboxylase synthetase deficiency (OMIM #253270) with affected individuals displaying elevated levels of C5OH+HMG. In line with this observation, the lead SNP at the *HLCS* locus exhibited a strong cis-eQTL and the allele responsible for higher *HLCS* expression was associated with lower C5OH+HMG levels.

A third novel locus (#8; 6q21) associated with multiple acylcarnitines (lead phenotype: AC-total) also contained a gene with direct biochemical relationship to the associated metabolites, namely *SLC22A16*, encoding an organic cation/ carnitine transporter. Gene expression of *SLC22A16* was regulated in *cis* at this locus, but *SLC22A16* gene expression was not correlated with acyl-carnitine concentrations in whole blood. In fact, the strongest SNP metabolite association at this locus was observed for a non-synonymous coding SNP (rs12210538) in *SLC22A16*, which is predicted to be damaging by Polyphen and SIFT [44,45]. These findings suggest that associations at 6q21 are more likely driven by this non-synonymous coding mutation than by gene expression of *SLC22A16*.

The remaining three novel loci relate to candidate genes with no prior connection to metabolism to the best of our knowledge. For the locus at 10q11.21, associated with C2 and C3, we observed cis-effects on *ANUBL1* and *FAM21C* expression, but gene expressions of both transcripts were not correlated with either C2 or C3. Thus, additional work will be required to explore the causal link between genetic variation at the 10q11.21 locus and C2 and C3 blood concentrations.

At novel locus 8q24.3, integration of SNP, eQTL and gene-expression data let to the identification of *PPP1R16* as putative causal gene for the association with aspartic acid and corresponding ratios (lead phenotype: alanine / aspartic acid). While we detected strong cis-effects on expression of two local genes, *PPP1R16A* and *LRRC14*, only the eQTL of *PPP1R16A* partly explained the observed SNP-phenotype associations. Future studies need to address how *PPP1R16A*, a gene involved in signal transduction [46], may be affecting blood levels of aspartic acid.

Finally, we identified *JAM3* encoding the junctional adhesion molecule C (JAM-C) as a novel candidate gene of acylcarnitine metabolism. Top associated SNP rs3811444 (1q44) exhibited an exceptionally strong trans-eQTL for *JAM3*, located at 11q25. This trans effect was also described by other eQTL studies [47]. Gene expression of *JAM3* correlated with several long chain acyl-carnitines (i.e. C16) and explained a significant part of the SNP-metabolite association. JAM-C participates in cell-cell adhesion, leukocyte transmigration and platelet activation. The soluble form of JAM-C has been shown to mediate angiogenesis [48]. Homozygous mutations in *JAM3* cause hemorrhagic destruction of the brain, subependymal calcification, and congenital cataracts (HDBSCC, OMIM #613730). At present, the potential functional role of *JAM3* in acyl-carnitine metabolism remains elusive.

### Novel evidence at known metabolite loci

In addition to the identification of novel loci, we replicated and extended functional evidence for SNP-metabolite associations at ten loci previously described in GWAS for serum or plasma metabolites (Table 1). The majority of these loci contain highly plausible candidate genes based on their biologic function in metabolism (*MCCC1*, *ETFDH*, *SLC22A4/5*, *ACADM*, *ACADS*, *CPS1*, *CRAT*). Rare loss of function mutations in these genes cause Mendelian inborn errors of metabolism and measuring the respective marker metabolites in whole blood spots is part of neonatal screening programs throughout the world [1]. Here, we validated common variants located in non-coding DNA with modest effect sizes on blood metabolites. Additionally, we found blood eQTLs for *MCCC1*, *ETFDH*, *SLC22A4/5*, *ACADM*, and *CRAT*. This is in line with evidence from other complex genetic traits, demonstrating that most associations for common variants arise in non-coding DNA and emphasizes the importance of regulatory variants in modulating gene expression [49,50]. A striking example is the ACADM locus, where SNPs have been associated with C8 and C10 levels [13,14,20,21]. In our study, gene-expression of *ACADM* was associated with C8 and C10 blood levels and we showed for the first time that this relationship was causal explaining a part of the observed SNP association.

In conclusion, our study expanded the current knowledge on the genetic regulation of human blood metabolites by adding six novel genetic loci. Furthermore, by integrative analysis of SNP, gene expression and metabolite data, we derived mechanistic insights into the molecular regulation of blood metabolites. At several loci, we provide evidence for metabolite regulation via gene-expression and observed overlaps with GWAS loci for other complex traits and diseases, pointing towards potential pathomechanisms via metabolic alterations. Additional functional studies are required to elucidate the cellular mechanisms how the discovered candidate genes affect metabolic pathways and relate to disease pathology.

## Materials and Methods

### Cohorts

LIFE Leipzig Heart is an observational study in a Central European population designed to analyze genetic and non-genetic risk factors of atherosclerosis and related vascular and metabolic phenotypes [51]. Patients undergoing first-time diagnostic coronary angiography due to suspected stable CAD with previously untreated coronary arteries, patients with stable left main coronary artery disease and patients with acute myocardial infarction were recruited. The latter were excluded for the present analysis.

The study meets the ethical standards of the Declaration of Helsinki. It has been approved by the Ethics Committee of the Medical Faculty of the University of Leipzig, Germany (Reg. No 276–2005) and is registered at ClinicalTrials.gov (NCT00497887). Written informed consent including agreement with genetic analyses was obtained from all participants. In this analysis, we considered a total of 2,464 individuals. From these, 2,107 had complete genotype, metabolite and covariate data qualifying them for GWAS analysis (descriptive statistics can be found in S9 Table). A subset of 1,856 individuals had complete data of genotypes, gene expression, metabolites and covariates. These individuals were used for integrative analyses (see study design, S1 Fig).

The Sorbs were recruited from the self-contained Sorbs population in Germany [52–54]. All individuals were at fasting state. Phenotyping included standardized questionnaires for past medical history and family history, collection of anthropometric data (weight, height, waist-to-hip ratio) and results from an oral glucose tolerance test. A complete set of high-quality genotype data, metabolites and covariates was available for 923 subjects (S9 Table). The study was approved by the ethics committee of the University of Leipzig and all subjects gave written informed consent before taking part in the study.

### Study design

An overview of the study design is presented in S1 Fig. In brief, we first performed a genome-wide metabolite quantitative trait (mQTL) analysis in the LIFE Leipzig Heart cohort, with replication of the top-SNPs in the Sorbs cohort. Following this two-stage design, we applied a liberal cut-off of 1.0x10^-7^ for the initial GWAS to identify candidate loci. A stringent cut-off is applied at the replication stage where we control the (study-wide) FDR at 5% based on permutation analysis [55]. This accounts for the correlation structure of individuals, SNPs and metabolites and the multiple testing issue (for details see below section “Genome-wide association analysis and SNP replication”).

Functional relevance of identified loci was studied in the LIFE Leipzig Heart cohort by analyzing expression quantitative traits (eQTL) and gene expression-metabolite associations followed by causal inference regarding discovered associations.

### Metabolomic analysis and data processing

Venous blood samples were obtained from all study participants and 40μl of native EDTA whole blood were spotted on filter paper WS 903 (Schleicher and Schüll, Germany) in the LIFE Leipzig Heart study. In the Sorb cohort, 40μl cell suspension obtained after plasma centrifugation (10 min at 3500 x g) were spotted on filter paper. All blood spots were stored at -80°C after 3 hours of drying until mass spectrometric analysis. Sample pretreatment and measurement is described elsewhere [56–58]. In brief, 3.0 mm diameter dried blood spot punches (containing 3 μL whole blood) were extracted with methanol containing isotope labelled standards. After sample extraction and derivatization, analysis was performed on an API 2000 tandem mass spectrometer (Applied Biosystems, Germany). Quantification of 26 amino acids, free carnitine and 34 acylcarnitines including related metabolites was performed using ChemoView 1.4.2 software (Applied Biosystems, Germany). Samples were analysed within 23 analytical batches with two quality controls samples in each batch. Mean inter-assay coefficients of variation were below 11% for amino acids and below 19% for acylcarnitines. Further, using these 61 directly measured analytes, we derived a number of biologically relevant sums (n = 1, total acylcarnitine) and ratios (n = 34) to assess reaction equilibria within physiological pathways and processes (e.g. Fischer’s ratio [59]). Consequently, a total of 96 quantities were analyzed as GWAS traits. A list of metabolites and quantities is presented in S1 Table.

Metabolites with more than 20 percent of values below detection limit were dichotomized for analysis (below detection limit versus above detection limit). This applies for the metabolites C5:1, C6DC, C14OH, C16OH, MeGlut, C18:1OH, C18:2OH, C18OH and C20:3. Quantities were arsinh-transformed (area sinus hyperbolicus) which is close to a log-transformation for large values but does not emphasize differences between small values and can operate on values of zero. Transformed quantities were approximately normal distributed. Values outside of the Interval Mean ± 5*SD were considered as outliers and were removed to stabilize subsequent regression analysis.

We previously analysed a variety of factors influencing blood metabolites. Age, sex, diabetes and fasting status show pronounced effects on several metabolites while log-BMI, smoking and some blood traits showed effects on selected metabolites. Therefore, we decided to adjust our analyses for these potential confounders.

### SNP genotyping and quality control

#### LIFE leipzig heart samples

DNA was extracted from peripheral blood using the Invisorb Spin Blood Maxi Kit (Stratec) as described elsewhere [60]. Samples where genotyped using an Affymetrix Axiom SNP array with custom option comprising a total of 624,908 SNPs. The Axiom CEU array served as a backbone of our custom array. In addition 62,471 autosomal SNPs were placed on the array corresponding to 44 genomic regions previously associated with cardiovascular disease and metabolic risk factors, in particular plasma lipids. Genotyping was performed at Affymetrix (Santa Clara, Ca; USA). 2,925 out of 3,036 DNA samples were successfully genotyped and were called in combination by Affymetrix Power Tools version 1.12. As further sample-wise quality control, we filtered individuals with sex mismatch, call rate<97%, low or high mean squared difference of individual’s genotype and expected genotype according to box plot outlier criteria, duplicates, implausible relatedness according to Wang et al. [61] and outliers of principal components analysis (6SD criterion of EIGENSTRAT [62]). Thereafter, a total of N = 2,838 individuals remained for analysis. After the final step of sample quality control, the population genetic structure was homogenous (see S5 Fig). Based on this sample, we determined our SNP quality filter as follows: non-autosomal SNPs, minimal plate-wise call rate <90%, i.e. the minimum of the SNP call rate over all plates (our criteria implies that the conventional overall SNP call rate is greater than 94.3% and its 10^th^ percentile is greater than 99.2%), p-value of asymptotic Hardy-Weinberg equilibrium test <1.0x10^-6^, p-value of the association of SNP allele frequency with plate number <1.0x10^-7^. A total of 566,359 SNPs passed all criteria.

Genotype imputation was performed using IMPUTE v2.1.2 (http://mathgen.stats.ox.ac.uk/impute/impute_v2.html). HapMap2 CEU, Release 24, dbSNP-build 126, NCBI built 36 served as reference panel comprising a total of 3,974,237 autosomal SNPs. 555,911 of our measured SNPs were successfully mapped to the reference. As post-imputation quality control we discarded all SNPs with minor allele frequency ≤1% or with IMPUTE-info score ≤ 0.3. According to these criteria, a total of 2,619,023 SNPs were analysed.

#### Sorbs samples

Subjects were either genotyped using the 500K Affymetrix GeneChip or Affymetrix Genome-Wide Human SNP Array 6.0. For genotype calling, BRLMM algorithm (Affymetrix, Inc) was applied for 500K and Birdseed algorithm for Genome-Wide Human SNP Array 6.0. Details of genotyping have been described elsewhere [53]. Quality control of samples was performed as described in Gross et al [52] resulting in N = 977 individuals with genotypes of good quality (N = 483 genotyped with 500K, N = 494 genotyped with 6.0). Three ethnic outliers were identified by a ‘drop one in’ procedure to avoid bias by the relatedness structure within the Sorbs (see [63] for details). These samples were excluded from subsequent analyses. After removing these samples, principal components revealed a homogenous population structure (see S6 Fig). To account for relatedness, a drop-one-in procedure was used for principal components analysis (see [63] for details).

Genotype imputation was performed without prior SNP filtering and separately for individuals genotyped with 500K and 6.0 respectively as described [63]. The same software and reference panel was used as for the LIFE Leipzig Heart samples.

### Genome-wide association analysis and SNP replication

Genome-wide association analyses for blood 96 metabolites was performed in the LIFE Leipzig Heart samples (N = 2,107 with complete phenotypes, covariates and high-quality genotypes). Associations were tested by linear regression models using gene-doses of imputed SNPs. We adjusted for age, sex, log-BMI, diabetes status, smoking status, fasting status, haematocrit, platelet count, white blood cell count and the first three genetic principal components. Results revealed no signs of genomic inflation (maximum lambda equal 1.018, see S10 Table). To avoid reporting of redundant SNP information, the top-SNP list was ordered according to minimal p-values and pruned applying a linkage disequilibrium cut-off of r^2^<0.3.

Replication analysis was performed in the independent cohort of Sorbs (N = 923 with complete genotype and metabolite data) and for all combinations of SNPs and metabolites achieving a p-value of <10^−7^ in our first stage GWAS. Based on our unpruned GWAS top-list, we retrieved all SNPs within a ±50kB environment which were successfully imputed in the Sorbs (IMPUTE-info score>0.3 in both, 500K and 6.0 subsample). Then, on the basis of the LIFE Leipzig Heart data, we assessed which of these SNPs are the best proxies of the corresponding top-SNPs to pair GWAS top-SNPs with optimal proxies of good quality within the Sorbs study.

Associations between pairs of proxies and metabolites were again analyzed using linear regression analyses of gene-doses. Here, we adjusted for age, sex, log-BMI, diabetes status, smoking status, haematocrit, platelet count, white blood cell count and the relatedness structure ([52,64,65], function “polygenic” of the “GenABEL” package of R was used to deal with the relatedness structure [63]).

Since test statistics are correlated due to LD between SNPs and correlations between metabolites, we decided to control the false-discovery rate (FDR) at 5% rather than family-wise error rates. Null-distribution for q-value calculation was determined by permutation analysis. For this purpose, 1000 random permutations of the links between SNPs and metabolites were analyzed.

### Novelty assessment of SNP-metabolite associations and search for pleiotropic effects

We compared our results with published GWAS hits on the basis of the GWAS catalogue (http://www.genome.gov/gwastudies/, date of download March, 4^th^, 2014). Required LD information was derived from HapMap3 (release 28) and 1000genomes project (release 20110521 version 3 f, restricted to SNPs with a MAF ≥ 1%). In addition, further evidence from published mQTL studies was manually included in this analysis to assess novelty of our results. A total of 13 studies were analyzed [8,9,12–21,23] (see also S4 Table). A locus was considered as novel if none of its SNPs were in linkage disequilibrium (r^2^>0.3) with any published mQTL hit reaching study-wide significance as defined by the authors of the corresponding publication. To increase relevance, we did not match the associated metabolic phenotypes between our study and the published ones, i.e. our approach of considering loci as novel is conservative.

In complete analogy to this analysis, we determined whether our top hits are associated with other traits for which results are published in the GWAS catalogue as well as those reported in two GWAS on plasma lipids [10,31]. These traits could point toward other causal or pleiotropic effects. If applicable, information on genetic disorders related to our loci were retrieved from OMIM (http://omin.org).

### Gene-expression measurement and pre-processing

Peripheral blood mononuclear cells were isolated in the LIFE Leipzig Heart cohort using Cell Preparation Tubes (CPT, Becton Dickinson) as previously described [66]. Total RNA was extracted using TRIzol reagent (Invitrogen) and quantified with an UV-Vis spectrophotometer (NanoDrop, Thermo Fisher). 500 ng RNA per sample were ethanol precipitated with GlycoBlue (Invitrogen) as carrier and dissolved at a concentration of 50–300 ng/μl prior to probe synthesis. N = 2,501 samples were hybridised to Illumina HT-12 v4 Expression BeadChips (Illumina, San Diego, CA, USA) in batches of 48 and scanned on the Illumina HiScan instrument according to the manufacturer’s specifications [60]. Documentation of sample processing included batch information at any processing step allowing adjustment in subsequent data analysis.

Raw data of all 47,323 probes was extracted by Illumina GenomeStudio, 47,308 probes could be successfully imputed in all samples. Data was further processed within R/ Bioconductor R [67]. Individuals having an extreme number of expressed genes (defined as median ± 3 interquartile ranges (IQR) of the cohort’s values) were excluded. Transcripts that were not expressed according to Illumina’s internal cut-off as implemented in the “lumi” Bioconductor package (p ≤ 0.05 in at least 5% of all samples) were excluded from further analysis. Expression values were quantile-normalised and log2-transformed [68]. For further outlier detection, we calculated the Euclidian distance between all individuals and an artificial individual which was defined as the average of samples after removing 10% samples farthest away from the average of all samples. Individuals with a distance larger than median + 3 IQR were excluded. Furthermore, we defined for each individual a combined quantitative measure combining quality control features available for HT-12 v4 (i.e. ratio of levels of perfect-match vs. mismatch control probes, mean signal of perfect-match control probes, mean of negative control probes and labelling-control probes, ratios of high-concentrated, medium-concentrated and low-concentrated control-probes, mean of house-keeping genes, Euclidian distances of expression values, number of expressed genes, mean signal strength of biotin-control-probes). We calculated Mahalanobis-distance between all individuals and an artificial individual having average values for these quality control features. Individuals with a distance larger than median + 3 IQR were excluded. Transcript levels were adjusted for known batch effects using an empirical Bayes method as described [69] and residualised for age, sex, monocyte counts and lymphocyte counts. Additionally, we calculated principal components of the expression data and residualised for the first five principal components of expression data to account for unmeasured batch effects [70]. Pre-processing resulted in 28,295 expression probes corresponding to 19,519 genes. Chromosomal mapping of expression probes and assignment of gene names was done using information as reported by the manufacturer (HumanHT-12_V4_0_R2_15002873_B).

### eQTL and gene-expression association analysis

After quality control, combined SNP and gene-expression data were available for a total of 2,112 individuals, from which 1,856 had been included in the GWAS. eQTL analysis of the pruned GWAS top-list was performed by linear regression analysis of gene-doses using the R add-on package Matrix eQTL [71]. EQTLs were considered as cis-regulated if the distance between SNP and the centre of the associated expression probe was not larger than 1 Mb, otherwise they were considered as trans-regulated. Cis- and trans- specific significance thresholds were derived by a Benjamini-Hochberg (B-H) procedure implemented in Matrix eQTL. For our data, cis associations with a p-value up to 0.0039 and trans-associations with a p-value up to 3.6x10^-14^ were considered study-wide significant at FDR<5%. B-H q-values were empirically confirmed by 100 permutation tests (permutation of SNP and gene-expression profiles). Further details can be found elsewhere [72].

Association analysis of gene-expression and metabolites was performed in 1,957 individuals for which both information as well as covariates were available (1,856 of these individuals had been included in the GWAS). Again, we adjusted for age, sex, log-BMI, diabetes status, smoking status, fasting status, haematocrit, platelet count, white blood cell count. FDR was controlled at 5%.

As we observed multiple relationships between genetic loci, gene-expressions, and metabolites, we visualized all associations found at FDR 5% in a network. Previously published relations were identified by mapping genetic loci, genes, and metabolites from mQTL, eQTL, and gene-expression-metabolite association analysis to QIAGEN’s Ingenuity Pathway Analysis (IPA, QIAGEN Redwood City, www.qiagen.com/ingenuity), as of May, 2015). This database includes, among many other information, data on genome-wide protein-protein interactions, activation / co-localization and enzymatic reactions. Significantly associated SNPs were represented by the three most proximal genes and metabolite ratios by the individual nominator and denominator.

### Identification of association triangles

For a more detailed characterization of the observed SNP-metabolite associations, we integrated genotype, gene expression and metabolite data to construct association triangles. A triangle is defined as a SNP that is significantly associated with both, a certain expression probe and a certain metabolite. Thereby, the expression probe must be also associated with the metabolite. For this purpose, we first determined the top associated SNP per locus, its corresponding best associated metabolite and eQTLs of that SNP (FDR = 5%, see above). Resulting triples of SNP, transcript level of eQTL and metabolite level were restricted to those showing a significant association between mRNA expression and metabolite level (FDR = 5%, see above). These gene-expressions were considered as possible explanatory quantities of the SNP-metabolite association.

We simulated the expected number of these association triangles under the null distribution by performing a comprehensive permutation analysis: We performed 100 permutations where we randomly assigned expression datasets and metabolic datasets to genetic datasets. We analysed these datasets for mQTLs, eQTLs, and gene-expression associations in accordance to our original analysis. For each of these 100 permutation-based datasets, we counted the number of pairwise associations and association triangles and compared it with the results of our original dataset. We calculated the empirical likelihood of triangles by comparing the observed number of six triangles with the number of triangles under the null assuming a Poisson distribution.

In order to exclude spurious correlation between gene-expression and metabolites as a cause of the observed association, we performed a Mendelian randomization analysis using our eQTL SNPs as instrumental variables [73]. In general, it is not easy to prove that the conditions of Mendelian randomization are fulfilled. In particular, a direct SNP effect on metabolites cannot be excluded, violating one of the assumptions [74]. Therefore, we adapted the Mendelian randomization analysis by using the residuals of metabolites regarding the remaining direct SNP effects (see also S1 Text for an extended discussion). Standard errors of Mendelian randomization effects were derived by Jackknife [75].

Furthermore, we tested whether gene-expressions explain at least parts of the observed mQTL associations. A subset of 1,856 individuals for which SNP, gene-expression, metabolite and covariate data were available, was eligible for this purpose. We analysed regression models of metabolites in dependence on SNPs, covariables and with or without gene-expression. We asked whether the absolute value of the beta-estimator of the SNP is reduced if gene-expression is added to the model. In this case, gene-expression explains a part of the observed SNP-metabolite association. The difference of these SNP beta-estimators is tested against zero by calculating Jackknife standard errors. This analysis also provides evidence for causal relations between genetic variants, gene-expression levels and metabolite concentrations. Since we observed that it is more stringent and conservative than Mendelian randomization analysis, our conclusions regarding causality are based on this type of analysis.

To gain additional insights into possible functional mechanisms of our loci, we performed the same analysis for all independently associated top-SNPs.

## Supporting Information

S1 Fig Study design. Analysis steps, required data and number of loci with significant results at each stage are shown. For causal inference, association triangles were analysed. A triangle is defined as a SNP that is significantly associated with both, a certain expression probe and a certain metabolite. Thereby, the expression probe must be also associated with the metabolite.
(PDF)

S2 Fig QQ-plots of GWAS. Quantile-quantile plots of (–log10)-p-values for our genome-wide association study of amino acids and acylcarnitines in the LIFE Leipzig Heart study. Post-analysis quality control was applied prior to plotting the results.
(PDF)

S3 Fig Regional association plots of GWAS hits. Regional association plots of all loci achieving p-values <1.0x10^-7^ for any of the metabolites and metabolite ratios. Association results are only shown for corresponding lead metabolites.
(PDF)

S4 Fig Interactive visualization of association structure between genetic loci, genes and metabolites (HTML). Significant relationships between genetic loci (top SNPs), gene-expression in PBMCs and metabolite levels in whole blood are displayed in an interactive way allowing the user to explore the network. Line thickness corresponds to amount of explained variance (Lightblue = genetic loci without triangles, darkblue = genetic loci with triangles, lightgreen = cis-regulated genes, darkgreen = trans-regulated genes, light orange = raw metabolites, darkorange = metabolite ratios).
(HTML)

S5 Fig Genetic Principal Components Analysis of LIFE-Heart samples (initial GWAS samples). We present the first ten Principal Components for 2,107 LIFE-Heart samples included in our initial GWAS.
(PDF)

S6 Fig Genetic Principal Components Analysis of Sorbs samples (replication samples). We present the first ten Principal Components of our replication sample of Sorbs individuals (red) in comparison to HapMap CEU (black).
(PDF)

S7 Fig Interactive eQTL map of mQTL loci. We present an interactive html-version of Fig 3. Each point represents an eQTL. Test statistics of each eQTL are available as tooltip. For clarity, on chromosome 15 only the strongest cis-eQTL is shown.
(HTML)

S8 Fig Empirical distribution of association triangles. We performed 100 permutations including mQTL (sub-figure A), eQTL (sub-figure B), and gene-expression association analysis (sub-figure C) using the same cut-offs as in our original analysis. The aim was to simulate a null-distribution of association triangles (sub-figure D). For all analyses, we observed significantly more associations than expected by chance. Particularly, no association triangles were found in 98 permutations while only one triangle was found in two permutations. In our original analysis, we observed six triangles.
(PDF)

S1 Table Metabolites and definition of metabolite ratios. Overview of analyzed metabolites and ratios, as well as the associated metabolic pathways are displayed.
(XLSX)

S2 Table Results of mQTL GWAS in LIFE Leipzig Heart (discovery cohort). Detailed results of the discovery cohort.
(XLSX)

S3 Table Replication of mQTL GWAS hits in the independent cohort of Sorbs. Detailed results of the replication cohort. All pairs of variants and metabolites showing association with p-value ≤ 1x10^-7^ in the initial GWAS were included in the replication.
(XLSX)

S4 Table Comparison of mQTL GWAS hits with published genetic association studies of metabolites. For all lead-SNPs of validated loci, we analyzed whether the same or SNPs in linkage disequilibrium (*R* ^*2*^ ≥0.3) were associated in previously published genome-wide association studies on metabolites. *R* ^*2*^: linkage disequilibrium between lead-SNPs of our study and the previously published SNPs.
(XLSX)

S5 Table Results of eQTL analysis of mQTL hits in LIFE Leipzig Heart. For all lead-SNPs of validated loci, we show eQTLs in PBMCs. Results are reported for a cis- and trans-specific FDR of 5%, respectively.
(XLSX)

S6 Table Results of association analysis of gene-expressions and metabolites in LIFE Leipzig Heart. For all associated gene expression probes identified in eQTL analysis, we analyzed association with metabolites and metabolite ratios.
(XLSX)

S7 Table Integrative analysis of mQTLs, eQTLs and expression-metabolite associations in LIFE Leipzig Heart. Shown are all identified association triangles. A triangle is defined as a SNP that is significantly associated with both, a certain expression probe and a certain metabolite. Thereby, the expression probe must be also associated with the metabolite. *R* ^*2*^: explained variance.
(XLSX)

S8 Table Comparison of mQTL GWAS hits with GWAS catalogue. For all lead-SNPs of validated loci, we analyzed whether the same or linked SNPs (*R* ^*2*^≥0.3) were associated in previously published genome-wide association studies as reported in the GWAS catalogue (downloaded at March 4^th^, 2014) or two large GWAS on plasma lipids [10,31]. *R* ^*2*^: linkage disequilibrium between lead-SNP of our study and the previously published SNP.
(XLSX)

S9 Table Cohort descriptions. We present major characteristics of our study populations used for GWAS (LIFE Leipzig Heart) and replication (Sorbs). For quantitative parameters we present median and interquartile range.
(XLSX)

S10 Table Genomic inflation. Overview of observed genomic inflation factors (lambda) for the mQTL GWAS in LIFE Leipzig Heart (discovery cohort). We observed no evidence for population stratification as lambda ranged from 0.977 to 1.018.
(XLSX)

S11 Table Reported relationships between significant hits from the mQTL, eQTL, and gene expression-metabolite association of this study. We used Ingenuity Pathway Analysis in order to identify previously published relationships between significant hits from our study shown in Fig 4 and S4 Fig. Locus: Locus of Molecule 1 and Molecule 2. Molecule 1 / 2: Molecules for which relation is described, SNPs are represented by their three nearest genes. mQTL: Molecule was observed as significant finding in the mQTL GWAS or replication of this study. eQTL: Molecule was observed as significant finding in the eQTL analysis of this study. gene—metab: Molecule was observed as significant finding in the gene-expression—metabolite association analysis of this study. Citation: Source of relation description.Prim./ Second. Relation: Prim. = primary relation, i.e. relation directly linking two molecules both observed as significant findings in this study, Sec. = secondary relation, i.e. Molecule 1 originates from this study and is related with a certain Molecule 2 from Ingenuity pathway-database. The pathway molecule is also related with a further Molecule 1 from this study written in the following on the next row. Hence, a secondary relation between Molecule 1 from the first and Molecule 1 from the second row via the same Molecule 2 from the database exists. Direct/Indirect Interaction: D = Direct Interaction: There is a physical interaction between Molecule 1 and Molecule 2, I = Indirect Interaction: Both molecules are related via a certain mechanism, but no physical interaction is reported yet. Interaction type: Interaction as defined in Ingenuity pathway analysis: A = Activation, CP = Chemical-Protein interaction, E = Expression (includes metabolism/ synthesis for chemicals), EC = Enzyme Catalysis, I = Inhibition, L = Molecular Cleavage (includes degradation for Chemicals), LO = Localization, M = Biochemical Modification MB = Group/complex Membership, P = Phosophorylation / Dephosphorylation, PP = Proten-Protein binding, RB = Regulation of Binding, RE = Reaction, T = Transcription, UB = Ubiquination; Relationship: Short description of reported relationship between Molecule 1 and Molecule 2.
(XLSX)

S1 Text Extended methods and discussion of Mendelian randomization analysis. (PDF)
