# Supplementary figures and images for: Integration of Genome-Wide SNP Data and Gene-Expression Profiles Reveals Six Novel Loci and Regulatory Mechanisms for Amino Acids and Acylcarnitines in Whole Blood
Source: PLoS Genet. 2015 Sep 24;11(9):e1005510. doi: 10.1371/journal.pgen.1005510 (PMC4581711; doi:10.1371/journal.pgen.1005510)

Figure S1 (study design)

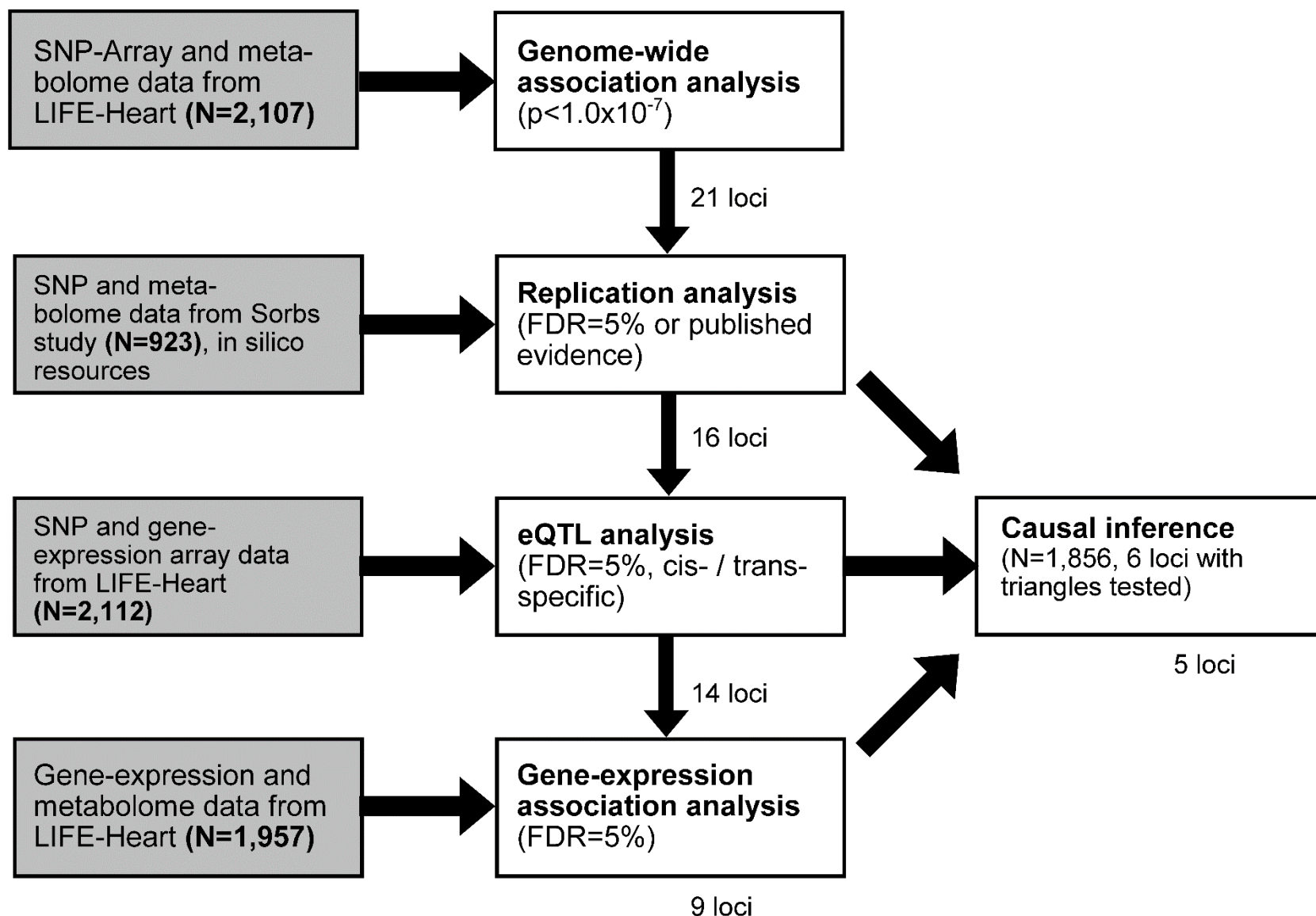

Supplement: S1 Fig — Analysis steps, required data and number of loci with significant results at each stage are shown. For causal inference, association triangles were analysed. A triangle is defined as a SNP that is significantly associated with both, a certain expression probe and a certain metabolite. Thereby, the expression probe must be also associated with the metabolite. (PDF) [file pgen.1005510.s001.pdf]

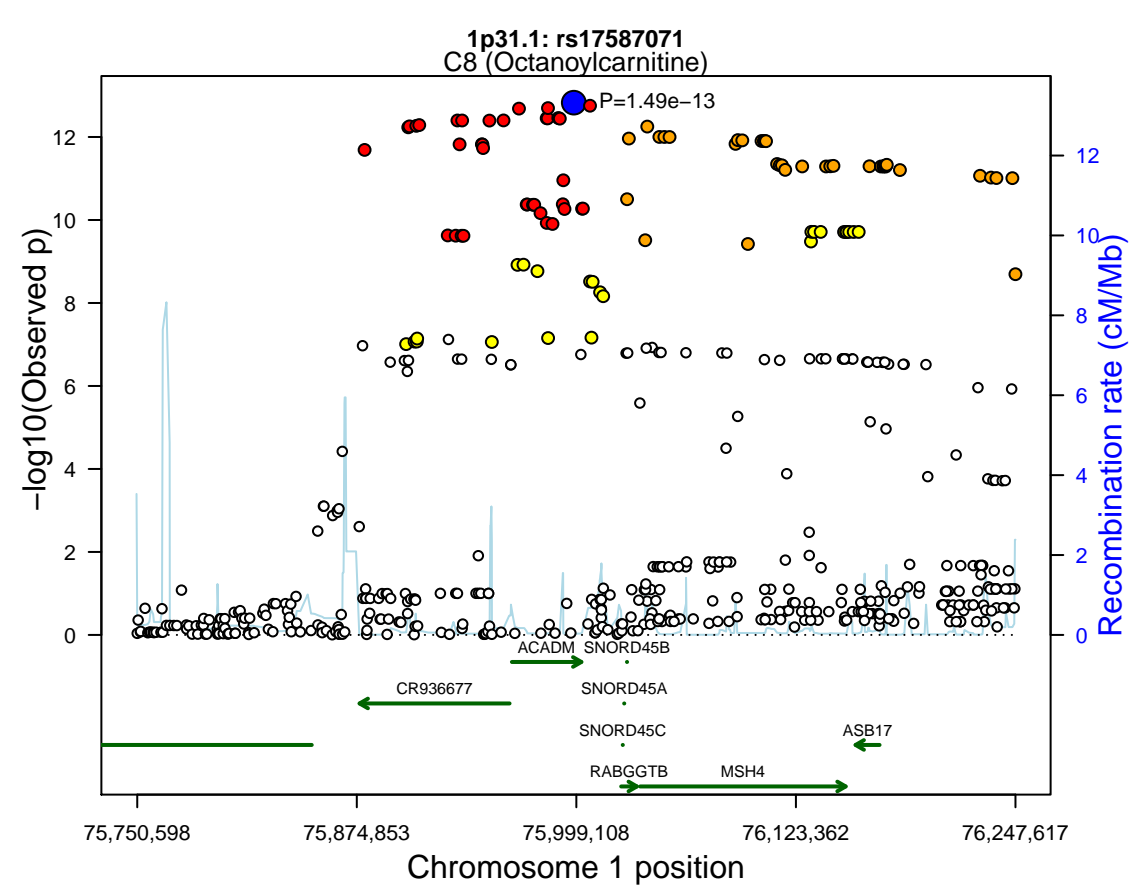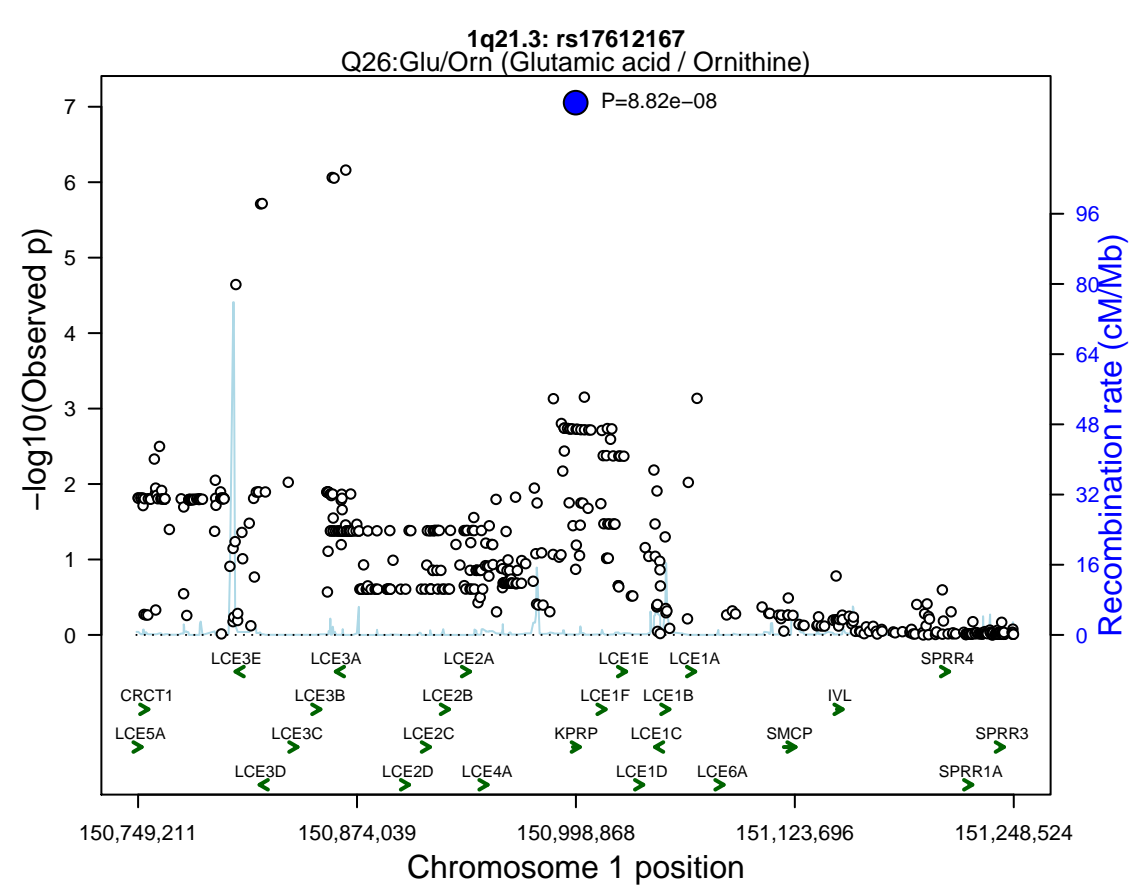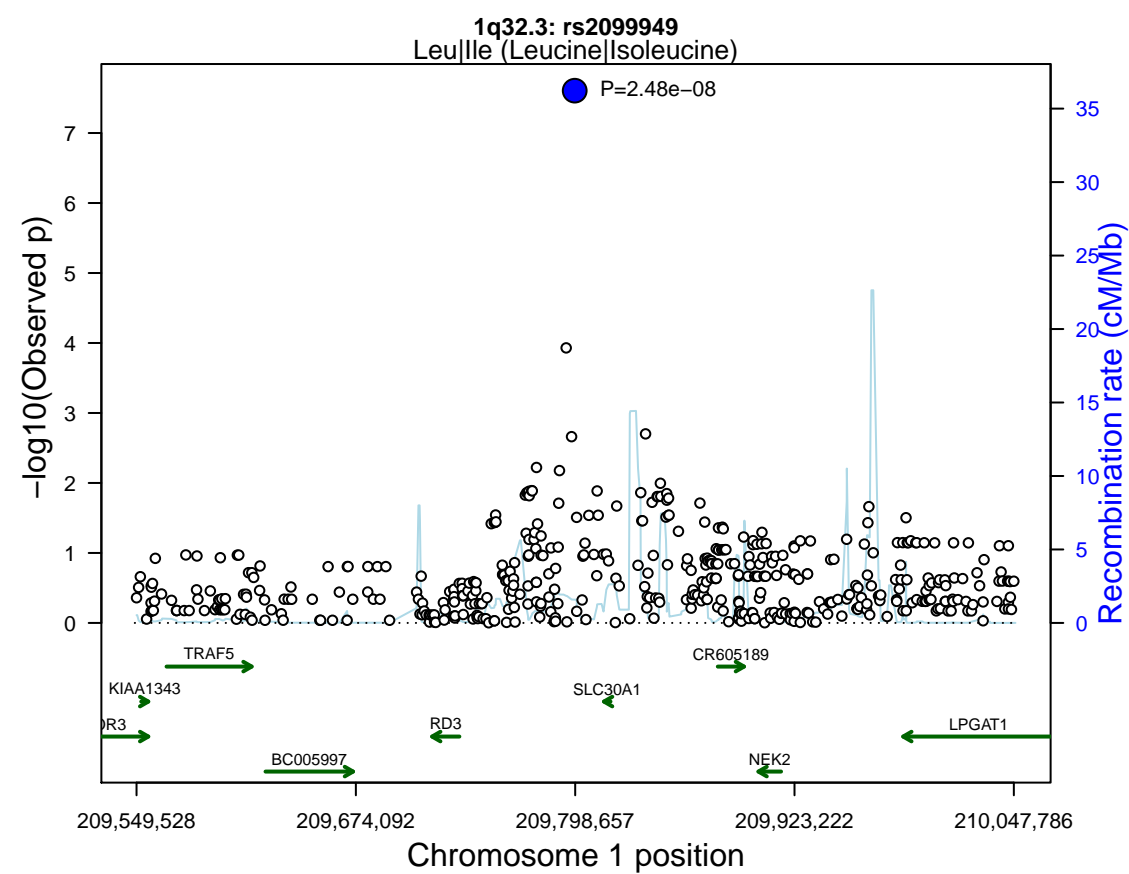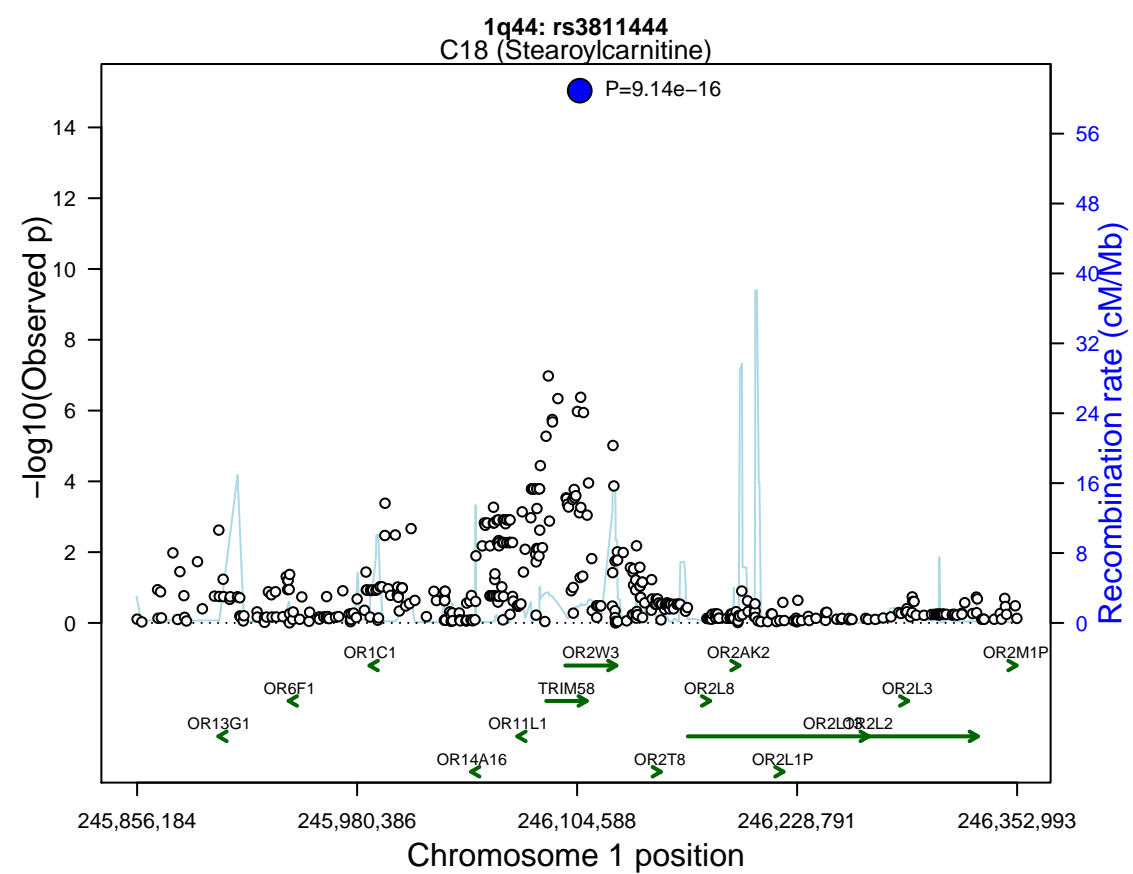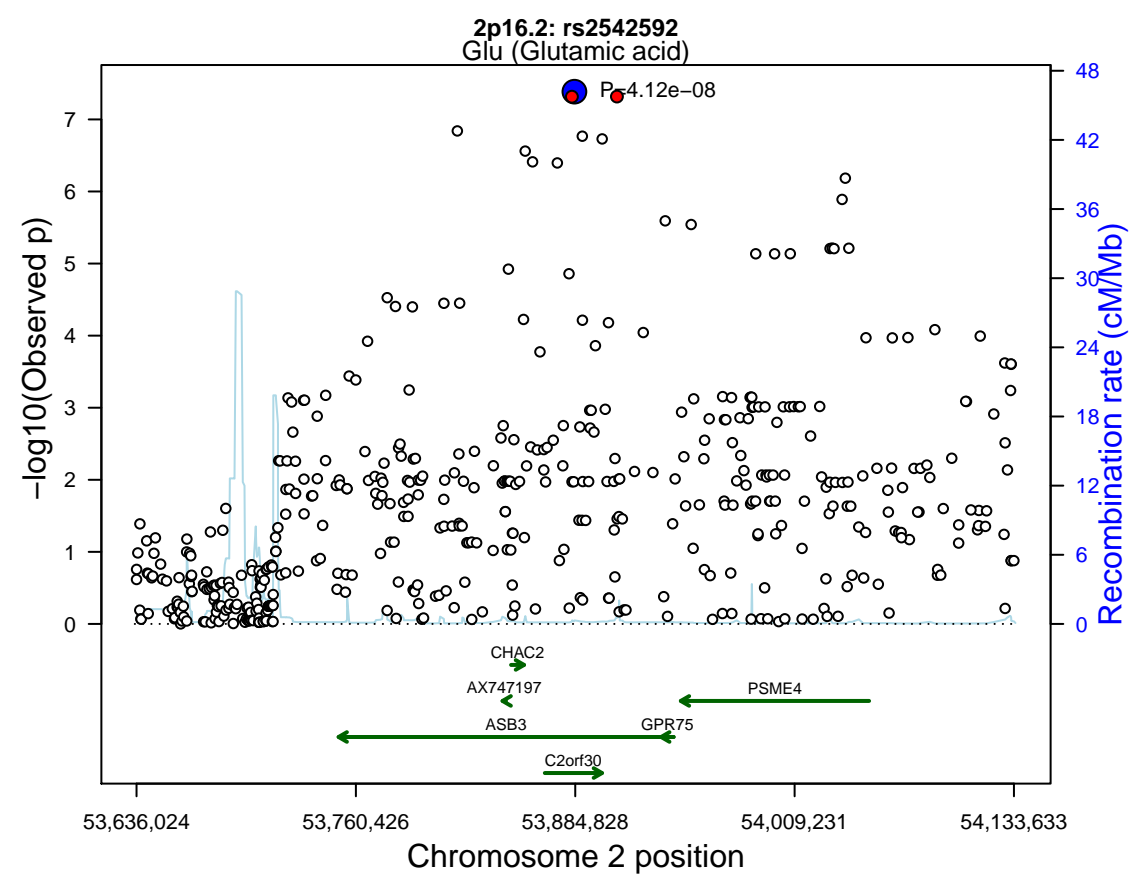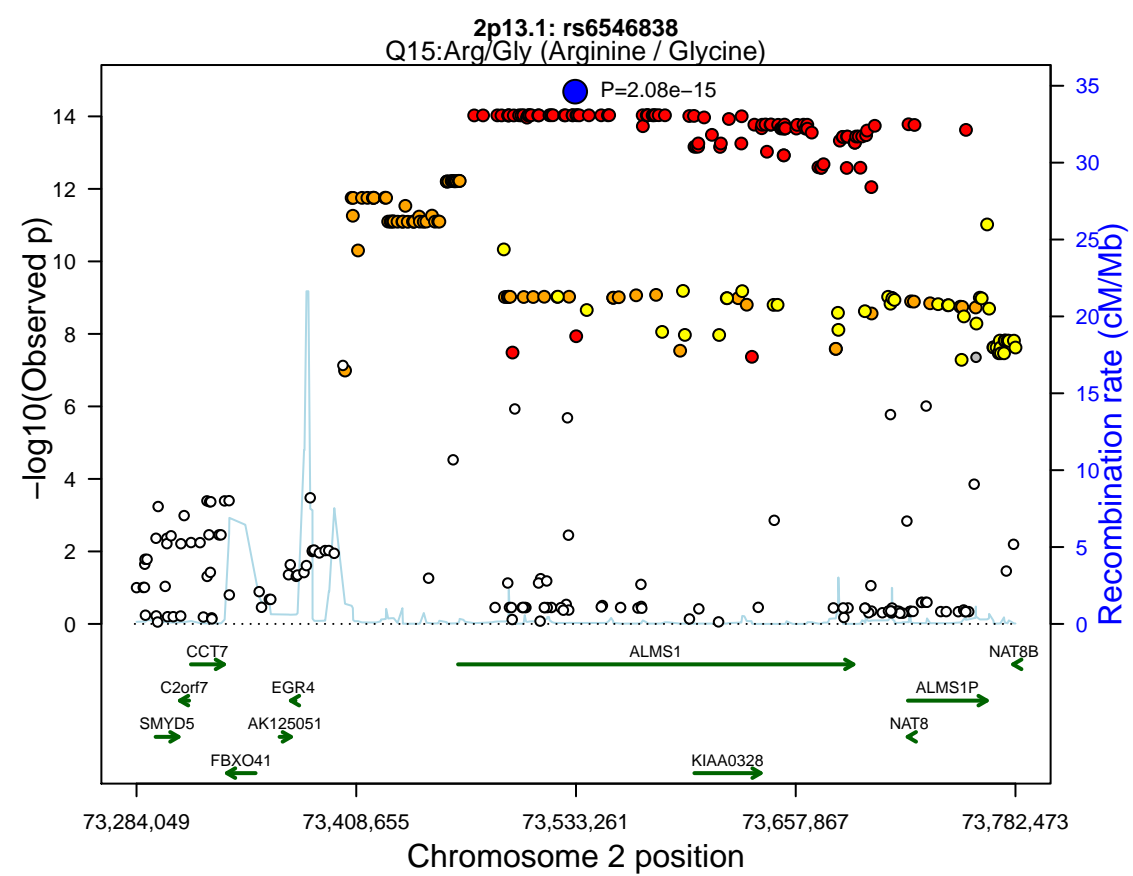

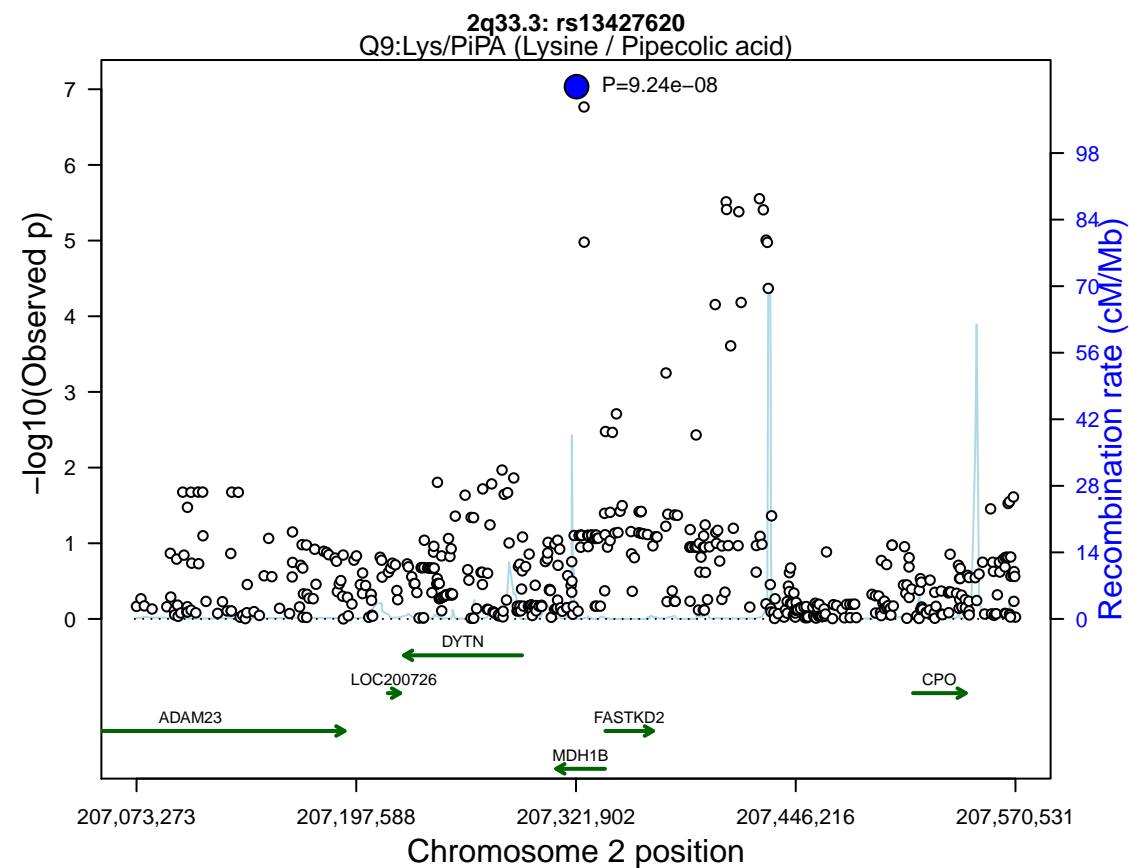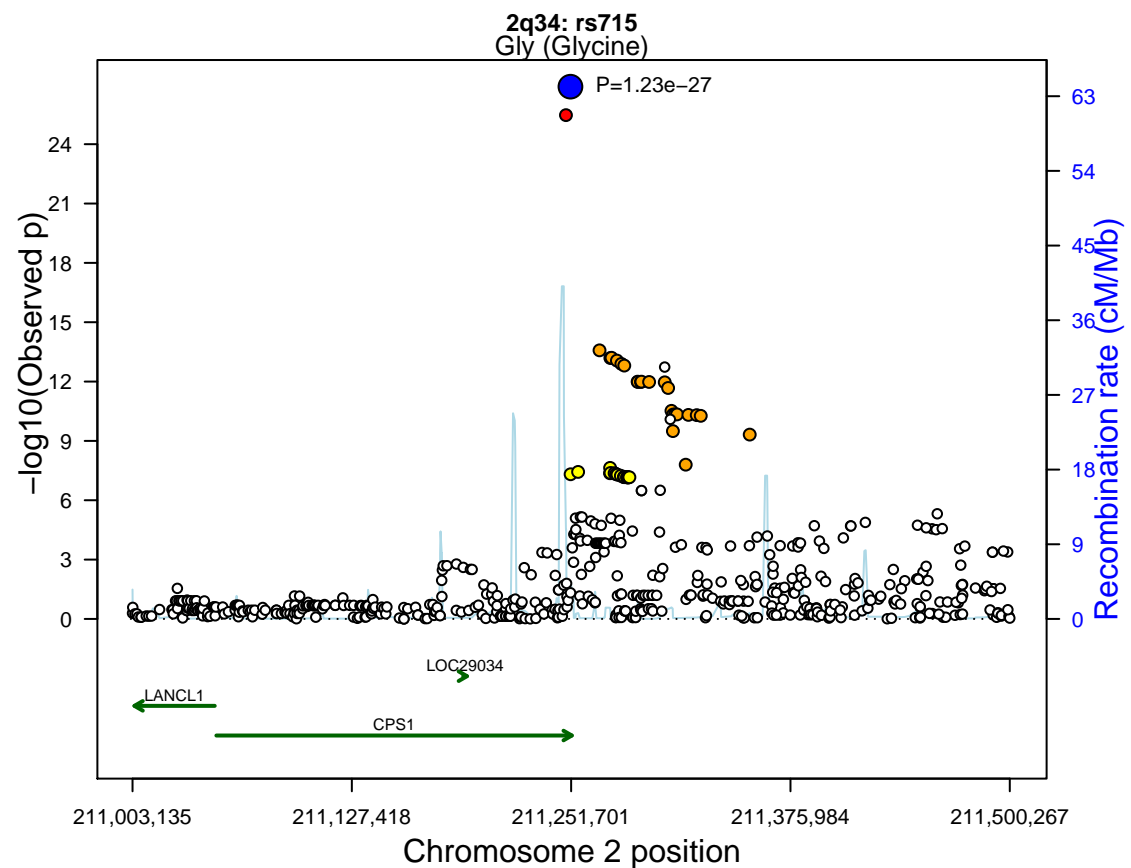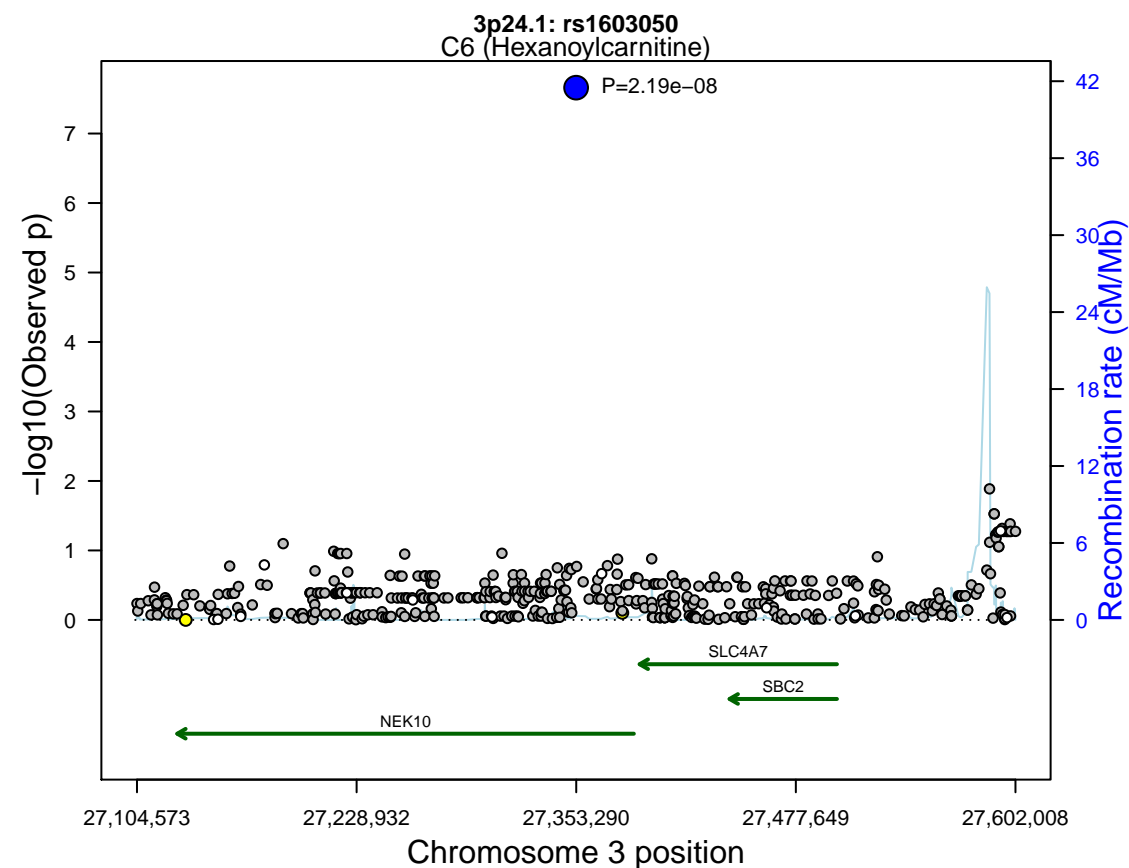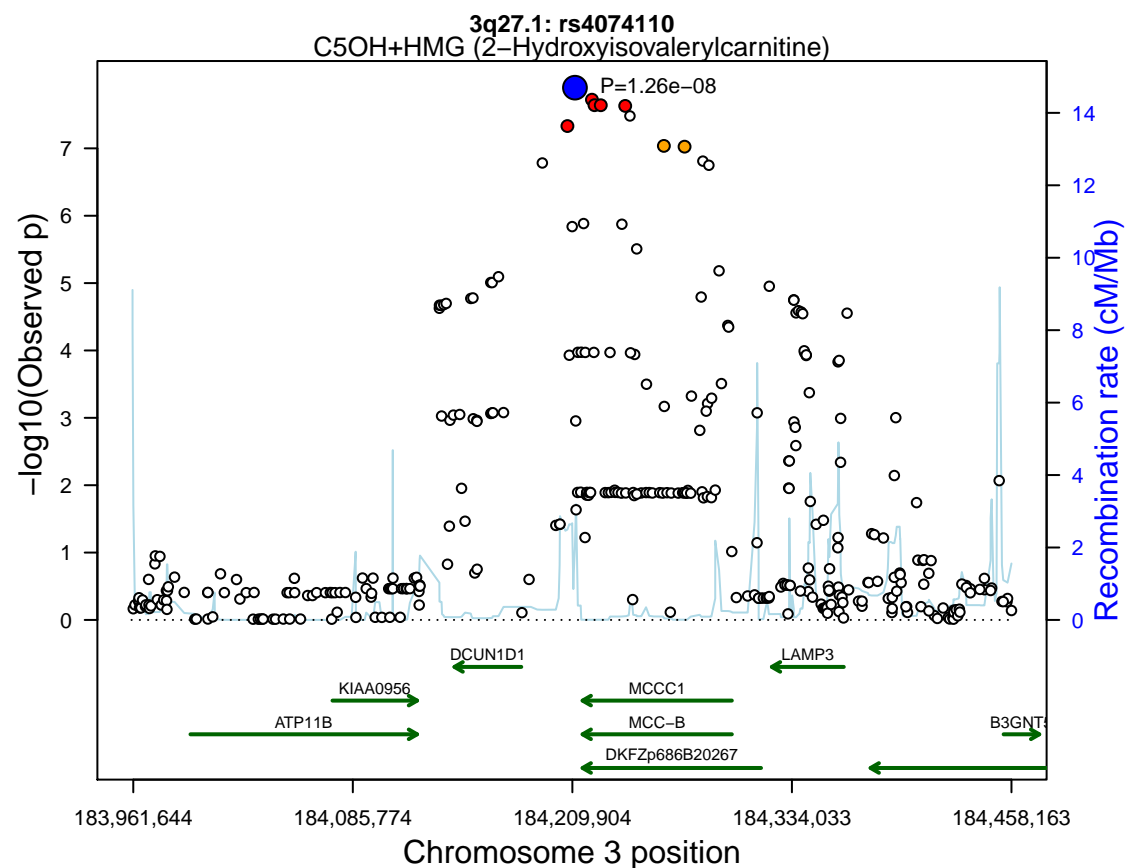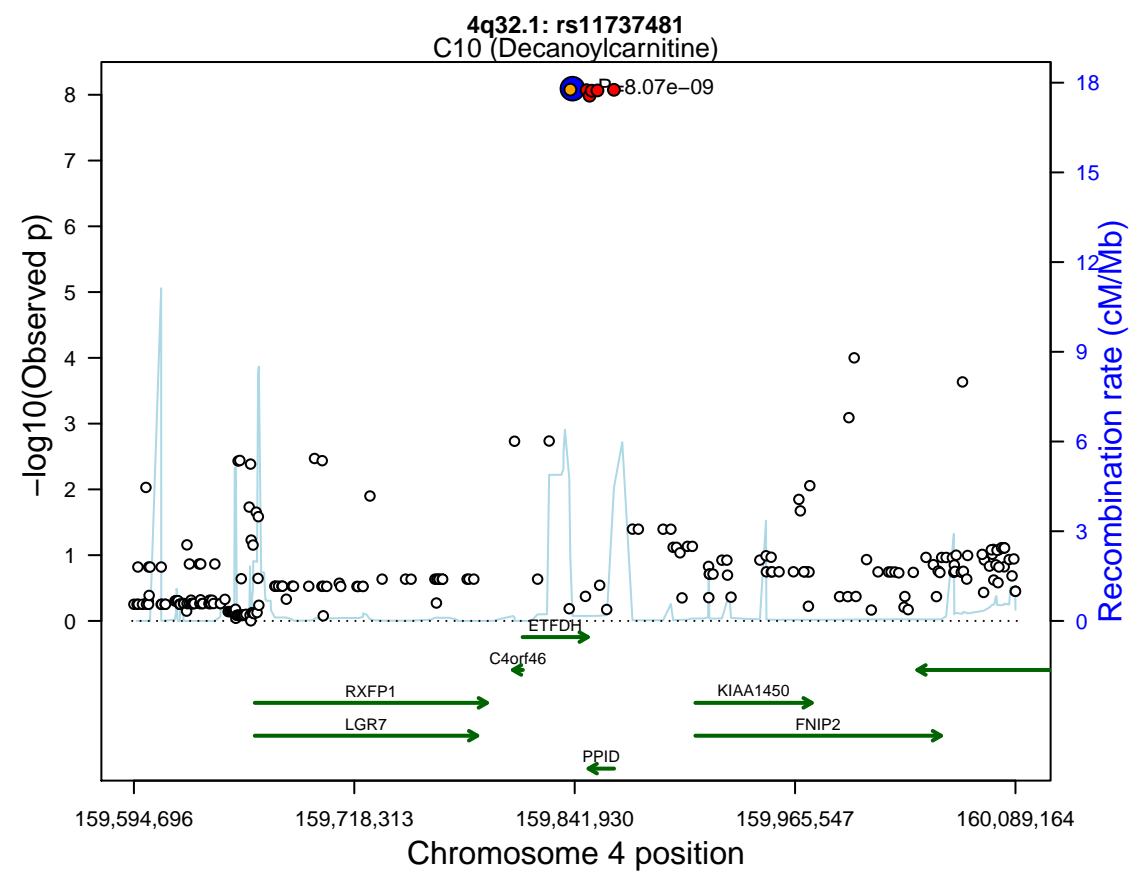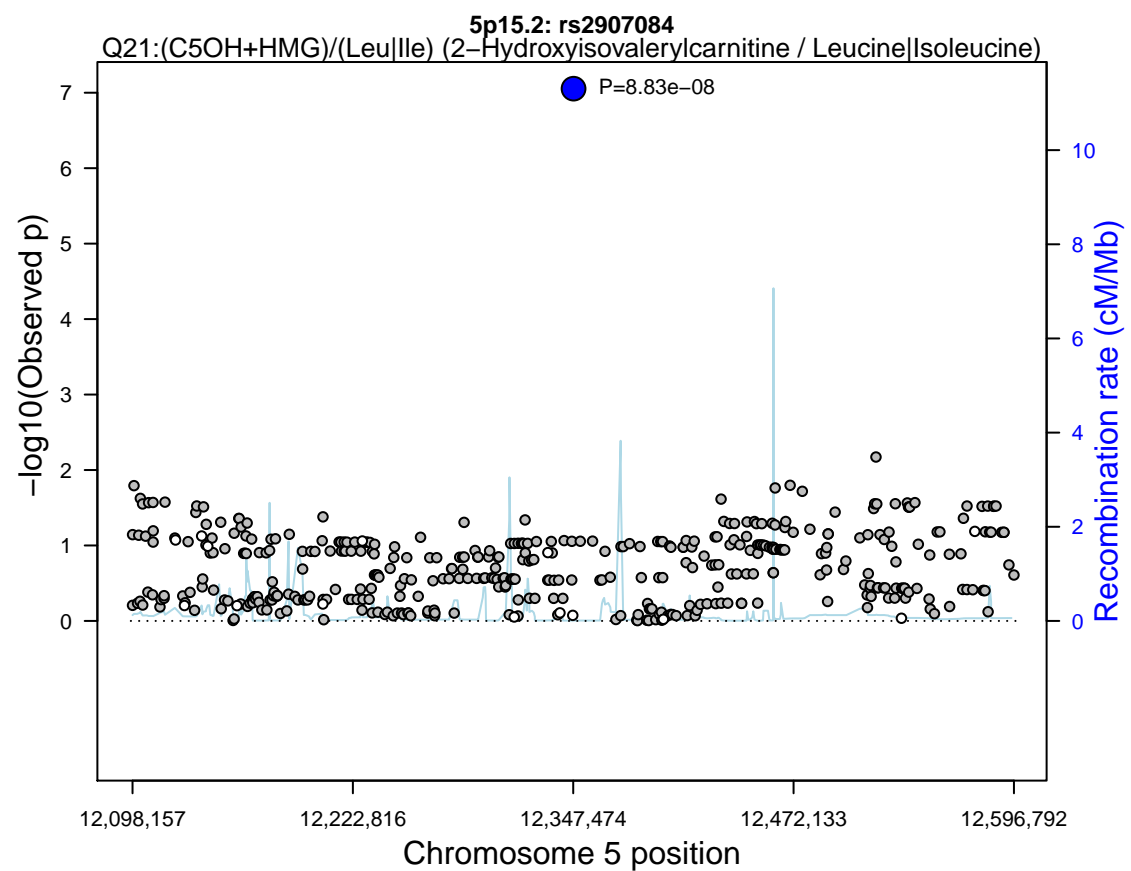

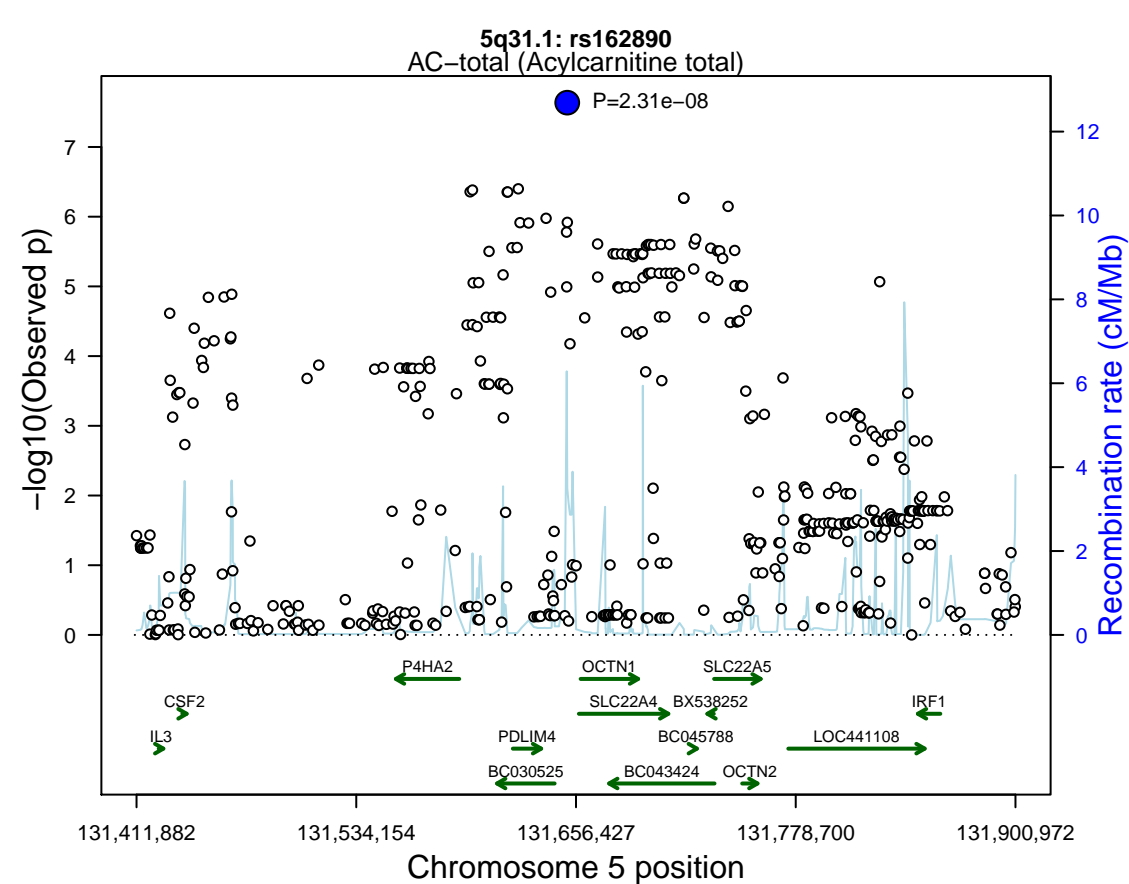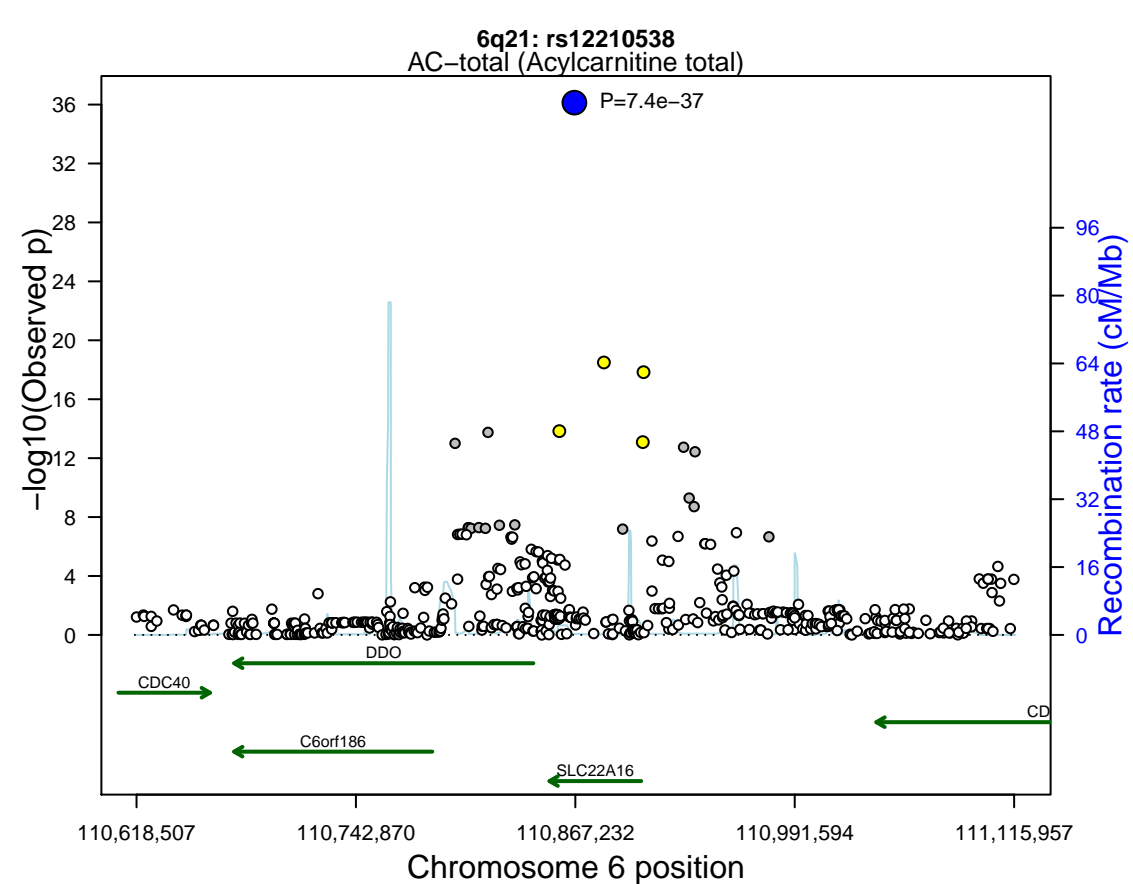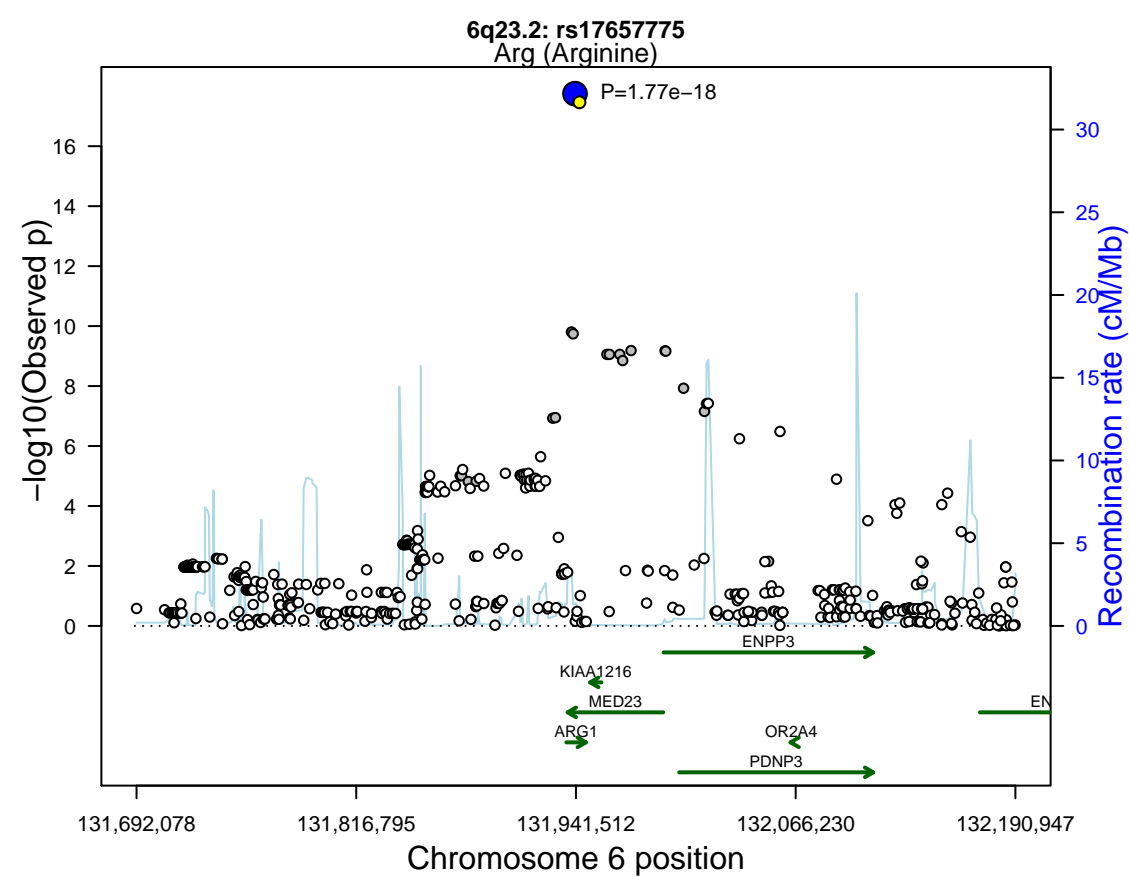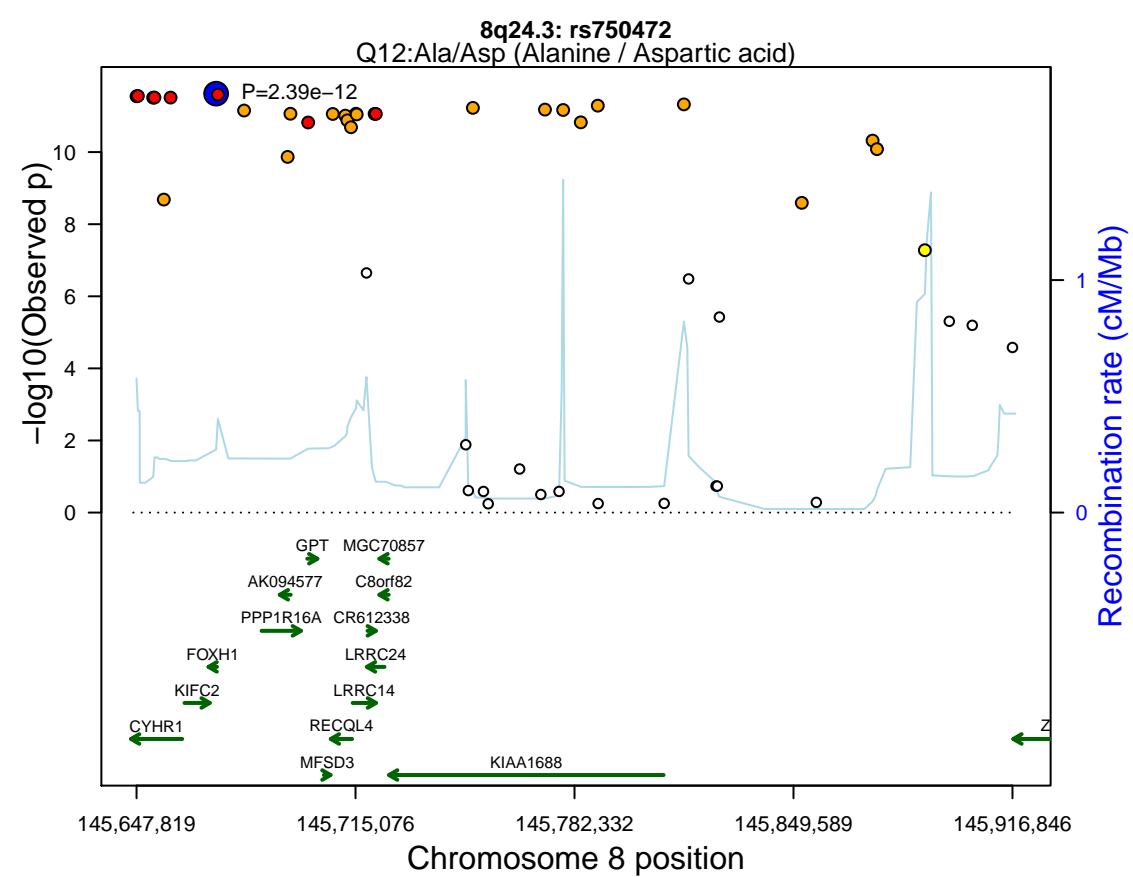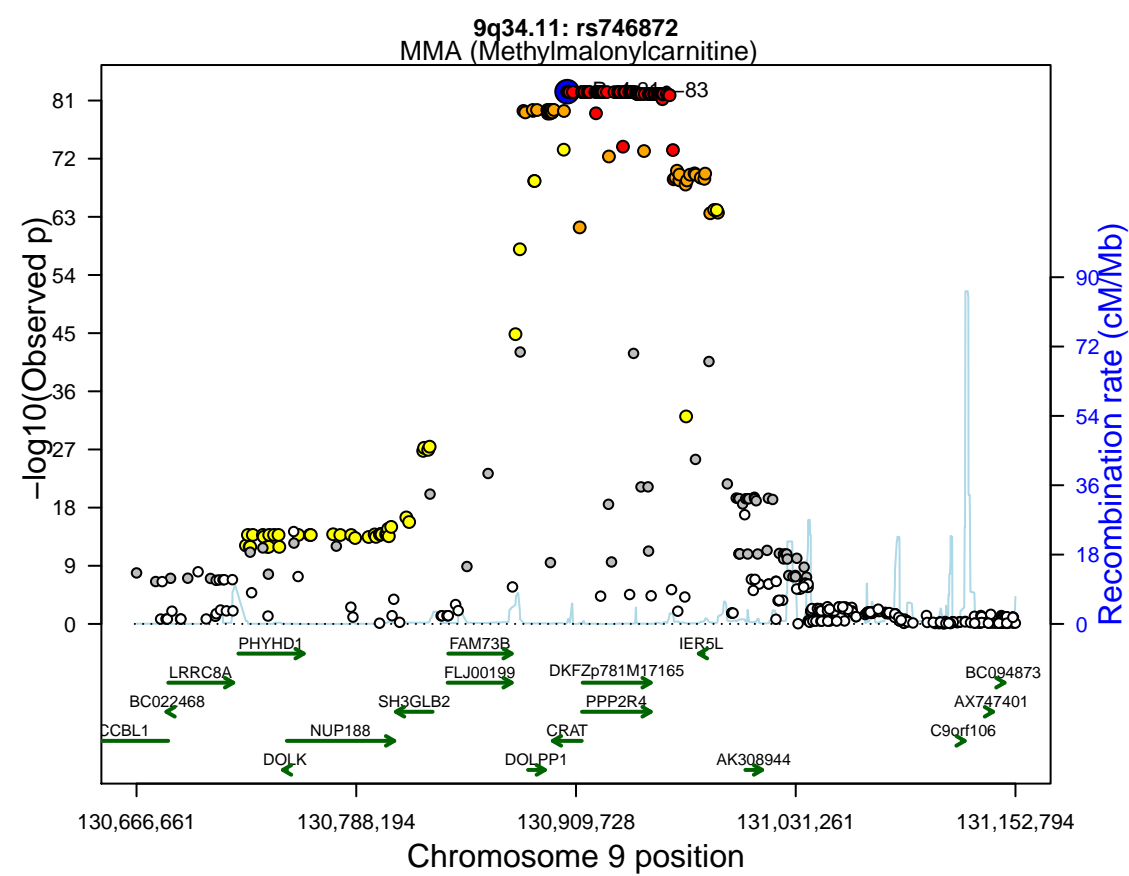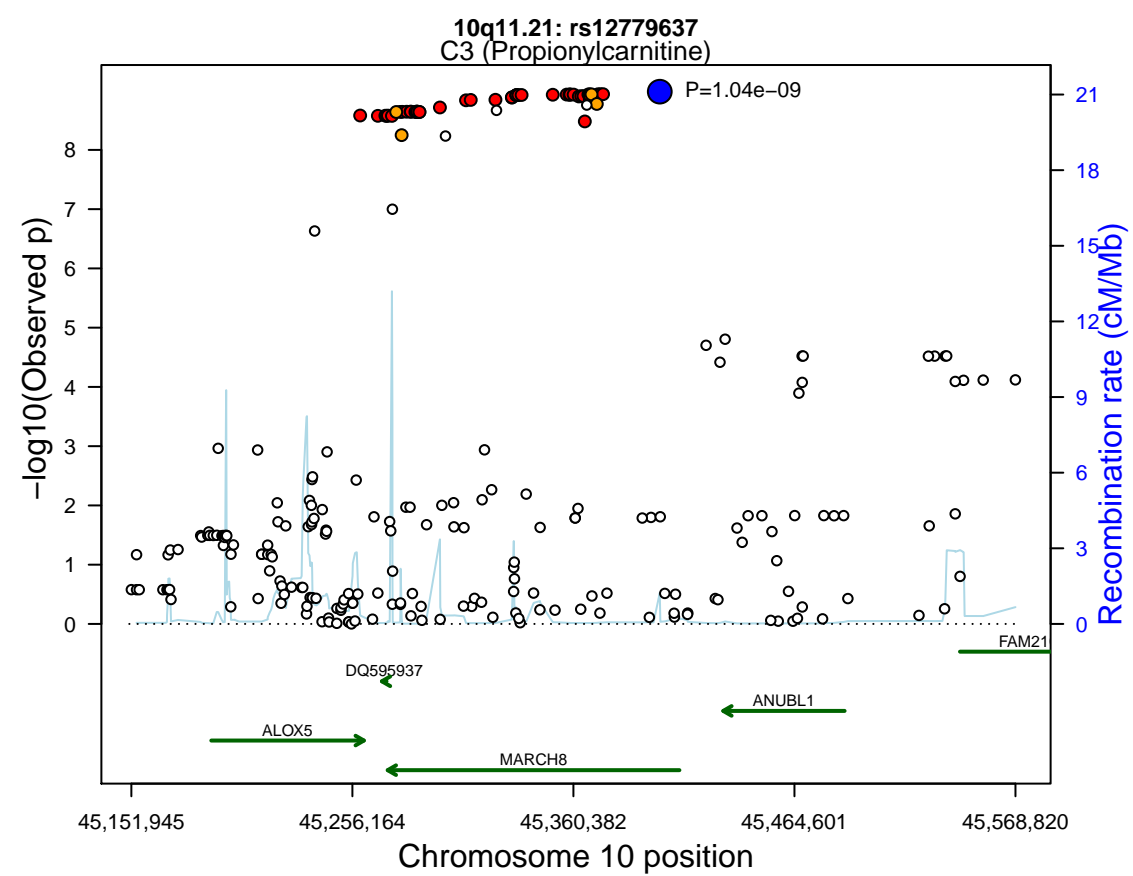

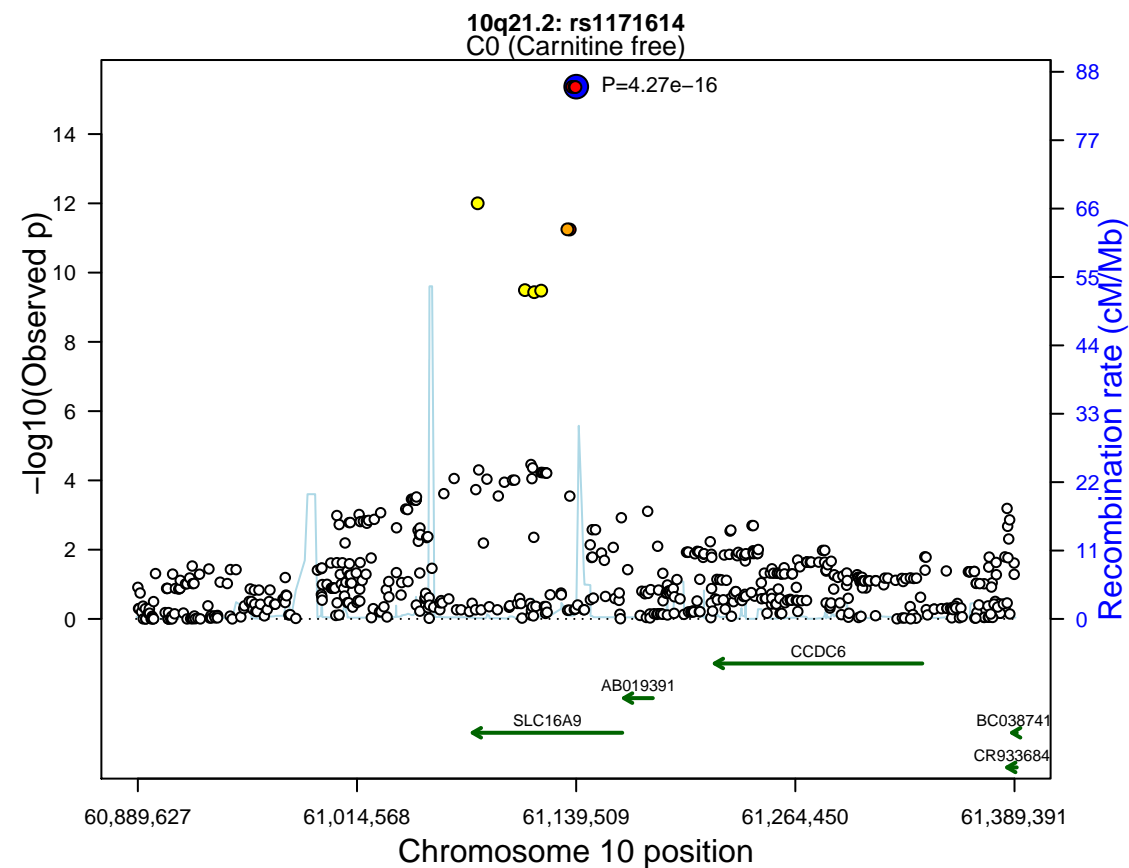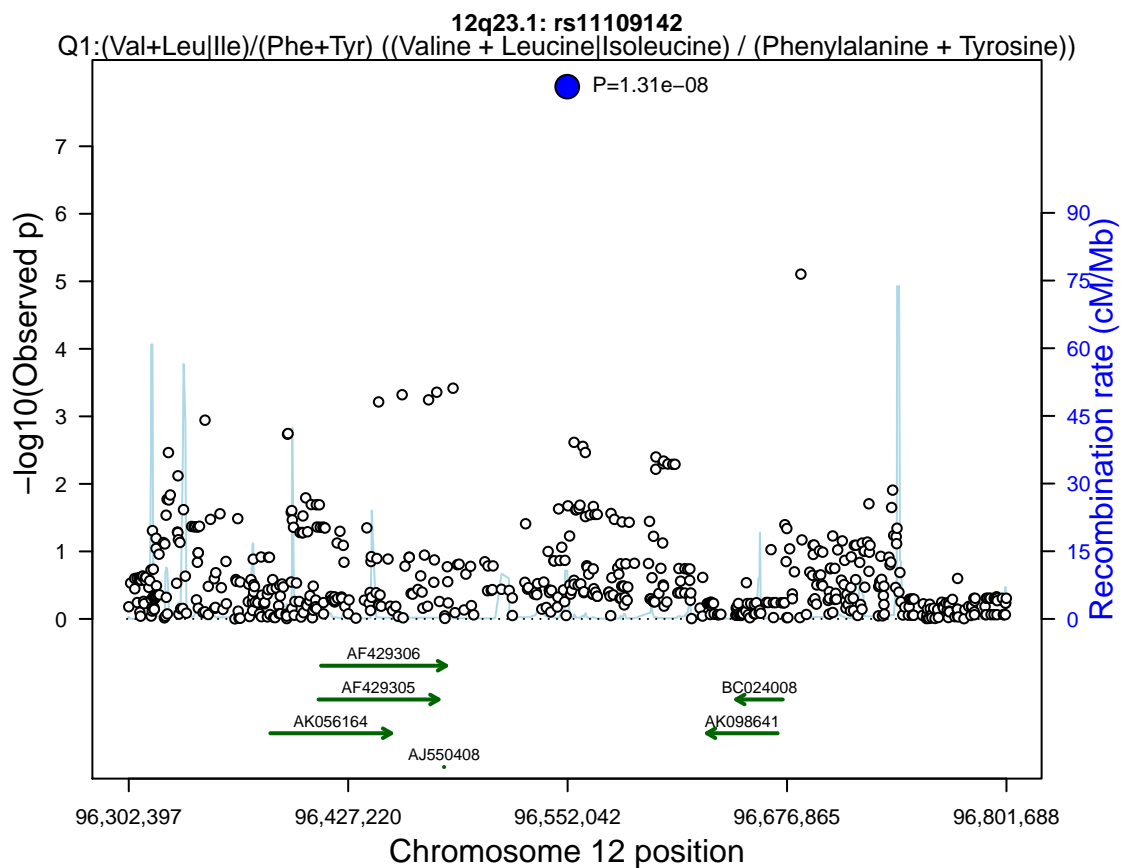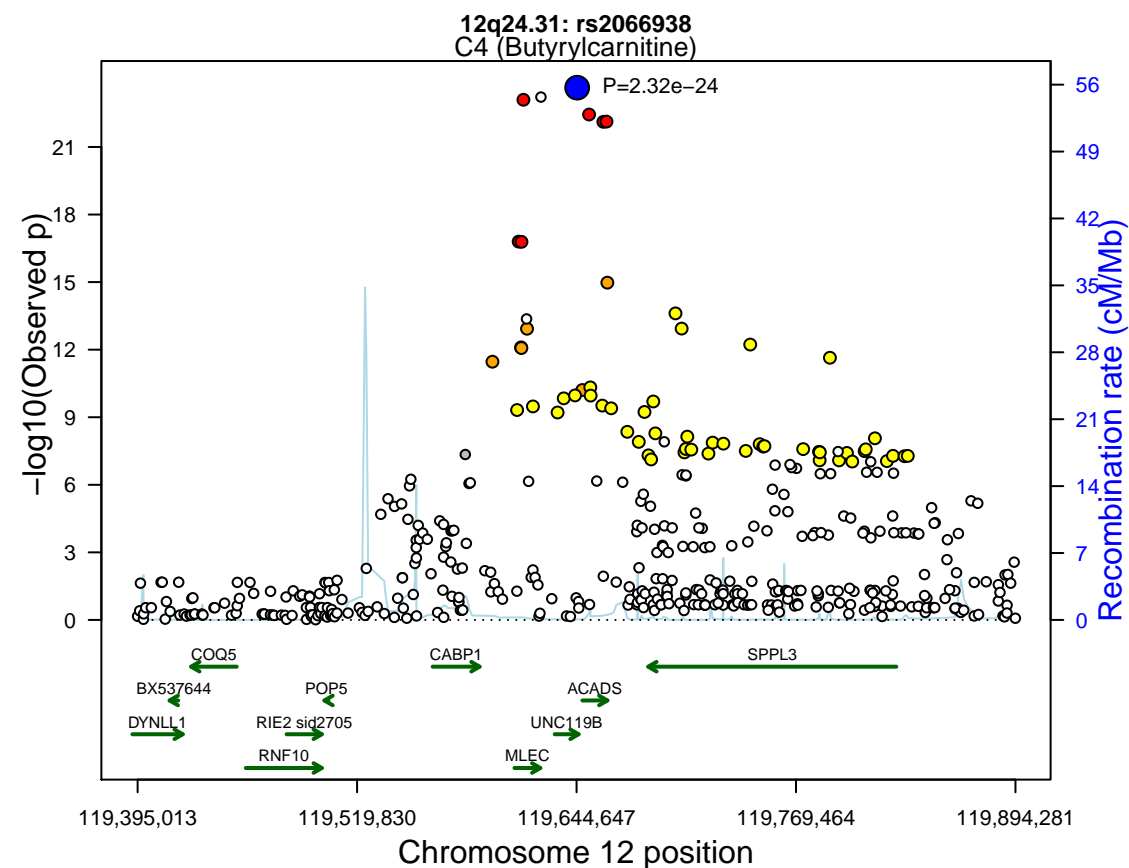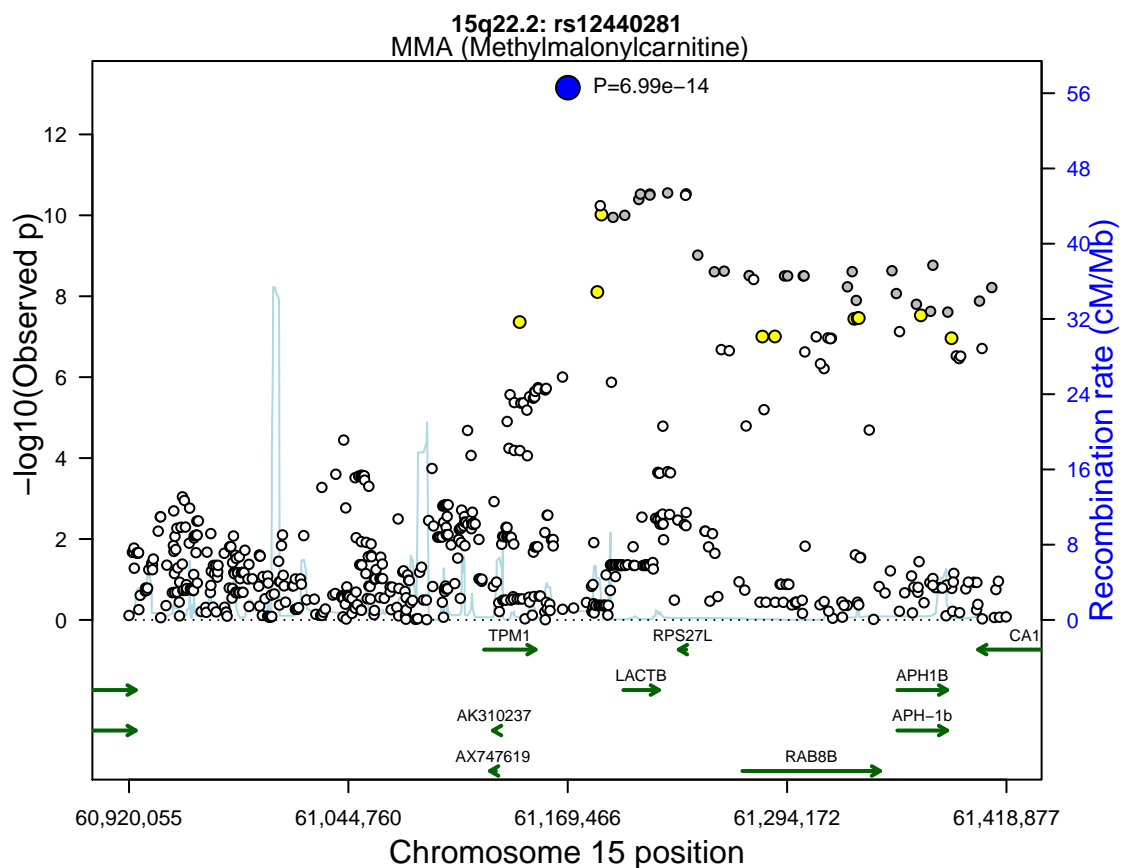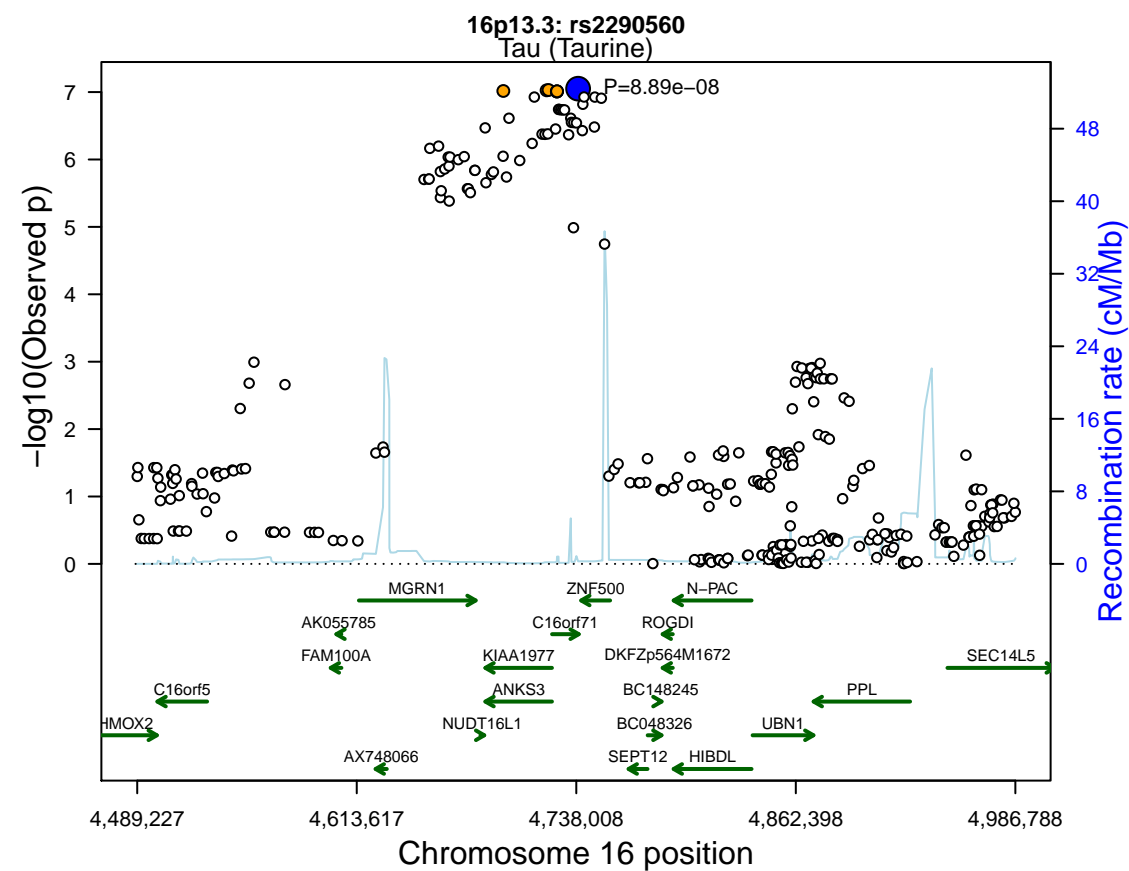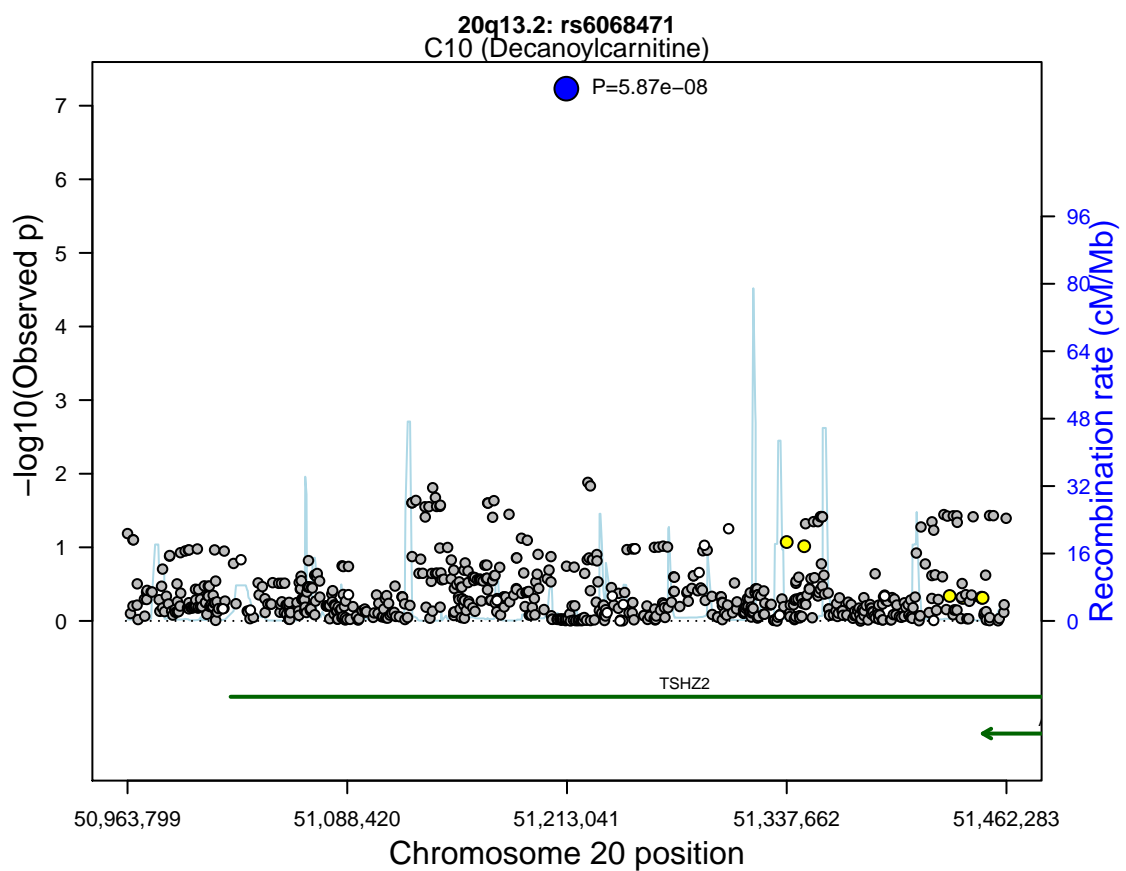

21q22.13: rs1571700  
C5OH+HMG (2-Hydroxyisovalerylcarnitine)

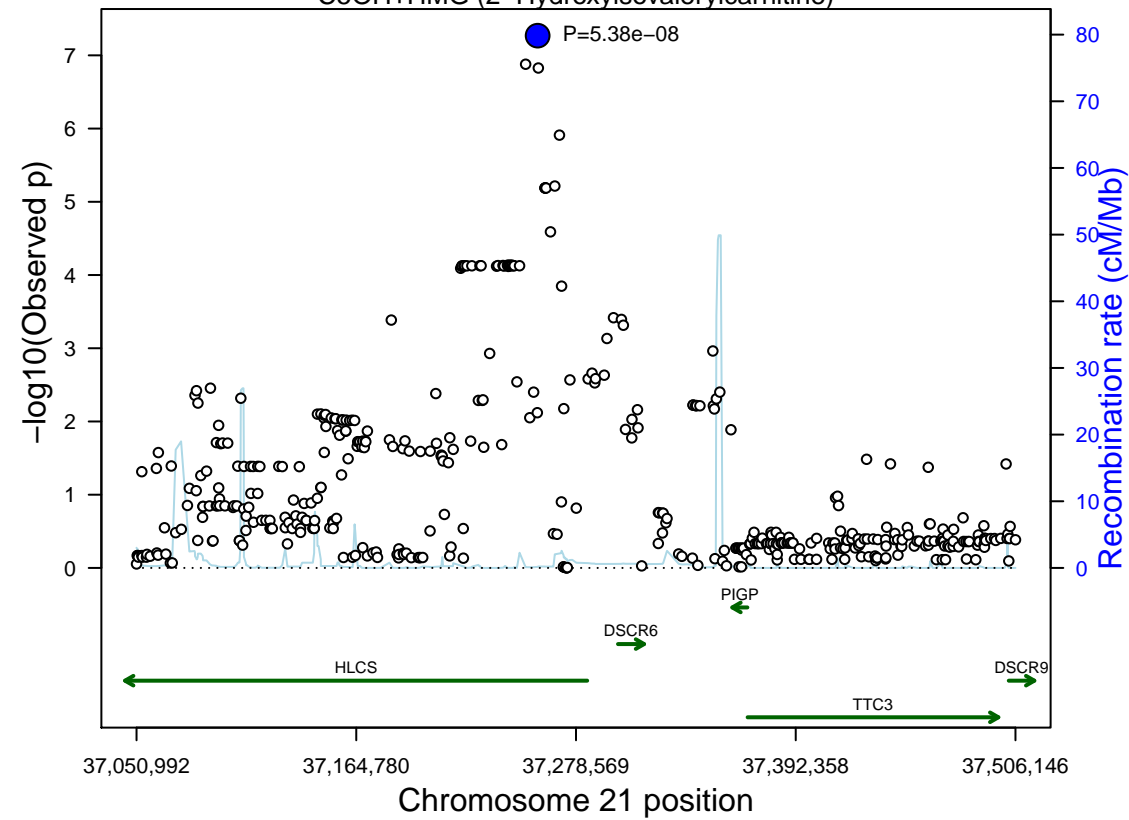

Supplement: S3 Fig — Regional association plots of all loci achieving p-values <1.0x10^-7^ for any of the metabolites and metabolite ratios. Association results are only shown for corresponding lead metabolites. (PDF) [file pgen.1005510.s003.pdf]

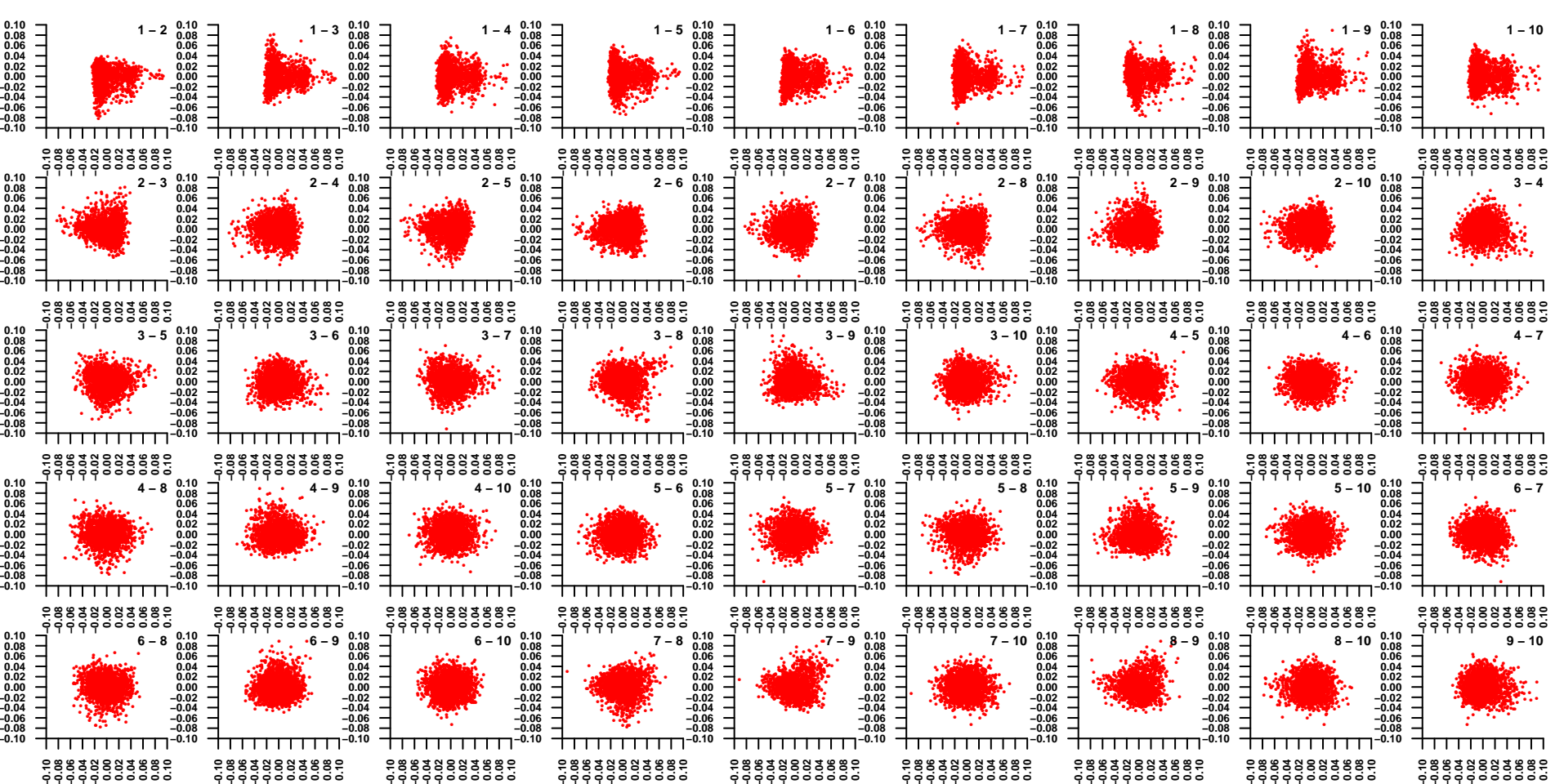

Supplement: S5 Fig — We present the first ten Principal Components for 2,107 LIFE-Heart samples included in our initial GWAS. (PDF) [file pgen.1005510.s005.pdf]

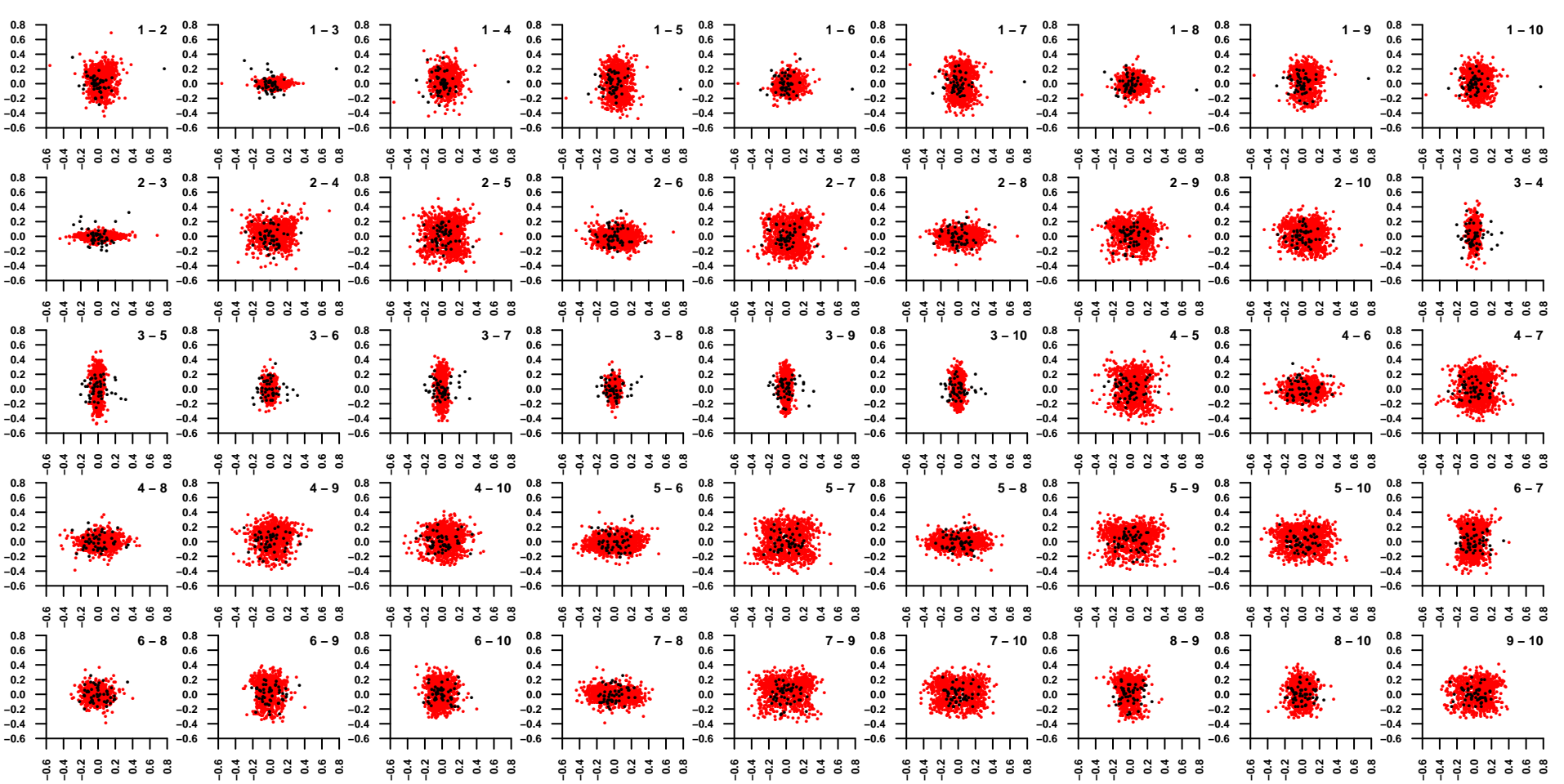

Supplement: S6 Fig — We present the first ten Principal Components of our replication sample of Sorbs individuals (red) in comparison to HapMap CEU (black). (PDF) [file pgen.1005510.s006.pdf]

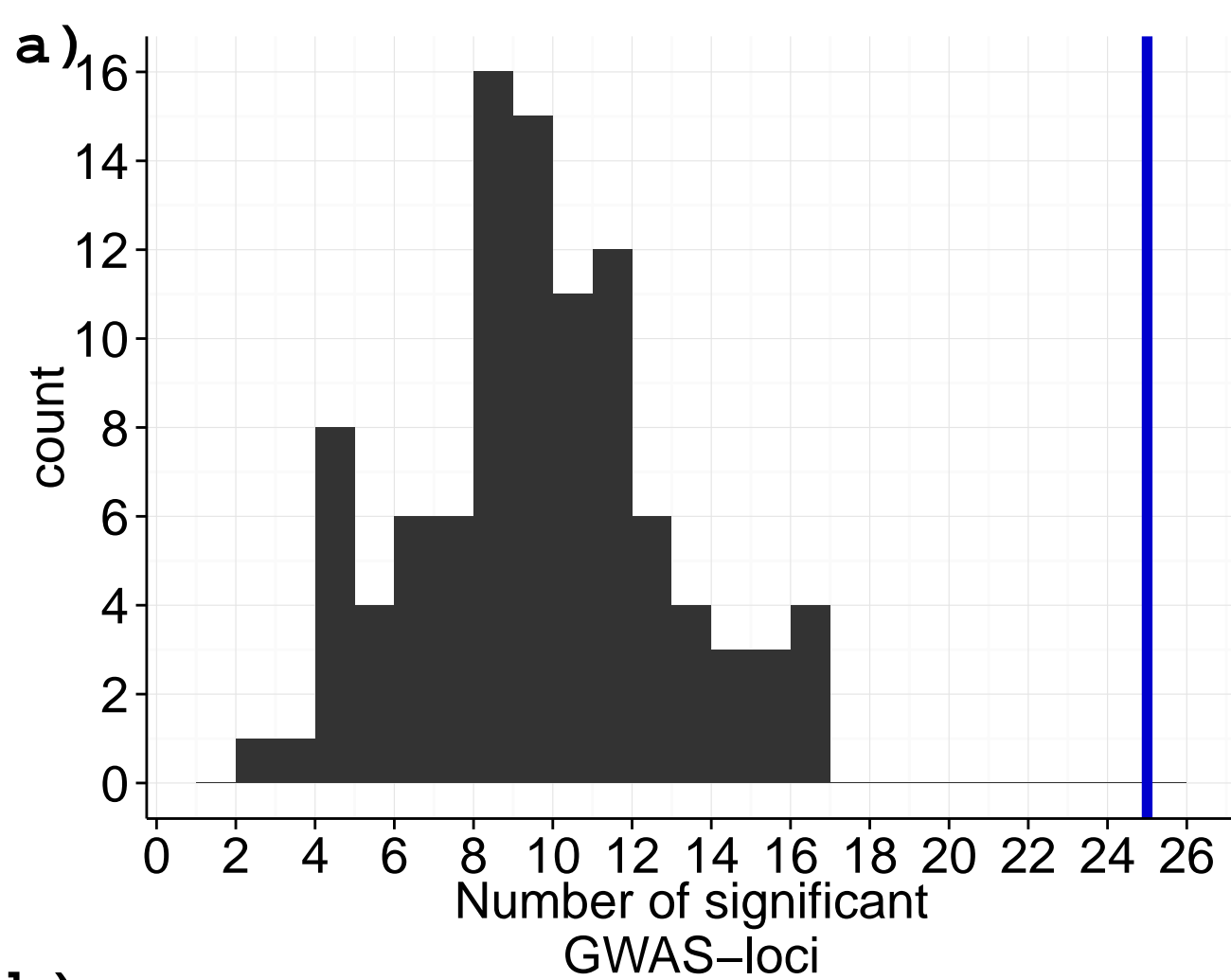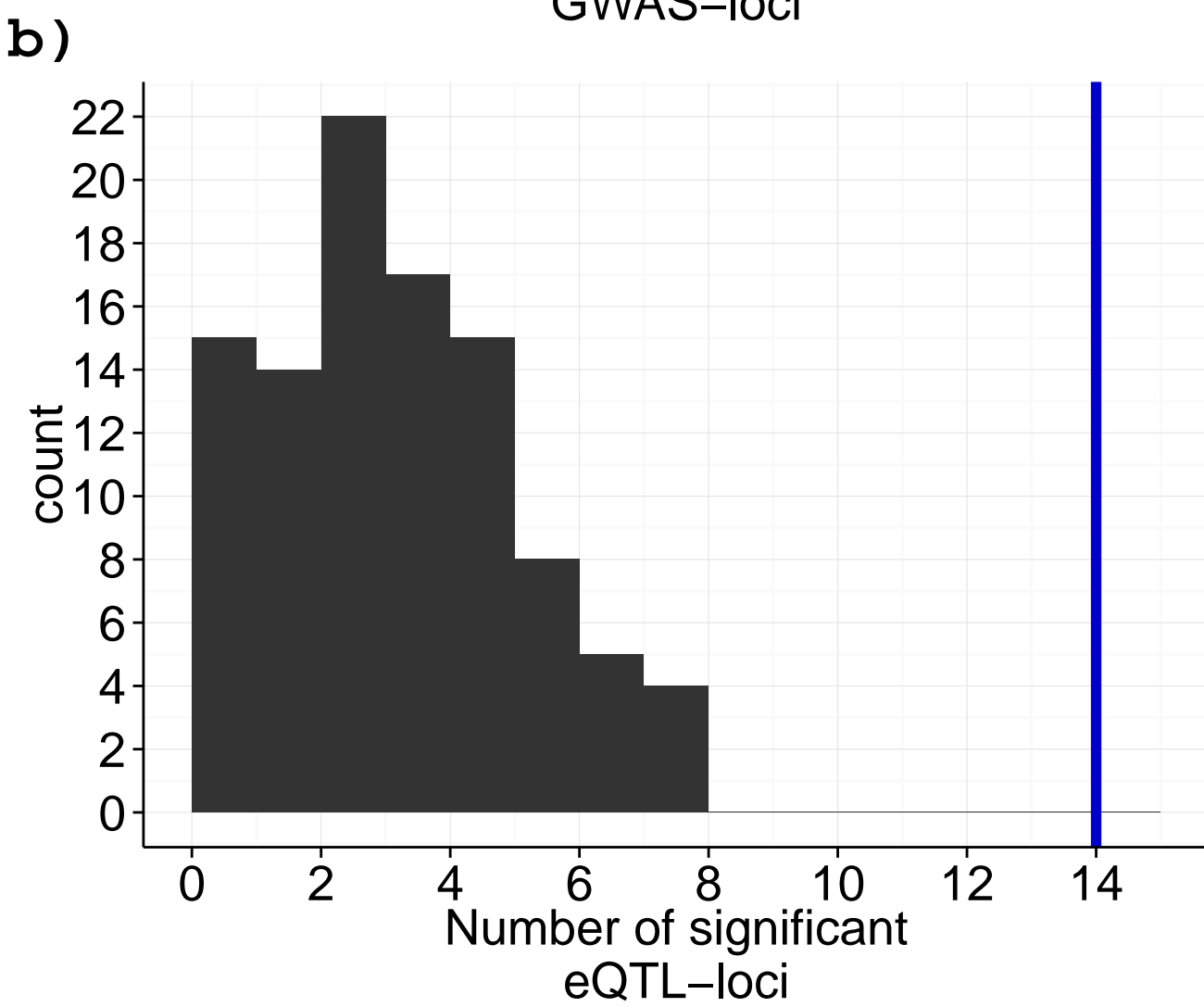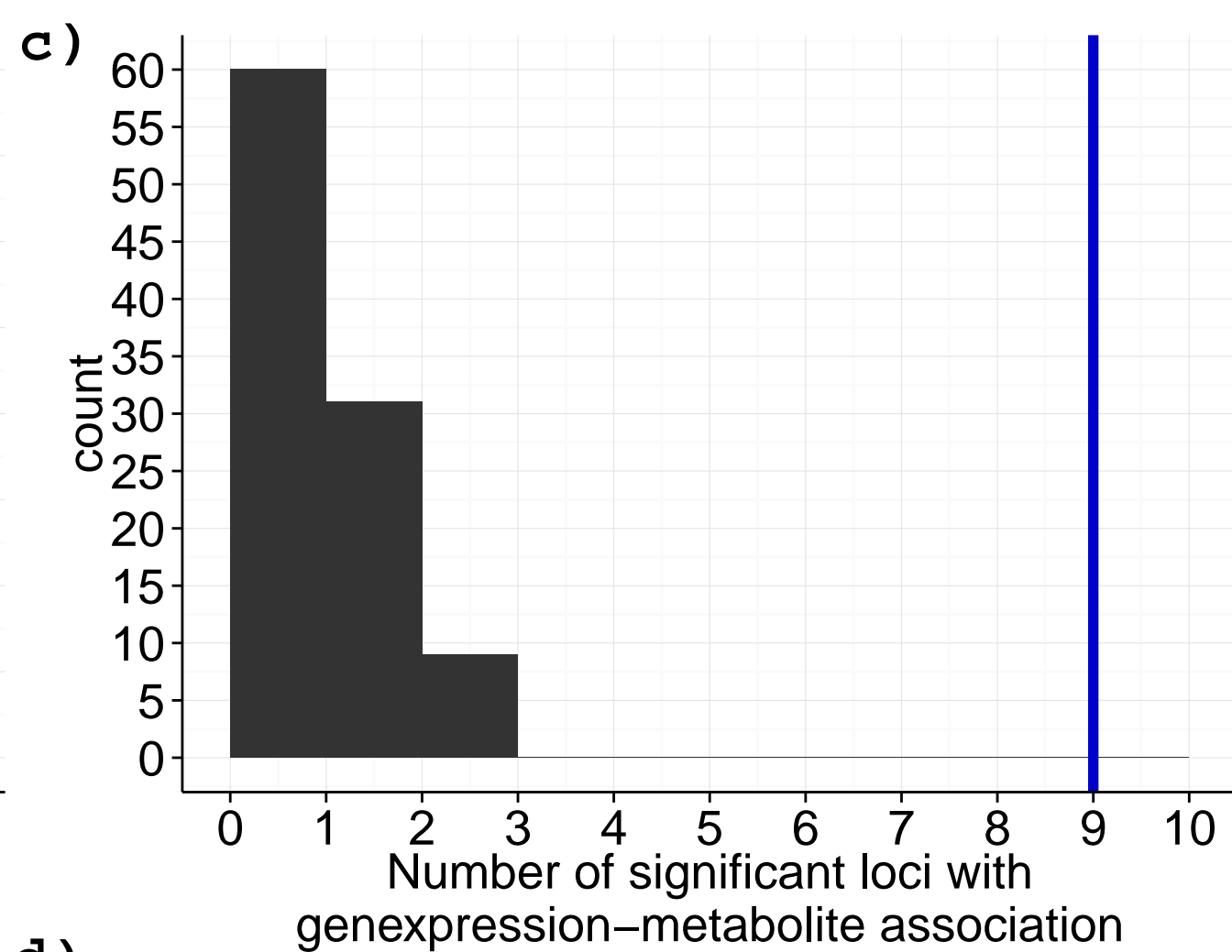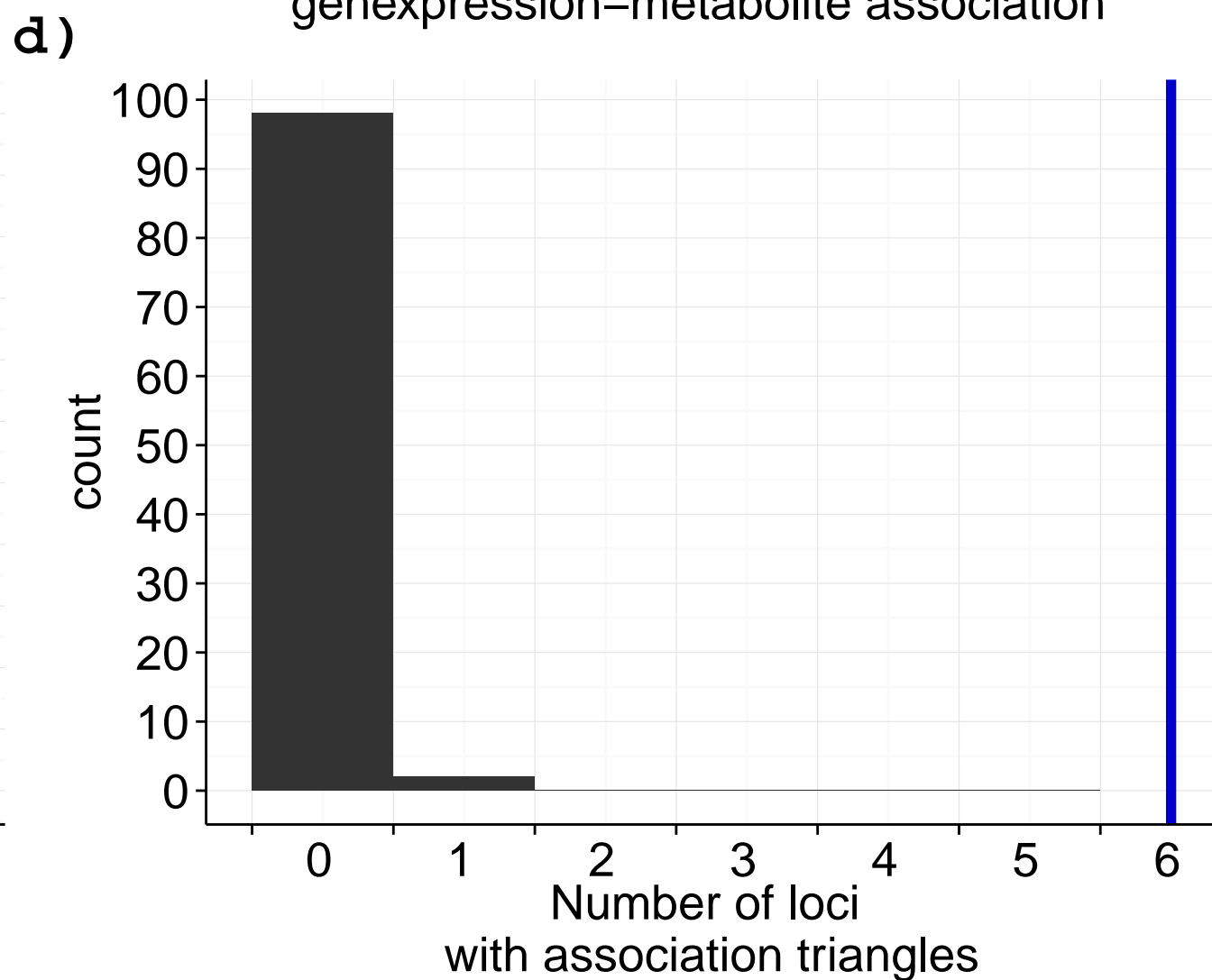

Supplement: S8 Fig — We performed 100 permutations including mQTL (sub-figure A), eQTL (sub-figure B), and gene-expression association analysis (sub-figure C) using the same cut-offs as in our original analysis. The aim was to simulate a null-distribution of association triangles (sub-figure D). For all analyses, we observed significantly more associations than expected by chance. Particularly, no association triangles were found in 98 permutations while only one triangle was found in two permutations. In our original analysis, we observed six triangles. (PDF) [file pgen.1005510.s008.pdf]
