# Supplementary material for: Integration of Genome-Wide SNP Data and Gene-Expression Profiles Reveals Six Novel Loci and Regulatory Mechanisms for Amino Acids and Acylcarnitines in Whole Blood
Source: PLoS Genet. 2015 Sep 24;11(9):e1005510. doi: 10.1371/journal.pgen.1005510 (PMC4581711; doi:10.1371/journal.pgen.1005510)
Supplement: S2 Fig — Quantile-quantile plots of (–log10)-p-values for our genome-wide association study of amino acids and acylcarnitines in the LIFE Leipzig Heart study. Post-analysis quality control was applied prior to plotting the results. (PDF) [file pgen.1005510.s002.pdf]

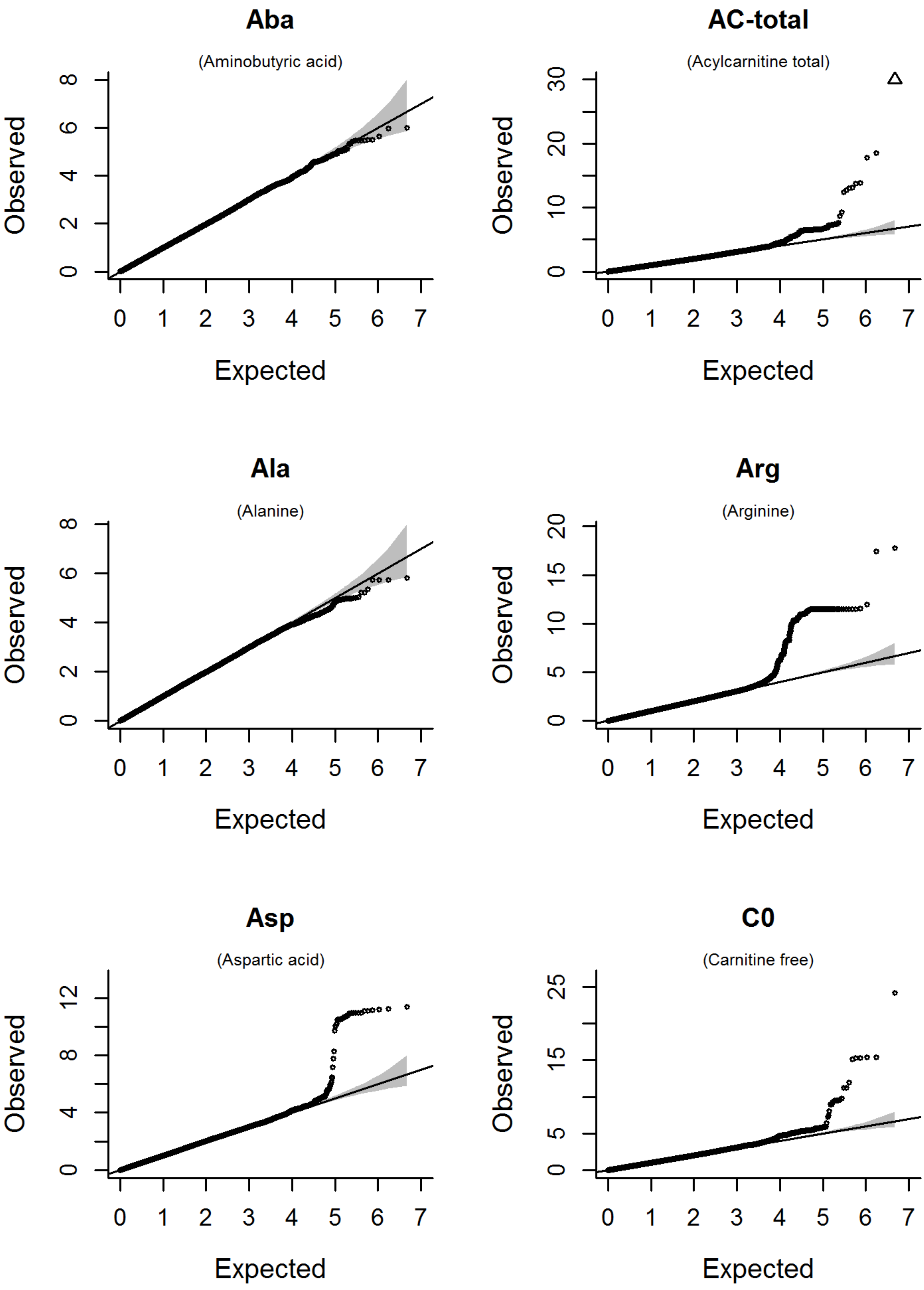
Aba
(Aminobutyric acid)
AC-total
(Acylcarnitine total)
Ala
(Alanine)
Arg
(Arginine)
Asp
(Aspartic acid)
C0
(Carnitine free)
Observed
Expected

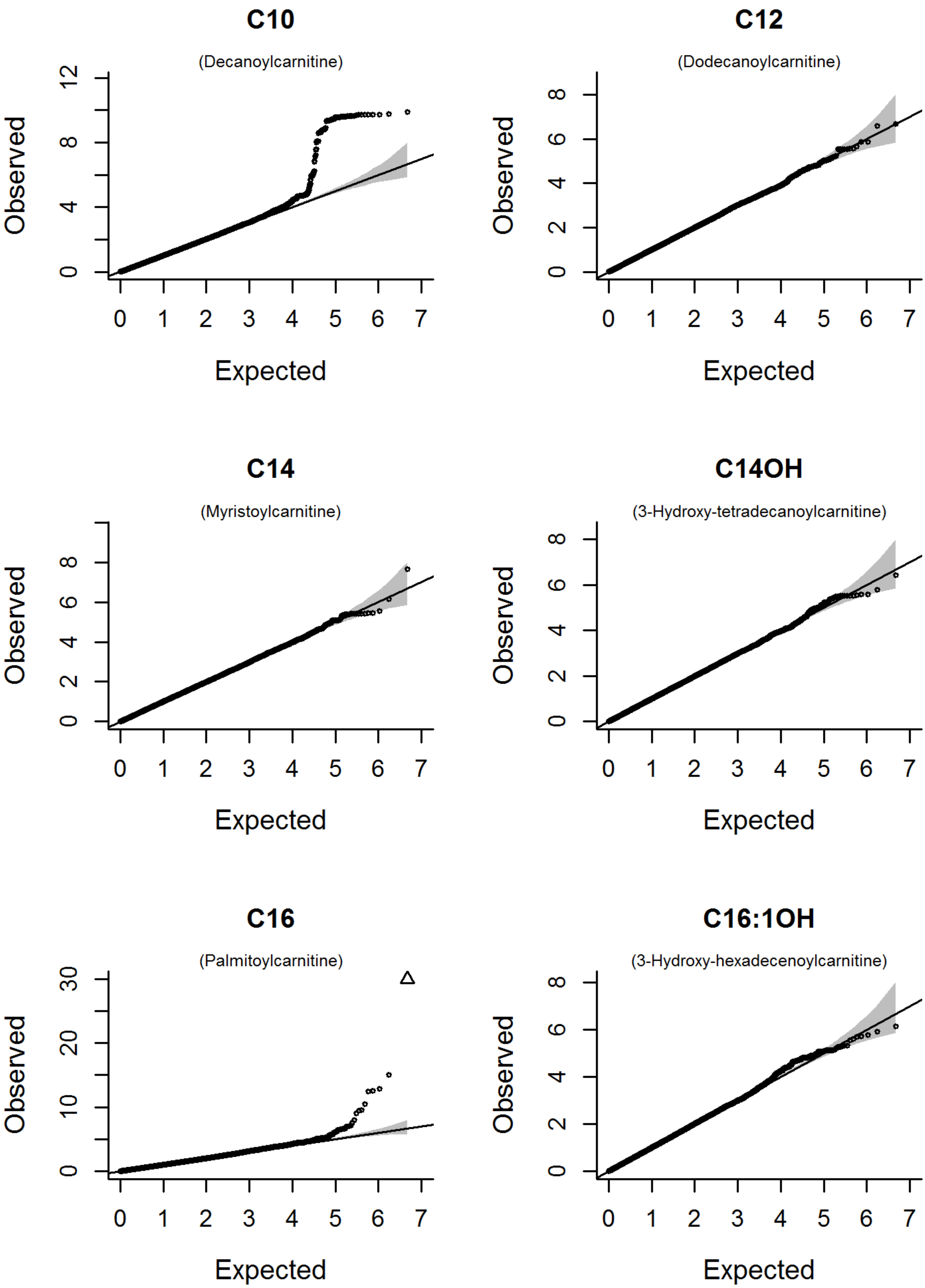
C10
(Decanoylcarnitine)
C12
(Dodecanoylcarnitine)
C14
(Myristoylcarnitine)
C14OH
(3-Hydroxy-tetradecanoylcarnitine)
C16
(Palmitoylcarnitine)
C16:1OH
(3-Hydroxy-hexadecenoylcarnitine)
Observed
Expected

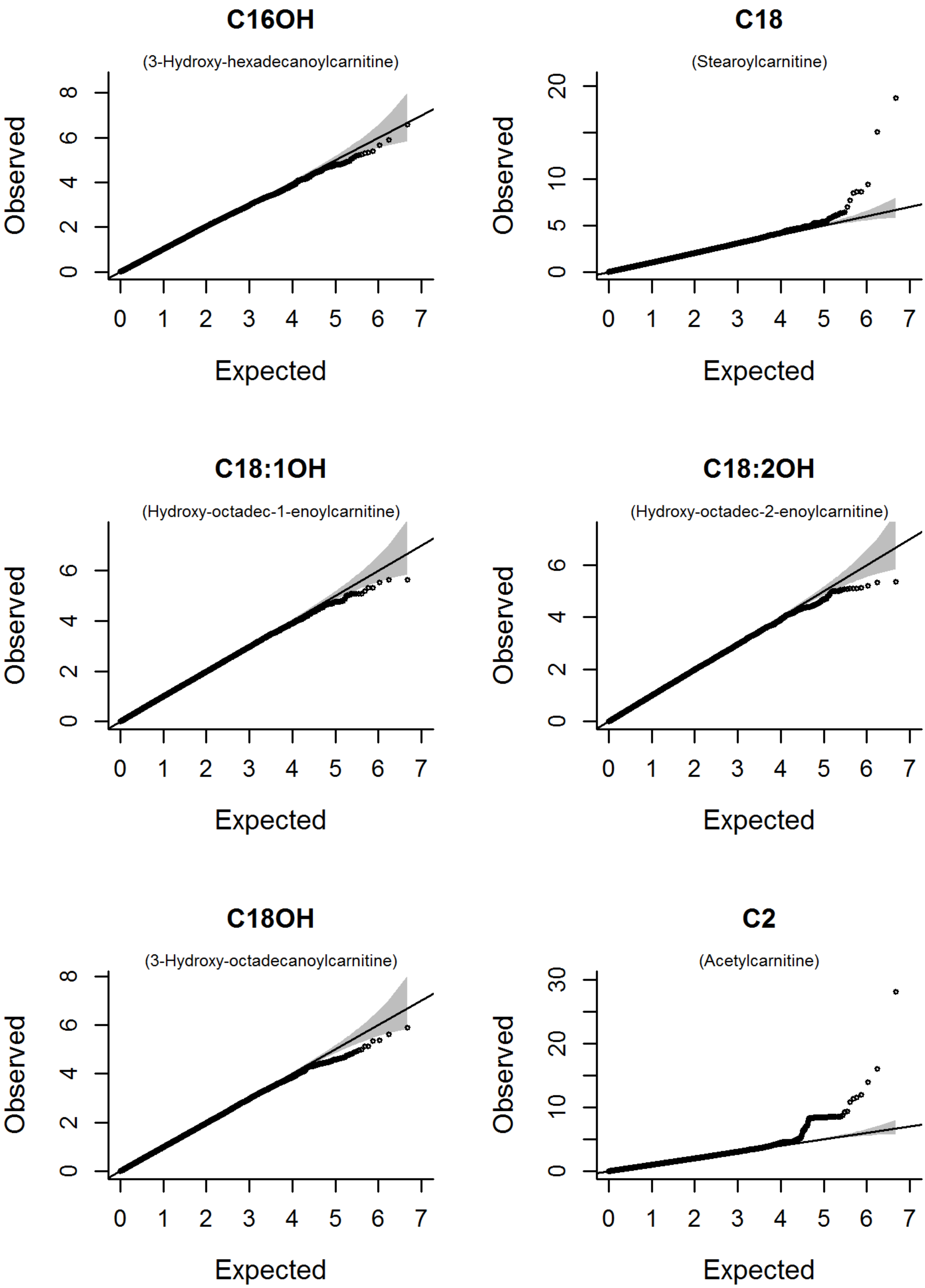
C16OH
(3-Hydroxy-hexadecanoylcarnitine)
Observed
Expected
C18
(Stearoylcarnitine)
Observed
Expected
C18:1OH
(Hydroxy-octadec-1-enoylcarnitine)
Observed
Expected
C18:2OH
(Hydroxy-octadec-2-enoylcarnitine)
Observed
Expected
C18OH
(3-Hydroxy-octadecanoylcarnitine)
Observed
Expected
C2
(Acetylcarnitine)
Observed
Expected

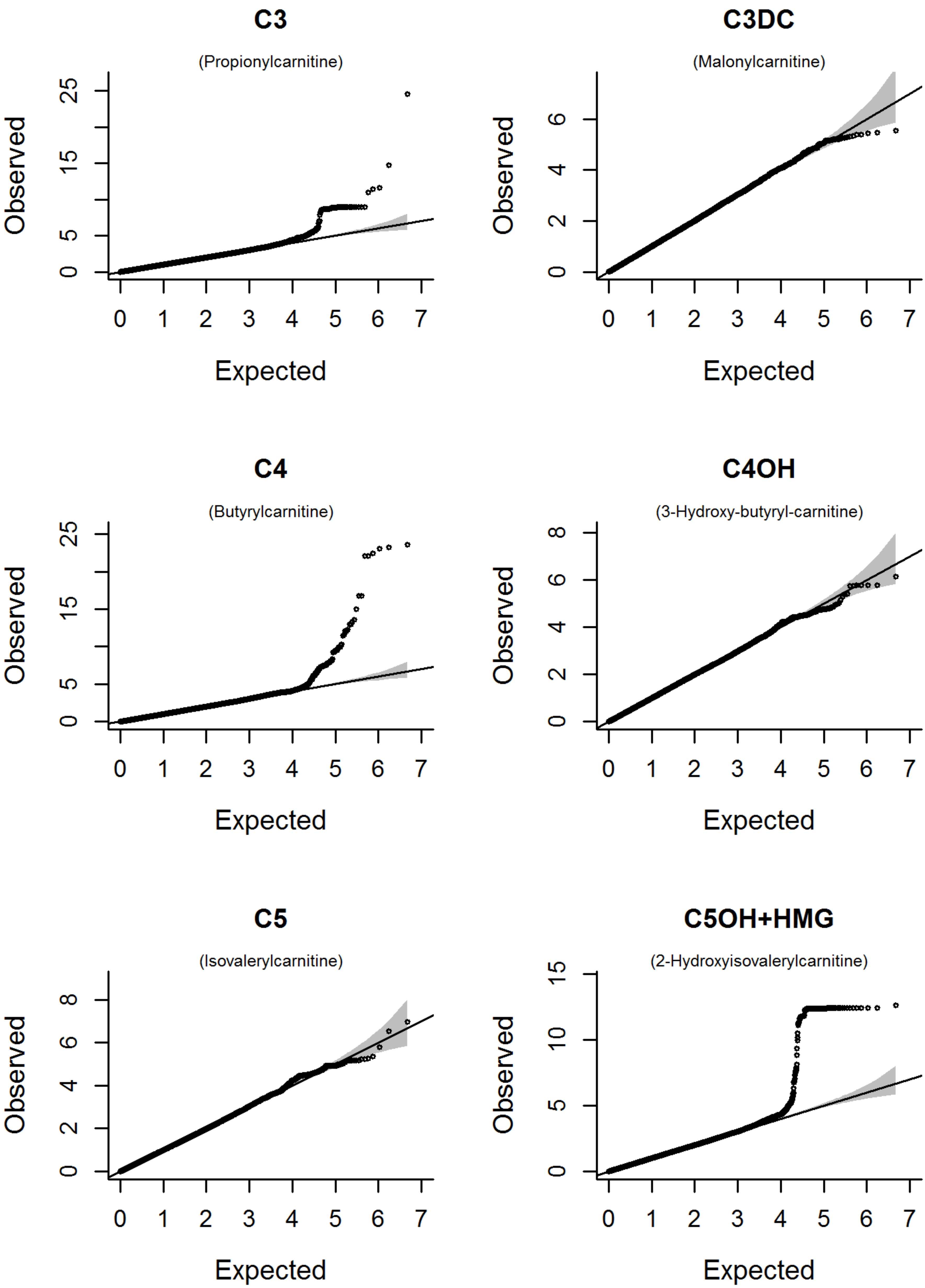
C3
(Propionylcarnitine)
C3DC
(Malonylcarnitine)
C4
(Butyrylcarnitine)
C4OH
(3-Hydroxy-butyryl-carnitine)
C5
(Isovalerylcarnitine)
C5OH+HMG
(2-Hydroxyisovalerylcarnitine)
Observed
Expected
0 1 2 3 4 5 6 7
0 5 15 25
0 2 4 6
0 2 4 6 8
0 5 10 15

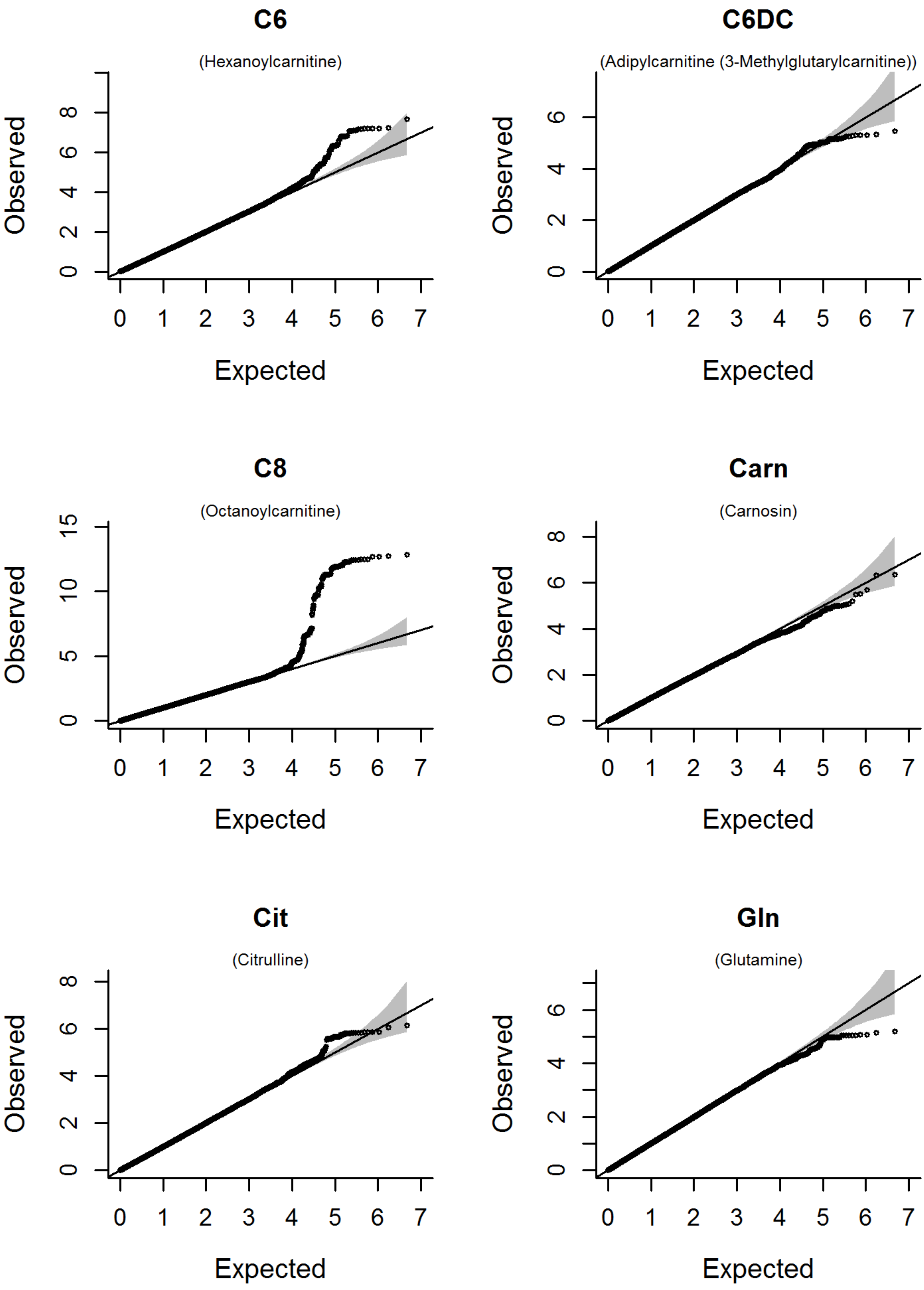
C6
(Hexanoylcarnitine)
C6DC
(Adipylcarnitine (3-Methylglutarylcarnitine))
C8
(Octanoylcarnitine)
Carn
(Carnosin)
Cit
(Citrulline)
Gln
(Glutamine)
Observed
Expected

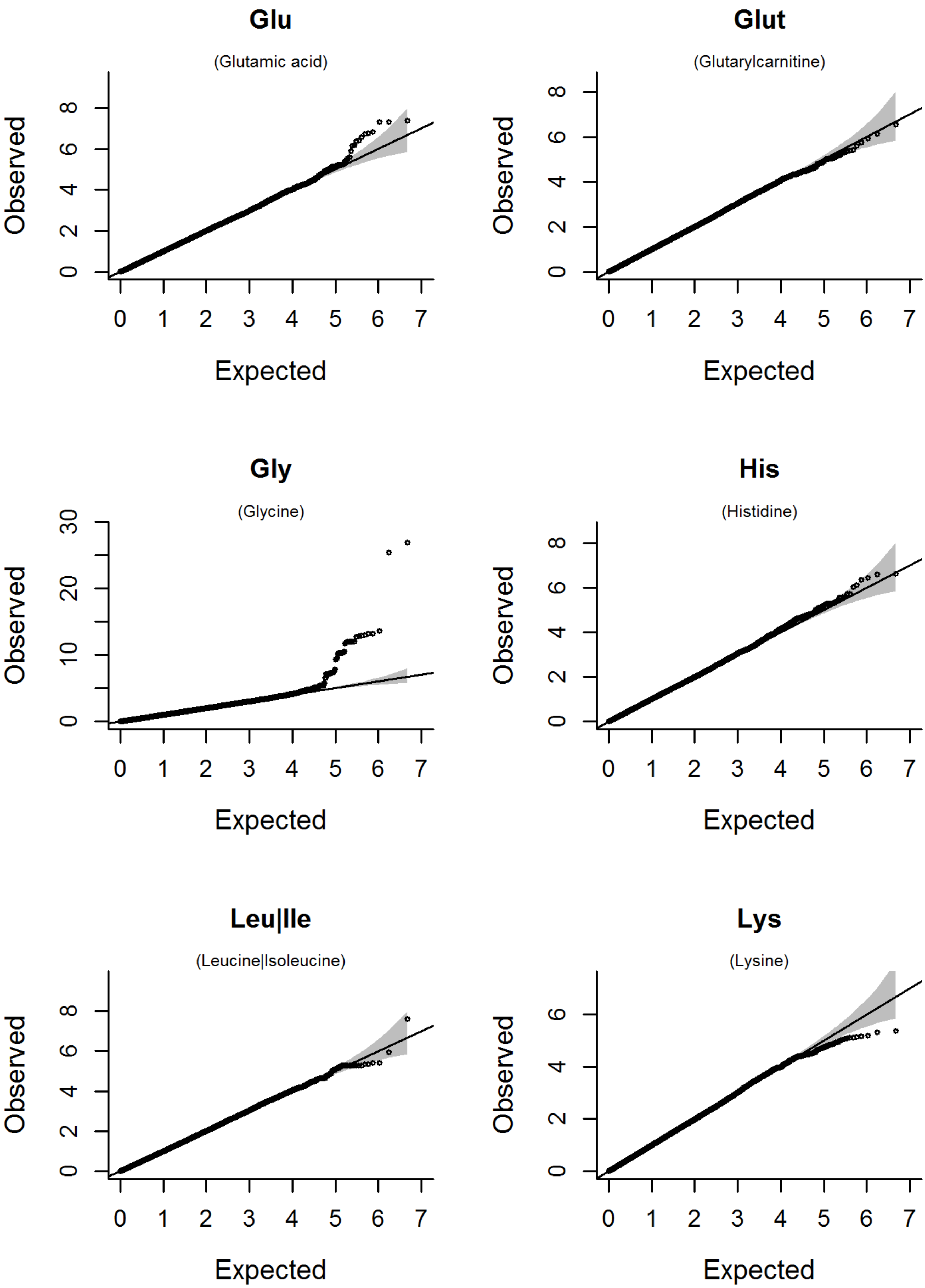
Glu
(Glutamic acid)
Glut
(Glutarylcarnitine)
Gly
(Glycine)
His
(Histidine)
Leu|Ile
(Leucine|Isoleucine)
Lys
(Lysine)
Observed
Expected

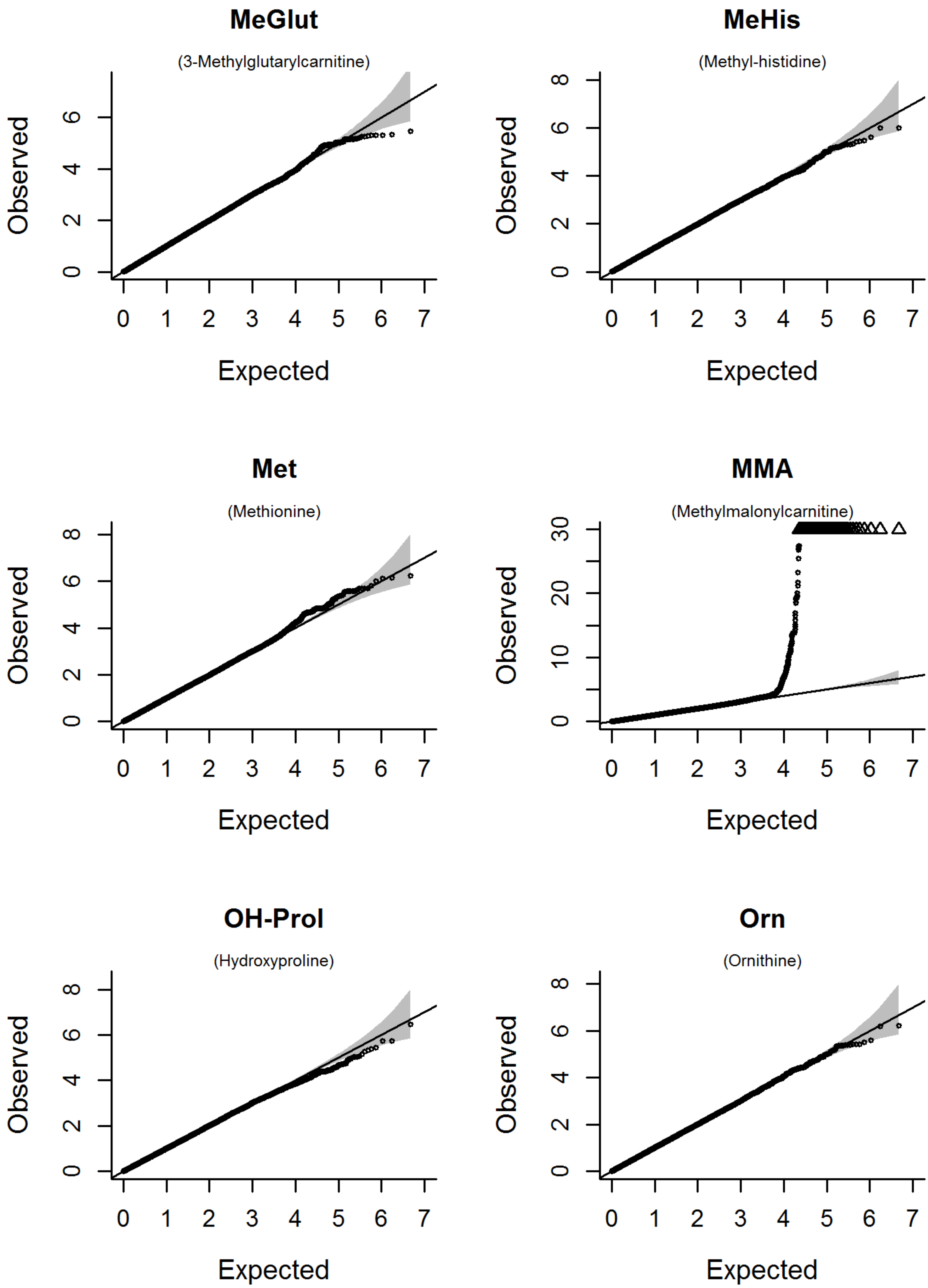
MeGlut
(3-Methylglutarylcarnitine)
Observed
Expected
0
2
4
6
0
1
2
3
4
5
6
7
MeHis
(Methyl-histidine)
Observed
Expected
0
2
4
6
8
0
1
2
3
4
5
6
7
Met
(Methionine)
Observed
Expected
0
2
4
6
8
0
1
2
3
4
5
6
7
MMA
(Methylmalonylcarnitine)
Observed
Expected
0
10
20
30
0
1
2
3
4
5
6
7
OH-Prol
(Hydroxyproline)
Observed
Expected
0
2
4
6
8
0
1
2
3
4
5
6
7
Orn
(Ornithine)
Observed
Expected
0
2
4
6
8
0
1
2
3
4
5
6
7

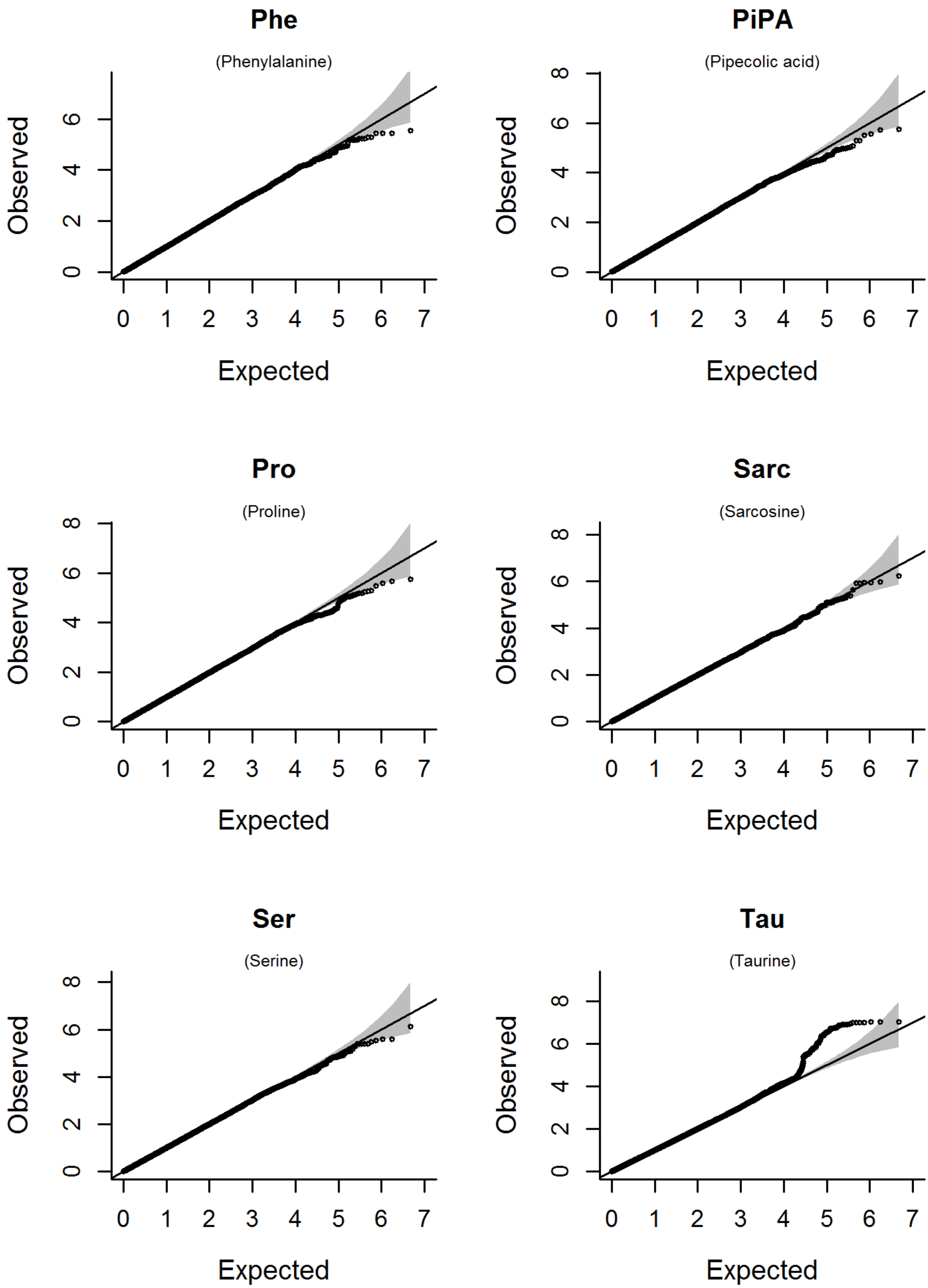
Phe
(Phenylalanine)
PiPA
(Pipecolic acid)
Pro
(Proline)
Sarc
(Sarcosine)
Ser
(Serine)
Tau
(Taurine)
Observed
Expected

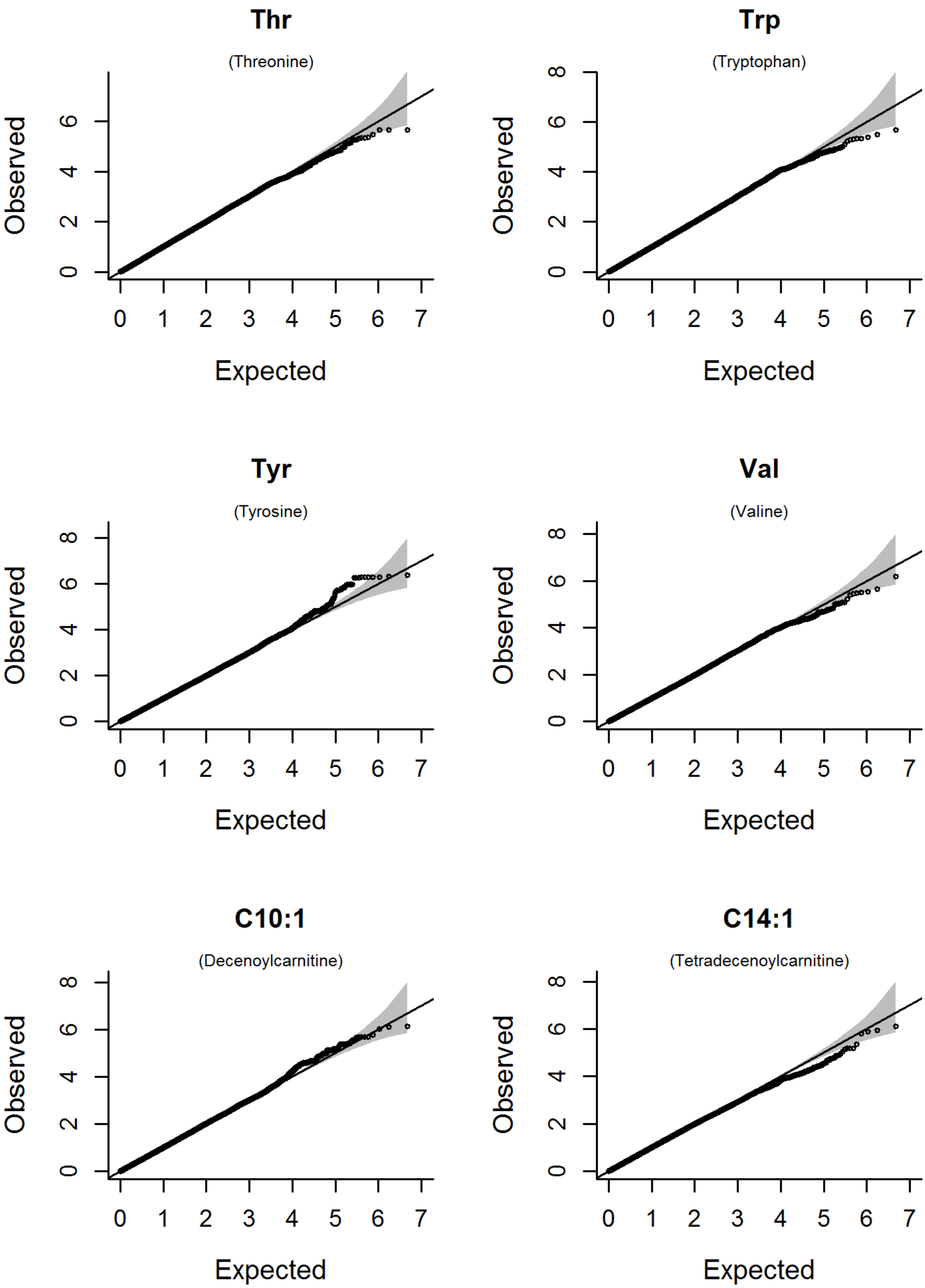

Thr
(Threonine)
Trp
(Tryptophan)
Tyr
(Tyrosine)
Val
(Valine)
C10:1
(Decenoylcarnitine)
C14:1
(Tetradecenoylcarnitine)
Observed
Expected
0 2 4 6 8
0 1 2 3 4 5 6 7

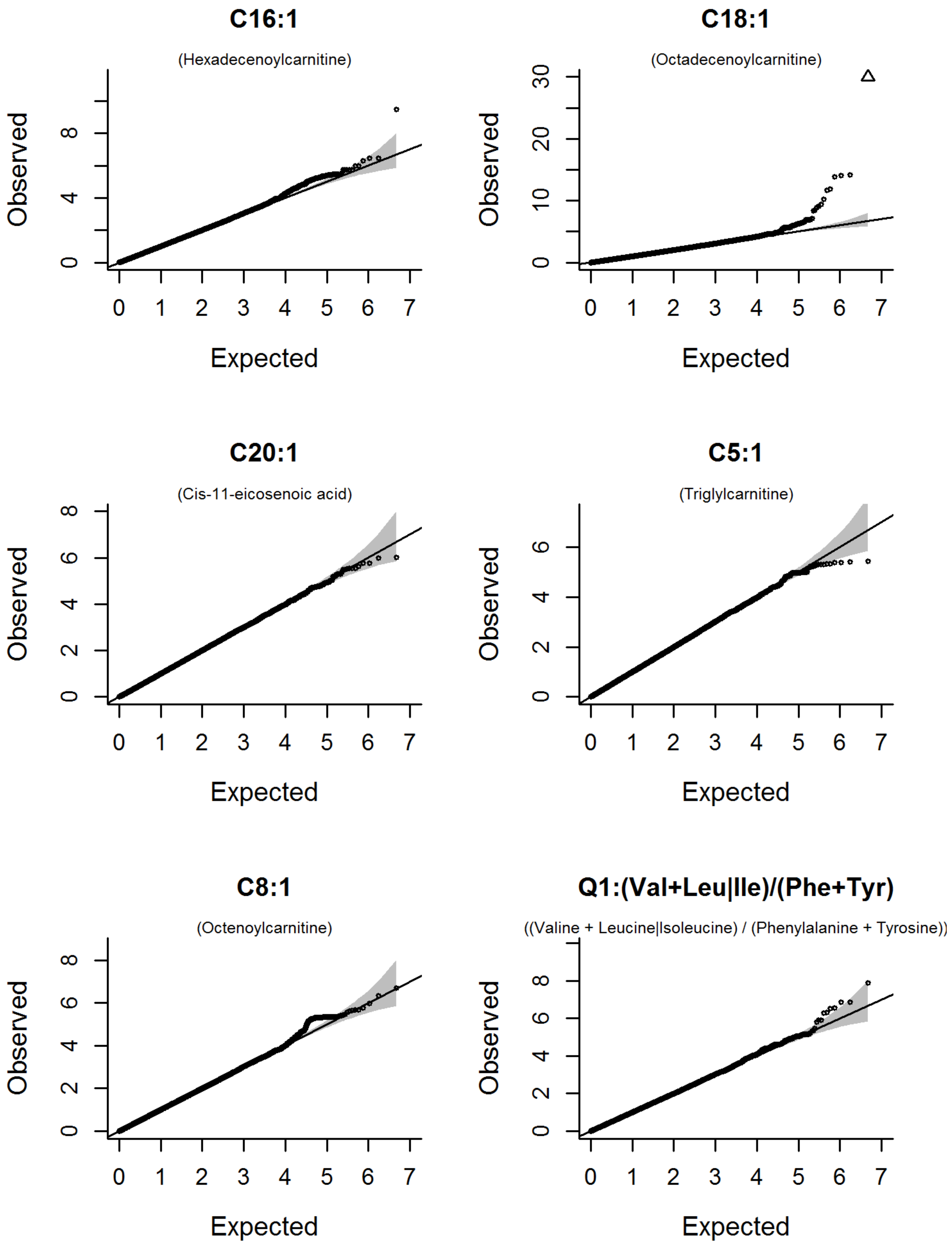
C16:1
(Hexadecenoylcarnitine)
C18:1
(Octadecenoylcarnitine)
C20:1
(Cis-11-eicosenoic acid)
C5:1
(Triglylcarnitine)
C8:1
(Octenoylcarnitine)
Q1:(Val+Leu|Ile)/(Phe+Tyr)
((Valine + Leucine|Isoleucine) / (Phenylalanine + Tyrosine))
Observed
Expected
0 1 2 3 4 5 6 7
0 4 8
0 10 20 30
0 2 4 6 8
0 2 4 6

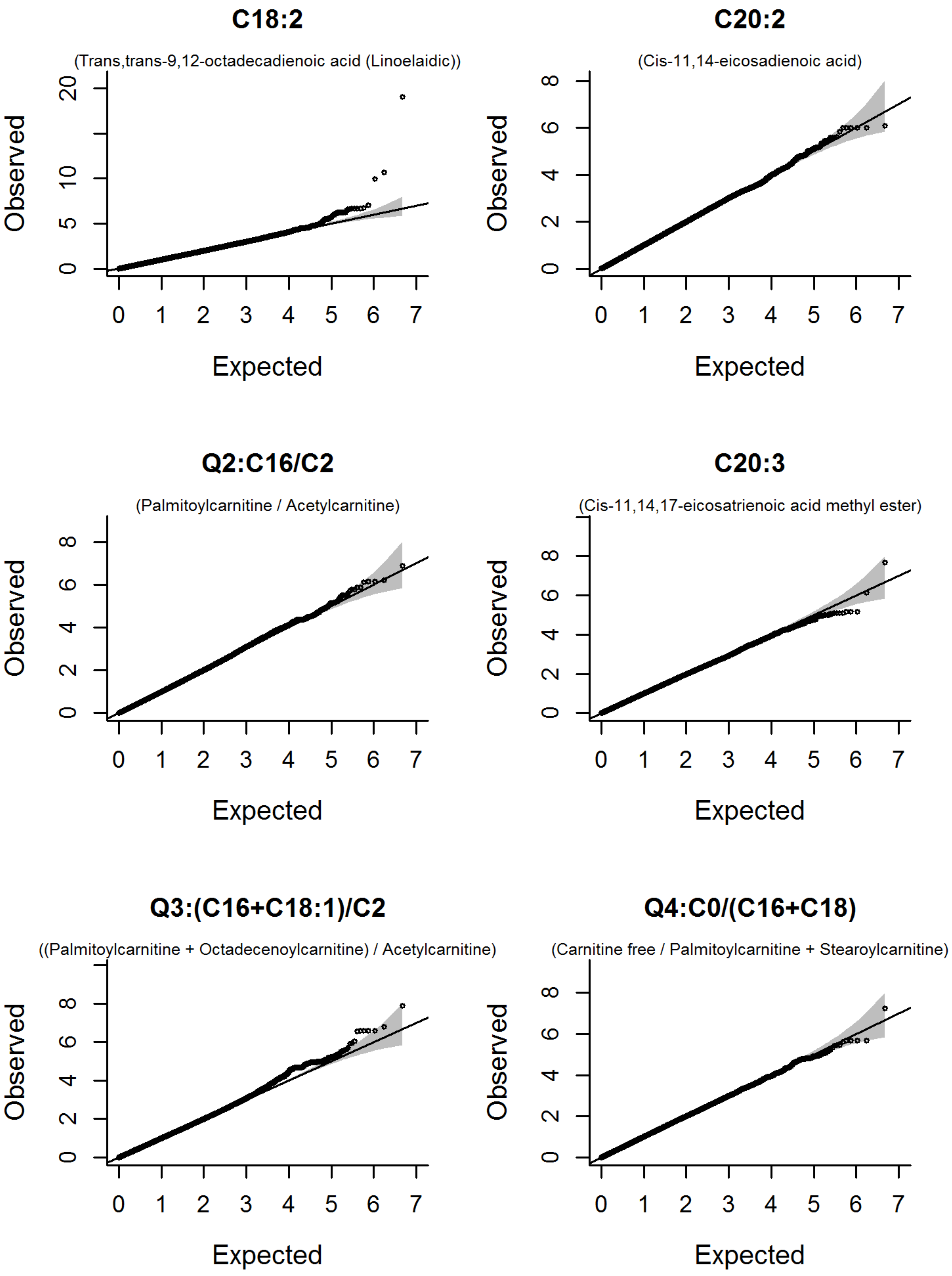
C18:2
(Trans,trans-9,12-octadecadienoic acid (Linoelaidic))
C20:2
(Cis-11,14-eicosadienoic acid)
Q2:C16/C2
(Palmitoylcarnitine / Acetylcarnitine)
C20:3
(Cis-11,14,17-eicosatrienoic acid methyl ester)
Q3:(C16+C18:1)/C2
((Palmitoylcarnitine + Octadecenoylcarnitine) / Acetylcarnitine)
Q4:C0/(C16+C18)
(Carnitine free / Palmitoylcarnitine + Stearoylcarnitine)
Observed
Expected

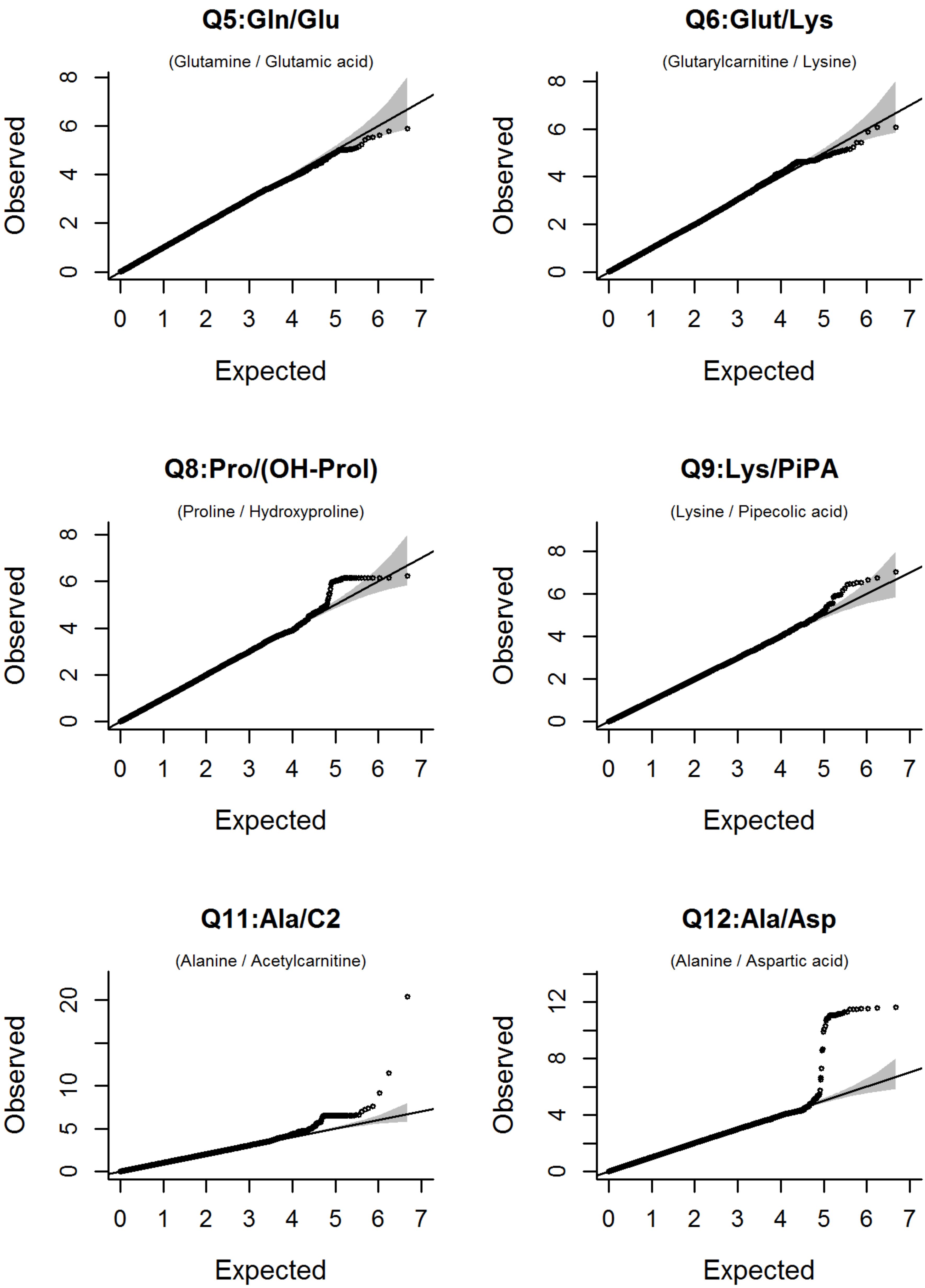
Q5:Gln/Glu
(Glutamine / Glutamic acid)
Q6:Glut/Lys
(Glutarylcarnitine / Lysine)
Q8:Pro/(OH-Prol)
(Proline / Hydroxyproline)
Q9:Lys/PiPA
(Lysine / Pipecolic acid)
Q11:Ala/C2
(Alanine / Acetylcarnitine)
Q12:Ala/Asp
(Alanine / Aspartic acid)
Observed
Expected
0
1
2
3
4
5
6
7
8
10
12
20

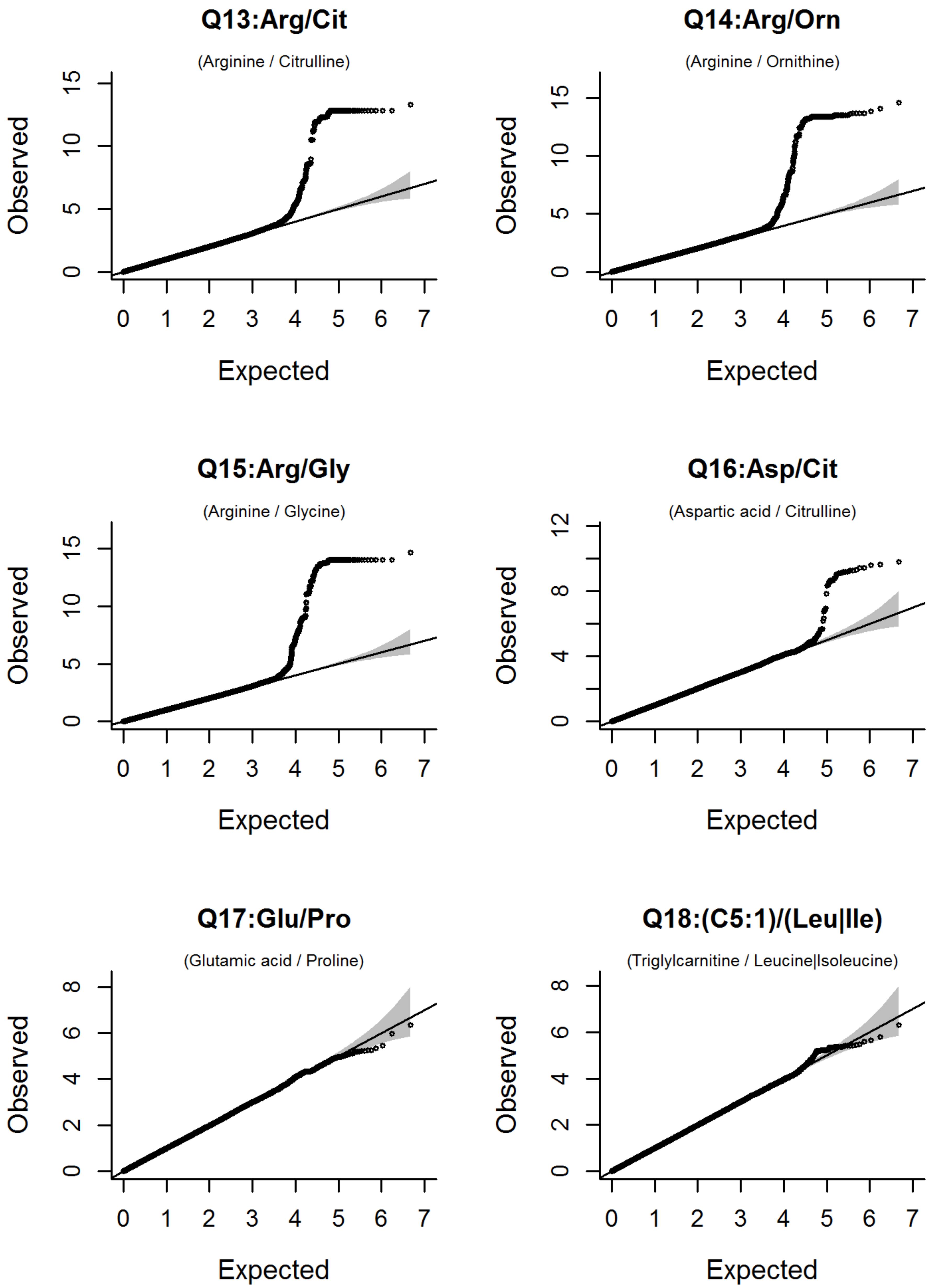
Q13:Arg/Cit
(Arginine / Citrulline)
Q14:Arg/Orn
(Arginine / Ornithine)
Q15:Arg/Gly
(Arginine / Glycine)
Q16:Asp/Cit
(Aspartic acid / Citrulline)
Q17:Glu/Pro
(Glutamic acid / Proline)
Q18:(C5:1)/(Leu|Ile)
(Triglylcarnitine / Leucine|Isoleucine)
Observed
Expected

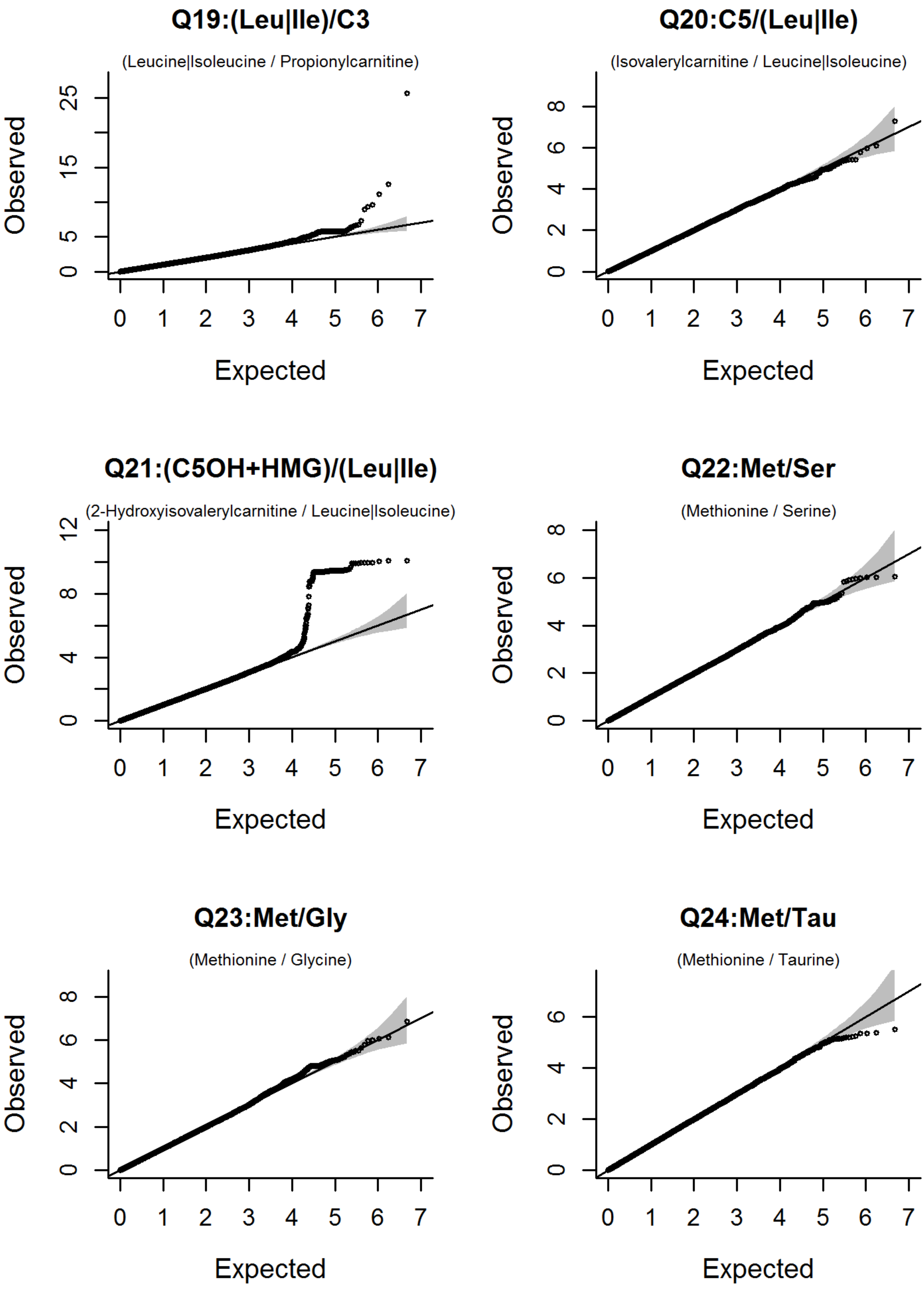
Q19:(Leu|Ile)/C3
(Leucine|Isoleucine / Propionylcarnitine)
Observed
Expected
Q20:C5/(Leu|Ile)
(Isovalerylcarnitine / Leucine|Isoleucine)
Observed
Expected
Q21:(C5OH+HMG)/(Leu|Ile)
(2-Hydroxyisovalerylcarnitine / Leucine|Isoleucine)
Observed
Expected
Q22:Met/Ser
(Methionine / Serine)
Observed
Expected
Q23:Met/Gly
(Methionine / Glycine)
Observed
Expected
Q24:Met/Tau
(Methionine / Taurine)
Observed
Expected

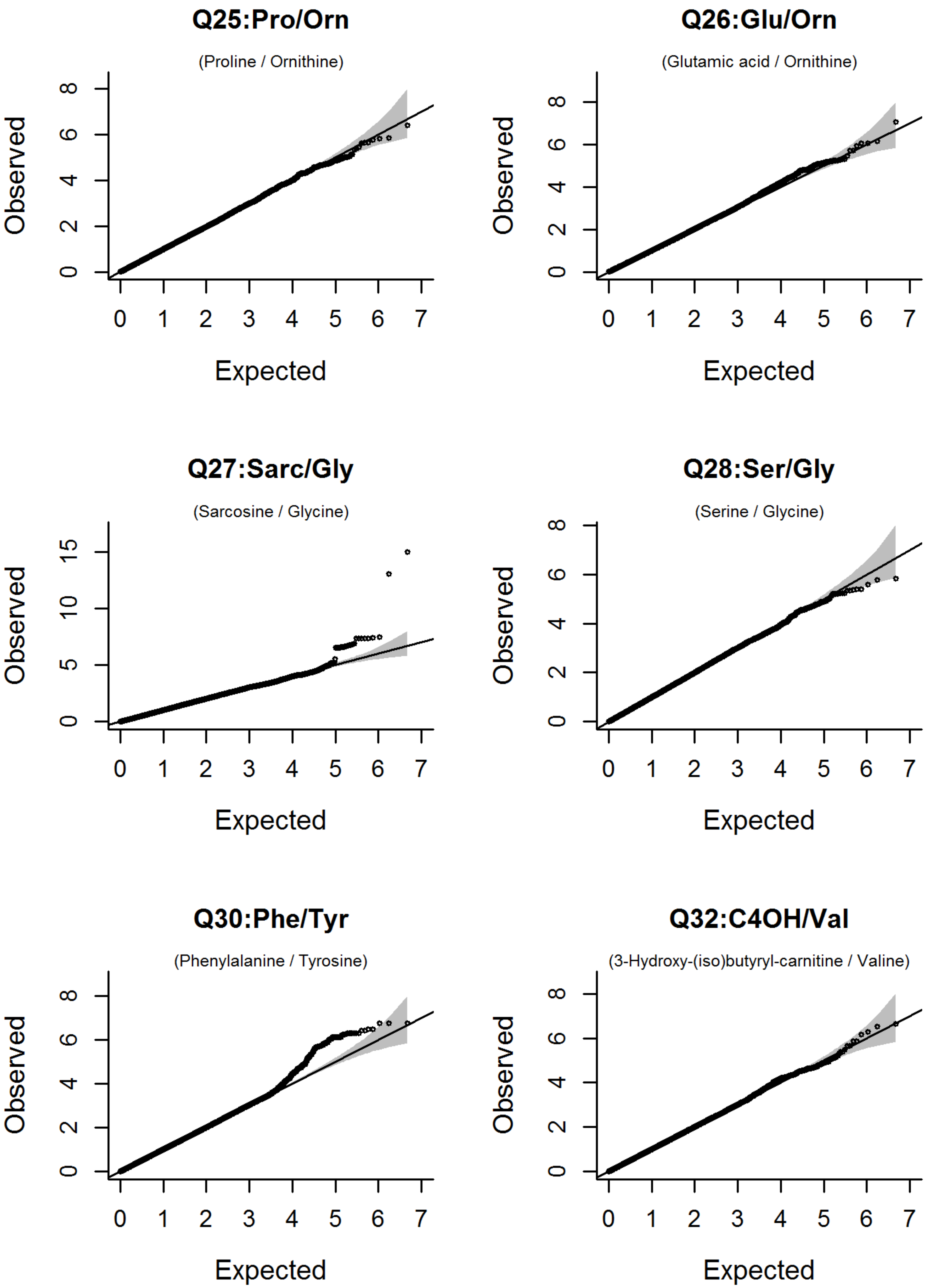

Q25:Pro/Orn
(Proline / Ornithine)
Q26:Glu/Orn
(Glutamic acid / Ornithine)
Q27:Sarc/Gly
(Sarcosine / Glycine)
Q28:Ser/Gly
(Serine / Glycine)
Q30:Phe/Tyr
(Phenylalanine / Tyrosine)
Q32:C4OH/Val
(3-Hydroxy-(iso)butyryl-carnitine / Valine)
Observed
Expected

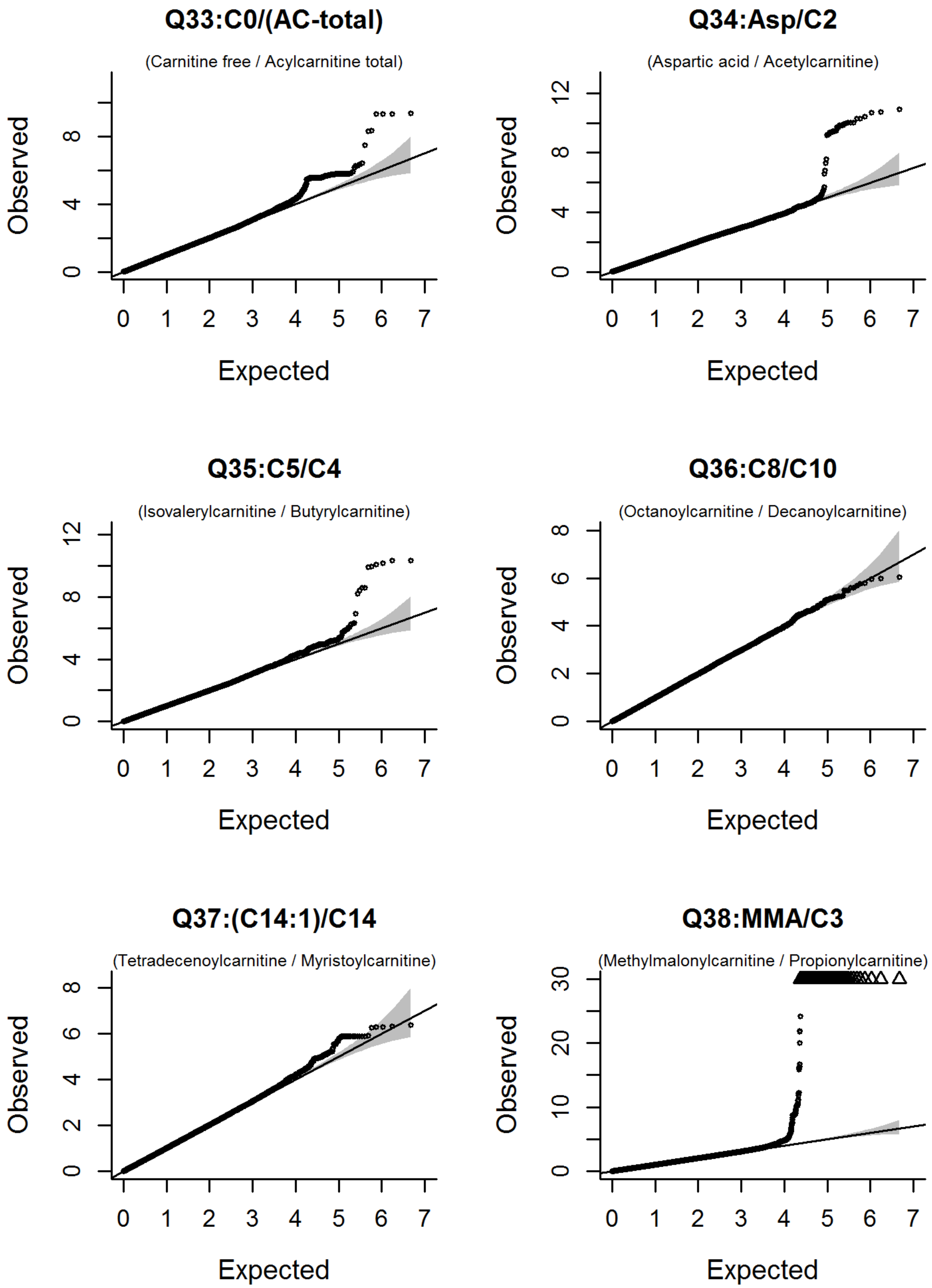
Q33:C0/(AC-total)
(Carnitine free / Acylcarnitine total)
Q34:Asp/C2
(Aspartic acid / Acetylcarnitine)
Q35:C5/C4
(Isovalerylcarnitine / Butyrylcarnitine)
Q36:C8/C10
(Octanoylcarnitine / Decanoylcarnitine)
Q37:(C14:1)/C14
(Tetradecenoylcarnitine / Myristoylcarnitine)
Q38:MMA/C3
(Methylmalonylcarnitine / Propionylcarnitine)
Observed
Expected
