# Supplementary material for: Integration of Genome-Wide SNP Data and Gene-Expression Profiles Reveals Six Novel Loci and Regulatory Mechanisms for Amino Acids and Acylcarnitines in Whole Blood
Source: PLoS Genet. 2015 Sep 24;11(9):e1005510. doi: 10.1371/journal.pgen.1005510 (PMC4581711; doi:10.1371/journal.pgen.1005510)
Supplement: S7 Fig — We present an interactive html-version of Fig 3. Each point represents an eQTL. Test statistics of each eQTL are available as tooltip. For clarity, on chromosome 15 only the strongest cis-eQTL is shown. (HTML) [file pgen.1005510.s007.html]

**Results of eQTL analysis of mQTL hits in LIFE Leipzig Heart**
