# Supplementary material for: Integration of Genome-Wide SNP Data and Gene-Expression Profiles Reveals Six Novel Loci and Regulatory Mechanisms for Amino Acids and Acylcarnitines in Whole Blood
Source: PLoS Genet. 2015 Sep 24;11(9):e1005510. doi: 10.1371/journal.pgen.1005510 (PMC4581711; doi:10.1371/journal.pgen.1005510)
Supplement: S1 Text — (PDF) [file pgen.1005510.s020.pdf]

Material S1: Extended Methods and Discussion of Mendelian Randomization analysis

Here, we explain and discuss our Mendelian Randomization analysis in more detail. For this purpose, we follow Lawlor et al.

In our situation, SNPs of identified association triangles serve as instrumental variables, corresponding regulated gene-expression as exposure and the metabolite concentration as outcome. We want to investigate whether the gene-expression has a causal effect on metabolite levels. However, a number of known and unknown covariables potentially confound this association (see figure below).

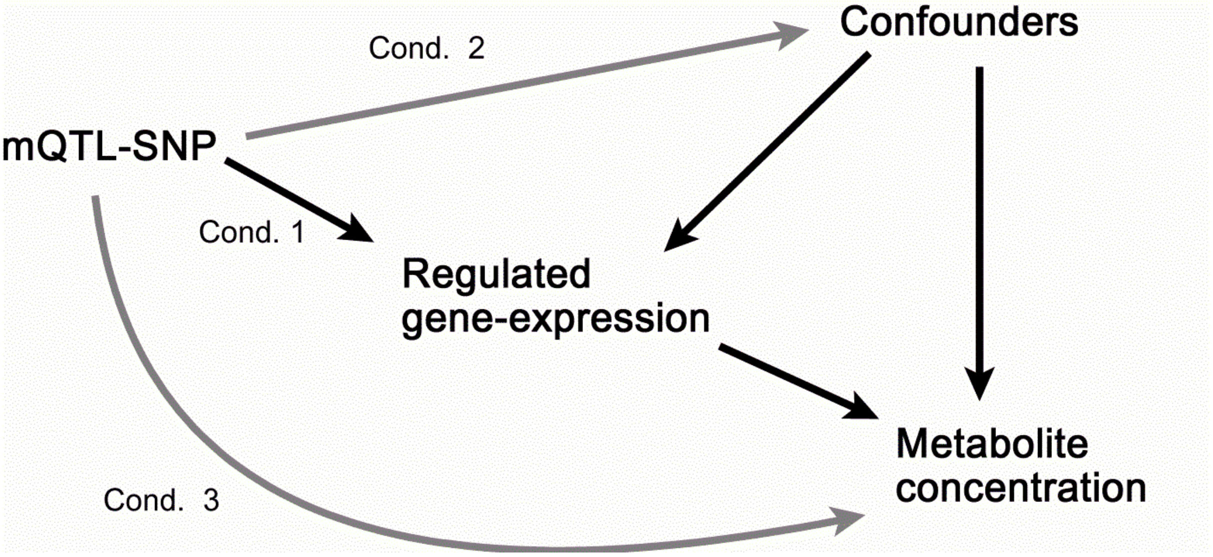

According to Lawlor et al., the assumptions of MR in our situation read as follows:

Condition 1: The mQTL-SNP is associated with the gene-expression.

Condition 2: The mQTL-SNP is not associated with factors confounding the association of gene-expression and metabolite concentration.

Condition 3: There is no association of the mQTL-SNP with the metabolite concentration conditioned on the gene-expression and the confounders. In other words, there is no direct effect of the mQTL-SNP on the metabolite concentration and there are no confounders inducing such a relationship.

The first condition is relatively easy to establish since usually, the association is represented by a strong cis-effect which is functionally plausible.

The second condition is less obvious since in table S8 we showed a number of pleiotropic effects of our mQTL-hits. Therefore, in our regression analysis we controlled for a number of covariables known to affect gene-expression or metabolite levels. By this approach, we avoided potential biases due to violation of condition 2 as much as possible. However, one cannot completely rule out the existence of other confounders with a relevant effect on gene-expression and metabolite levels.

The third condition is the most serious one since mQTL-SNPs were selected on the basis of strong associations with metabolite concentrations. These associations might also be exaggerated due to winners curse effects. To avoid this possible violation of MR conditions, we extended the method by residualizing metabolite concentrations for remaining direct effects of the mQTL-SNP.

According to Lawlor et al., genetic variants in LD with the mQTL hits could also induce spurious associations. They recommended testing different genetic variants for MR analysis which we did by analyzing all independent top-variants at a locus.

Reference: Lawlor DA, Harbord RM, Sterne, Jonathan A C, Timpson N, Davey Smith G (2008) Mendelian randomization: using genes as instruments for making causal inferences in epidemiology. Statistics in medicine 27 (8): 1133–1163.
